# (*E*)-*N'*-Arylidene-2-(4-oxoquinazolin-4(3*H*)-yl) acetohydrazides: Synthesis and evaluation of antitumor cytotoxicity and caspase activation activity

**DOI:** 10.1080/14756366.2018.1555536

**Published:** 2019-01-17

**Authors:** Le Cong Huan, Cao Viet Phuong, Le Cong Truc, Vo Nguyen Thanh, Hai Pham-The, Le-Thi-Thu Huong, Nguyen Thi Thuan, Eun Jae Park, A Young Ji, Jong Soon Kang, Sang-Bae Han, Phuong-Thao Tran, Nguyen-Hai Nam

**Affiliations:** a Pharmaceutical Chemistry, Hanoi University of Pharmacy, Hanoi, Vietnam;; b School of Medicine and Pharmacy, Vietnam National University, Hanoi, Vietnam;; c College of Pharmacy, Chungbuk National University, Cheongju, Republic of Korea;; d Bio-Evaluation Center, Korea Research Institute of Bioscience and Biotechnology, Cheongju, Republic of Korea

**Keywords:** Acetohydrazides, quinazolin-4(3H)-one, cytotoxicity, caspase activation

## Abstract

In our search for novel small molecules activating procaspase-3, we have designed and synthesised a series of novel acetohydrazides incorporating quinazolin-4(3*H*)-ones (**5, 6, 7)**. Biological evaluation revealed eight compounds with significant cytotoxicity against three human cancer cell lines (SW620, colon cancer; PC-3, prostate cancer; NCI-H23, lung cancer). The most potent compound **5t** displayed cytotoxicity up to 5-fold more potent than 5-FU. Analysis of structure-activity relationships showed that the introduction of different substituents at C-6 position on the quinazolin-4(3*H*)-4-one moiety, such as 6-chloro or 6-methoxy potentially increased the cytotoxicity of the compounds. In term of caspase activation activity, several compounds were found to exhibit potent effects, (e.g. compounds **7 b**, **5n**, and **5l**). Especially, compound **7 b** activated caspases activity by almost 200% in comparison to that of PAC-1. Further docking simulation also revealed that this compound potentially is a potent allosteric inhibitor of procaspase-3.

## Graphical Abstract

**Figure:**
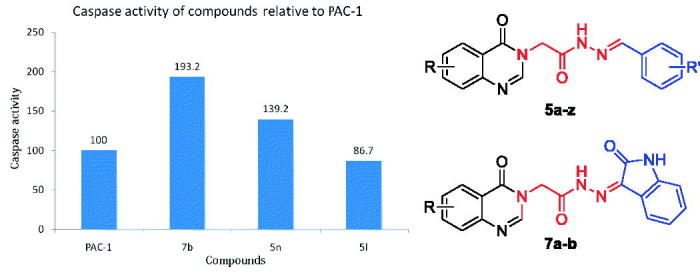

## 1. Introduction

Cancer is one of two leading killers worldwide currently. The primary cause of cancer development and progression is the dysregulation of apoptosis^1^. Compounds such as p53 disruptors (tenovin-1)^2^ and inhibitors of XIAP (GDC-0152)^3^ or Bcl-2 (ABT-199)^4^ act directly on proteins in the apoptotic pathway to induce apoptosis and lead to the death of cancer cells. Caspases are a family of cysteine proteases enzymes important for maintaining homeostasis through regulating cell death. A number of studies have demonstrated that direct activation of procaspase-3 by small molecules could have advantages over the above apoptosis-inducing compounds, because procaspase-3 is found overexpressed in various human tumours, including those of colon cancer^5^, lung cancer^6^, melanoma^7^, hepatoma^8^, breast cancer^9^, lymphoma^10^, and neuroblastoma^11^.

**Figure 1.**
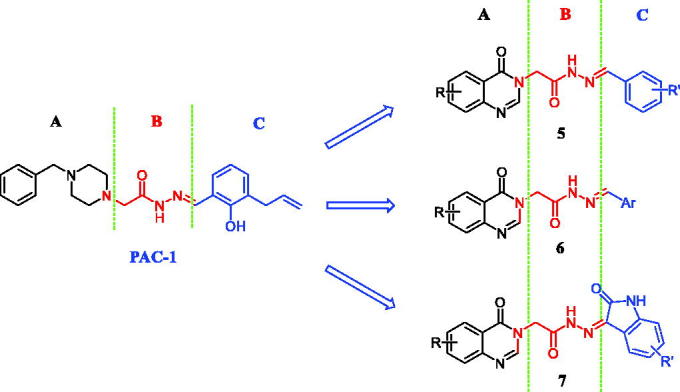
Structure of PAC-1 and rational design of novel (*E*)-*N'*-arylidene-2–(4-oxoquinazolin-4(3*H*)-yl)acetohydrazides.

Despite the significant potential role of caspases in cancer pathology^12^, very few caspase-specific activators have been developed thus far^5^ ^,^ ^13^ ^,^ ^14^. PAC-1, the first procaspase activating compound (Figure 1), is among the first procaspase activating compounds reported in recent years which shows promising *in vivo* antitumor activity profile^5^. Studies on the structure-activity relationships of PAC-1 and other related compounds pointed out that the acyl hydrazone moiety (B-region, Figure 1) plays an important role for their broad bioactivity, owing to their ability to form stable complexes with zinc^15^ ^,^ ^16^.

Quinazolin-4(3*H*)-one is a common scaffold found in many diverse biological compounds (e.g. luotonin A, rutaecarpine, tryptanthrin, chloroqualone, and alloqualone). The quinazoline heterocyclic system, in particular, is also present as a core structure in a number of tyrosine kinase inhibitors and anticancer agents (e.g. gefitinib, erlotinib) ^17^. Based on the pharmacophoric structure of PAC-1 and related acylhydrazone procaspase activators, and the biological importance of quinazolin-4(3*H*)-one scaffold, we have designed three series of novel acyl hydrazones (Figure 1). In the designed compounds, the 4-benzylpiperazin-1-yl part of PAC-1 (A-region) was replaced by the quinazolin-4(3*H*)-one heterocycle system. The phenyl ring of PAC-1 (C-region) was replaced by different substituted phenyl, heteroaryl rings or isatins. The acylhydrazone moiety (B-region) serves as a linker between A- and C-regions was kept unchanged. This paper describes the results from synthesis, biological evaluation and docking studies of the designed compounds.

## 2. Material and methods

### 2.1. Chemistry

Thin layer chromatography which was performed using Whatman^®^ 250 µm Silica Gel GF Uniplates and visualised under UV light at 254 nm, was used to check the progress of reactions and preliminary evaluation of compounds’ homogeneity. Melting points were measured using a Gallenkamp Melting Point Apparatus (LabMerchant, London, United Kingdom) and are uncorrected. Purification of compounds was carried out using crystallization methods and/or open silica gel column flash chromatography employing Merck silica gel 60 (240 to 400 mesh) as stationary phase. Nuclear magnetic resonance spectra (^1^H NMR) were recorded on a Bruker 500 MHz spectrometer with DMSO-*d*_6_ as solvent unless otherwise indicated. Tetramethylsilane was used as an internal standard. Chemical shifts are reported in parts per million (ppm), downfield from tetramethylsilane. Mass spectra with different ionisation modes including electron ionisation (EI), Electrospray ionisation (ESI), were recorded using PE Biosystems API2000 (Perkin Elmer, Palo Alto, CA, USA) and Mariner^®^ (Azco Biotech, Inc. Oceanside, CA, USA**)** mass spectrometers, respectively. The elemental (C, H, N) analyses were performed on a Perkin Elmer model 2400 elemental analyser. All reagents and solvents were purchased from Aldrich or Fluka Chemical Corp. (Milwaukee, WI, USA) or Merck unless noted otherwise. Solvents were used directly as purchased unless otherwise indicated.

**Scheme 1.**
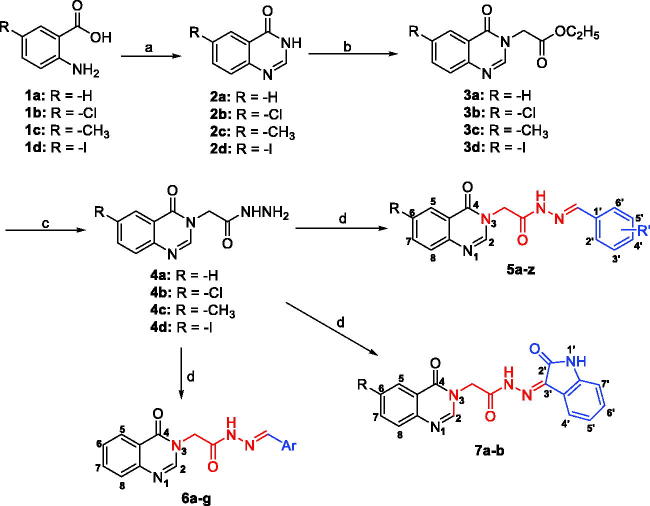
Synthesis of acetohydrazides incorporating quinazolin-4(3*H*)-one (**5, 6, 7**). *Reagents and conditions:* (a) H_2_N-CHO, 120 °C, 3 h; (b) ethyl chloroacetate, KI, K_2_CO_3_, acetone, 60 °C, 3.5 h; (c) N_2_H_4_.H_2_O, EtOH, reflux; (d) Ar-CHO or isatin der., AcOH conc., EtOH, reflux.

The synthesis of acetohydrazides incorporating quinazolin-4(3*H*)-one (**5**, **6**, **7**) was carried out as illustrated in Scheme 1. Details are described below.

The mixtures of anthranilic acid (**1a**) or 5-substituted-2-aminobenzoic acid (**1b–d**) (1 mmol) and formamide (3 mmol) were stirred at 120 °C for 3 h. Upon completion, the resulting mixtures were cooled, poured into ice-cold water, the light brown solids were formed, filtered, and washed with water (3 times) and dried to give quinazoline-4(3*H*)-on derivatives (**2a–d**), which were used for the next step without further purification.

To a respective solution of quinazoline-4(3*H*)-on derivatives (**2a–d**) (1 mmol) in acetone (10 ml) were added K_2_CO_3_ (206.9 mg, 1.5 mmol). The resulting mixtures were stirred at 80 °C for 30 min, then KI (16.6 mg, 0.1 mmol) was added. After stirring for further 15 min, 0.13 ml of ethyl chloroacetate (1.2 mmol) were dropped slowly into the mixtures. The reaction mixtures were again stirred at 60 °C for 3 h. After completion of the reaction, the resulting mixtures were cooled, poured into ice-cold water. The brown solids were formed, filtered and dried to give the ethyl 2–(4-oxoquinazolin-3-(4*H*)-yl)acetate derivatives **3a–d**.

To a respective solution of compounds **3a–d** (0.5 mmol) in ethanol (10 ml) was added 0.12 ml of hydrazine monohydrate (2.5 mmol) slowly. The mixture was stirred at room until the starting material consumed. The white precipitates formed were filtered and washed with cold ethanol (3 times), the off-white solids (**4a–d**) were collected, dried under vacuum and carried on to the next step without further purification.

The acetohydrazides **4a–d** (0.5 mmol) were dissolved in ethanol (20 ml), 2 drops of concentrated acetic acid, followed by benzaldehyde or isatine derivatives (1.0 mmol) were added. The mixtures were refluxed until the reaction completed. The precipitates formed were filtered and washed with ethanol (3 times), the off-white solid products were collected, dried under vacuum and re-crystalised in ethanol or column chromatography (MeOH:DCM) to obtain the desired product **5a–z**, **6a–g**, **7a**–**b**.

#### (E)-N'-Benzylidene-2–(4-oxoquinazolin-3(4H)-yl)acetohydrazide (5a)

White solid; Yield: 60%. mp: 179.7–181.5 °C. *R_f_*=0.53 (DCM: MeOH = 14: 1). *IR (KBr, cm^−1^):* 3506 (NH); 3236 (N = C–H aromatic); 3060, 2990 (CH, aren); 2830 (CH_2_); 1719, 1680, 1531 (C = O, C = N); 1608 (C = C). *^1^H-NMR (500 MHz, DMSO-d_6_, ppm)*: *δ* 11.90, 11.84 (∼26%, 74%) (s, 1H, CONH); 8.39 (s, 1H, H_2_); 8.24, 8.07 (∼22%, 78%) (s, 1H, N = CH); 8.17 (d, *J* = 7.5 Hz, 1H, H_5_); 7.87 (t, *J* = 7.5 Hz, 1H, H_7_); 7.76–7.71 (m, 3H, H_2′_, H_6′_, H_8_); 7.58 (t, *J* = 7.5 Hz, 1H, H_6_); 7.48–7.45 (m, 3H, H_3′_, H_4′_, H_5′_); 5.24, 4.80 (∼23%, 77%) (s, 2H, NCH_2_CO). 13*C NMR (125 MHz, DMSO-d_6_, ppm): δ* 168.3 (CONH), 160.3 (C = O), 148.6 (C_8_=C–N = C_2_), 148.1 (C_2_), 144.3 (N=CH), 134.5 (C_7_), 133.9 (C_1′_), 130.1 (C_4′_), 128.9 (C_2′_, C_6′_), 127.2 (C_6_), 127.1 (C_8_), 126.9 (C_3′_, C_5′_), 126.1 (C_5_), 121.5 (C_5_=C–C = O), 47.0 (NCH_2_CO). MS (ESI) *m/z* 307.9 [M + H]^+^. Anal. Calcd. For C_17_H_14_N_4_O_2_ (306.1117): C, 66.66; H, 4.61; N, 18.29. Found: C, 66.63; H, 4.64; N, 18.32.

#### (E)-N'-(2-Chlorobenzylidene)-2–(4-oxoquinazolin-3(4H)-yl)acetohydrazide (5b)

White solid; Yield: 44%. mp: 180.0–182.0 °C. *R_f_*=0.55 (DCM: MeOH = 14: 1). *IR (KBr, cm^−1^):* 3506 (NH); 3240 (N = C–H aromatic); 3059, 2987 (CH, aren); 1707, 1685, 1558 (C = O, C = N); 1608 (C = C); 777 (C–Cl). *^1^H-NMR (500 MHz, DMSO-d_6_, ppm)*: *δ* 12.09, 11.97 (∼22%, 78%) (s, 1H, CONH); 8.63, 8.45 (∼22%, 78%) (s, 1H, N = CH); 8.38, 8.37 (s, 1H, H_2_); 8.16 (dd, *J* = 8.0 Hz, 1.5 Hz, 1H, H_5_); 8.04, 7.94 (∼77%, 23%) (dd, *J* = 7.0 Hz, 2.0 Hz, 1H, H_6′_), 7.85 (td, *J* = 8.0 Hz, 1.5 Hz, 1H, H_7_); 7.72 (d, *J* = 8.0 Hz, 1H, H_8_); 7.56 (t, *J* = 7.5 Hz, 1H, H_6_); 7.53, 7.52 (dd, *J* = 7.0 Hz, 1.5 Hz, 1H, H_3′_); 7.47–7.42 (m, 2H, H_4′_, H_5′_); 5.24, 4.81 (∼80%, 20%) (s, 2H, NCH_2_CO). 13*C NMR (125 MHz, DMSO-d_6_, ppm): δ* 168.4 (CONH), 160.2 (C = O), 148.5 (C_8_=C–N = C_2_), 148.0 (C_2_), 140.3 (N=CH), 134.4 (C_7_), 133.0 (C_1′_), 131.4 (C_2′_), 131.1 (C_4′_), 128.9 (C_3′_), 127.6 (C_6′_), 127.2 (C_6_), 127.0 (C_8_), 126.8 (C_5′_), 125.9 (C_5_), 121.4 (C_5_=C–C = O), 46.9 (NCH_2_CO). MS (ESI) *m/z* 340.9 [M + H]^+^. Anal. Calcd. For C_17_H_13_ClN_4_O_2_ (340.0727): C, 59.92; H, 3.85; N, 16.44. Found: C, 59.90; H, 3.87; N, 16.46.

#### (E)-N'-(2-Nitrobenzylidene)-2–(4-oxoquinazolin-3(4H)-yl)acetohydrazide (5c)

White solid; Yield: 56%. mp: 182.1–183.3 °C. *R_f_*=0.60 (DCM: MeOH = 14: 1). *^1^H-NMR (500 MHz, DMSO-d_6_, ppm)*: *δ* 11.93 (s, 1H, CONH); 8.65, 8.46 (∼23%, 77%) (s, 1H, N = CH); 8.38, 8.37 (s, 1H, H_2_); 8.16 (dd, *J* = 7.5 Hz, 1.0 Hz, 1H, H_5_); 8.13, 8.10 (dd, *J* = 8.0 Hz, 1.5 Hz, 1H, H_6′_); 8.08, 8.03 (∼78%, 22%) (dd, *J* = 8.5 Hz, 1.5 Hz, 1H, H_3′_); 7.87 (td, *J* = 8.5 Hz, 1.5 Hz, 1H, H_5′_); 7.82 (t, *J* = 7.5 Hz, 1H, H_7_); 7.72 (d, *J* = 8.0 Hz, 1H, H_8_); 7.69 (td, *J* = 7.5 Hz, 1.5 Hz, 1H, H_6_); 7.58 (td, *J* = 8.0 Hz, 1.0 Hz, 1H, H_4′_); 5.23, 4.81 (∼78%, 22%) (s, 2H, NCH_2_CO). 13*C NMR (125 MHz, DMSO-d_6_, ppm): δ* 168.5 (CONH), 160.2 (C = O), 148.5 (C_8_=C–N = C_2_), 148.0 (C_2_), 143.0 (C_2′_), 139.9 (N=CH), 134.5 (C_7_), 133.6 (C_5′_), 130.7 (C_4′_), 128.2 (C_6′_), 128.0 (C_1′_), 127.2 (C_6_), 127.1 (C_8_), 126.0 (C_5_), 124.6 (C_3′_), 121.4 (C_5_=C–C = O), 46.9 (NCH_2_CO). Anal. Calcd. For C_17_H_13_N_5_O_4_ (351.0968): C, 58.12; H, 3.73; N, 19.93. Found: C, 58.16; H, 3.70; N, 19.96.

#### (E)-N'-(3-Chlorobenzylidene)-2-(4-oxoquinazolin-3(4H)-yl)acetohydrazide (5d)

White solid; Yield: 58%. mp: 184.0–185.0 °C. *R_f_*=0.55 (DCM: MeOH = 14: 1). *^1^H-NMR (500 MHz, DMSO-d_6_, ppm)*: *δ* 11.91 (s, 1H, CONH); 8.37 (s, 1H, H_2_); 8.16–8.15 (m, 1H, H_5_); 8.05 (s, 1H, N = CH); 7.85–7.82 (m, 2H, H_7_, H_2′_); 7.71–7.69 (m, 2H, H_6′_, H_8_); 7.57–7.49 (m, 3H, H_4′_, H_6_, H_5′_); 5.25, 4.80 (∼80%, 20%) (s, 2H, NCH_2_CO). 13*C NMR (125 MHz, DMSO-d_6_, ppm): δ* 168.4 (CONH), 160.3 (C = O), 148.5 (C_8_=C–N = C_2_), 148.0 (C_2_), 145.7 (N=CH), 142.7 (C_1′_), 136.1 (C_3′_), 134.5 (C_7_), 133.7 (C_4′_), 130.7 (C_5′_), 129.7 (C_6′_), 127.2 (C_6_), 127.1 (C_8_), 126.0 (C_5_), 125.8 (C_2′_), 121.4 (C_5_=C–C = O), 47.1 (NCH_2_CO). Anal. Calcd. For C_17_H_13_ClN_4_O_2_ (340.0727): C, 59.92; H, 3.85; N, 16.44. Found: C, 59.95; H, 3.83; N, 16.41.

#### (E)-N'-(4-Chlorobenzylidene)-2-(4-oxoquinazolin-3(4H)-yl)acetohydrazide (5e)

White solid; Yield: 31%. mp: 184.2–185.4 °C. *R_f_*=0.56 (DCM: MeOH = 14: 1). *^1^H-NMR (500 MHz, DMSO-d_6_, ppm)*: *δ* 11.96, 11.89 (∼26%, 74%) (s, 1H, CONH); 8.34 (s, 1H, H_2_); 8.24, 8.07 (∼22%, 78%) (s, 1H, N = CH); 8.17 (d, *J* = 7.5 Hz, 1H, H_5_); 7.86–7.92 (m, 3H, H_2′_, H_6′_, H_7_); 7.78 (d, *J* = 8.5 Hz, 1H, H_8_); 7.59 (t, *J* = 7.5 Hz, 1H, H_6_); 7.53 (d, *J* = 8.5 Hz, 2H, H_3’_, H_4’_);5.24, 4.81 (∼23%, 77%) (s, 2H, NCH_2_CO). 13*C NMR (125 MHz, DMSO-d_6_, ppm): δ* 168.8 (CONH), 161.1 (C = O), 149.0 (C_8_=C–N = C_2_), 148.6 (C_2_), 144.3 (N=CH), 136.5 (C_4’_), 135.0 (C_7_), 133.5 (C_1′_), 130.0 (C_2′_, C_6′_), 129.5 (C_3′_, C_5′_), 127.7 (C_6_), 127.6 (C_8_), 129.0 (C_3′_, C_5′_), 126.5 (C_5_), 121.9 (C_5_=C–C = O), 47.4 (NCH_2_CO). MS (ESI) *m/z* 339.1 [M-H]^−^. Anal. Calcd. For C_17_H_13_ClN_4_O_2_ (340.0727): C, 59.92; H, 3.85; N, 16.44. Found: C, 59.89; H, 3.88; N, 16.47.

#### (E)-N'-(4-Fluorobenzylidene)-2-(4-oxoquinazolin-3(4H)-yl)acetohydrazide (5f)

White solid; Yield: 49%. mp: 181.0–182.0 °C. *R_f_*=0.59 (DCM: MeOH = 14: 1). *^1^H-NMR (500 MHz, DMSO-d_6_, ppm)*: *δ* 11.89, 11.83 (∼22%, 78%) (s, 1H, CONH); 8.38, 8,25 (∼18%, 82%) (s, 1H, H_2_); 8.17, 7.97 (∼81%, 19%) (dd, *J* = 9.0 Hz, 1.0 Hz, 1H, H_5_); 8.73, 8.25 (∼13%, 87%) (s, 1H, N = CH); 7.86 (td, *J* = 8.5 Hz, 1.5 Hz, 2H, H_7_); 7.84, 7.80 (∼78%, 22%) (dd, *J* = 3.5 Hz, 3.0 Hz, 2H, H_2′_, H_6′_); 7.74 (d, *J* = 8.0 Hz, 1H, H_8_); 7.58 (td, *J* = 8.0 Hz, 1.0 Hz, 1H, H_6_); 7.38, 7.31 (∼11%, 81%) (t, *J* = 9.0 Hz, 2H, H_3′_, H_5′_); 5.24, 4.80 (∼79%, 21%) (s, 2H, NCH_2_CO). 13*C NMR (125 MHz, DMSO-d_6_, ppm): δ* 168.7 (CONH), 162.6 (C_4′_), 160.8 (C = O), 149.1 (C_2_), 148.6 (C_8_=C–N = C_2_), 143.6 (N=CH), 135.0 (C_7_), 131.1 (C_1′_), 129.64 (C_2′_), 129.57 (C_6′_), 127.7 (C_6_), 127.6 (C_8_), 126.5 (C_5_), 121.9 (C_5_=C–C = O), 116.6 (C_3′_), 116.5 (C_5′_), 47.5 (NCH_2_CO). MS (ESI) *m/z* 323.2 [M-H]^−^. Anal. Calcd. For C_17_H_13_FN_4_O_2_ (324.1023): C, 62.96; H, 4.04; N, 17.28. Found: C, 62.93; H, 4.07; N, 17.31.

#### (E)-N'-(4-Bromobenzylidene)-2–(4-oxoquinazolin-3(4H)-yl)acetohydrazide (5g)

White solid; Yield: 33%. mp: 181.2–182.4 °C. *R_f_*=0.57 (DCM: MeOH = 14: 1). *^1^H-NMR (500 MHz, DMSO-d_6_, ppm)*: *δ* 11.93, 11.85 (∼22%, 78%) (mestnova) (s, 1H, CONH); 8.36 (s, 1H, H_2_); 8.22, 8.05 (∼24%, 76%) (s, 1H, N = CH); 8.16 (dd, *J* = 7.5 Hz, 1.0 Hz, 1H, H_5_); 7.86 (td, *J* = 7.0 Hz, 1.0 Hz, H_7_); 7.82, 7.66 (∼14%, 86%) (d, *J* = 7.5 Hz, 2H, H_4′_, H_6′_); 7.72 (d, *J* = 8.5 Hz, 1H, H_8_). 7.70 (d, *J* = 7.0 Hz, 2H, H_3′_, H_5′_); 7.57 (t, *J* = 7.5 Hz, 1H, H_6_); 5.23, 4.79 (∼78%, 22%) (s, 2H, NCH_2_CO). 13*C NMR (125 MHz, DMSO-d_6_, ppm): δ* 168.3 (CONH), 160.2 (C = O), 148.5 (C_8_=C–N = C_2_), 148.0 (C_2_), 143.1 (N=CH), 134.5 (C_7_), 133.2 (C_1′_), 132.0 (C_3′_), 131.8 (C_5′_), 130.2 (C_4′_), 128.8 (C_6′_), 127.2 (C_6_), 127.1 (C_8_), 126.0 (C_5_), 123.3 (C_4′_), 121.4 (C_5_=C–C = O), 46.9 (NCH_2_CO). Anal. Calcd. For C_17_H_13_BrN_4_O_2_ (384.0222): C, 53.00; H, 3.40; N, 14.54. Found: C, 52.97; H, 3.43; N, 14.57.

#### (E)-N'-(2-Hydroxybenzylidene)-2-(4-oxoquinazolin-3(4H)-yl)acetohydrazide (5h)

White solid; Yield: 37%. mp: 176.0–177.2 °C. *R_f_*=0.54 (DCM: MeOH = 14: 1). *IR (KBr, cm^−1^):* 3460 (NH); 3066 (OH); 3021 (CH, aren); 2912 (CH, CH_2_); 1697 (C = O); 1608, 1570 (C = C). *^1^H-NMR (500 MHz, DMSO-d_6_, ppm)*: *δ* 12.09, 11.75 (∼34%, 66%) (s, 1H, CONH); 10.94, 10.10 (∼37%, 63%) (s, 1H, 2′-OH); 8.47, 8.39 (s, 1H, N = CH); 8.38 (s, 1H, H_2_); 8.18 (d, *J* = 8 Hz, 1H, H_5_); 7.89 (t, J = 7 Hz, 1H, H_7_); 7.78 (d, *J* = 7.5 Hz, 1H, H_8_); 7.50 – 7.72 (m, 1H, H_6′_); 7.60 – 7.57 (m, 2H, H_6′_, H_6_); 7.32 – 7.26 (m, 1H, H_4′_); 6.94 – 6.88 (m, 2H, H_3′_, H_5′_); 5.22, 4.81 (∼65%, 35%) (s, 2H, NCH_2_CO). 13C-NMR (125 MHz, DMSO), *δ* 168.3 (CONH); 160.7 (C = O); 156.9 (C_2′_); 149.1 (N = CH); 148.6 (C_8_=C–N = C_2_); 142.0 (C_2_); 134.9 (C_4’_); 131.8 (C_6′_); 129.6 (C_7_); 127.8 (C_5_); 127.6 (C_6_); 126.5 (C_8_); 121.9 (C_5_=C–C = O); 120.5 (C_5′_); 119.9 (C_1′_); 116.8 (C_3′_); 47.4 (NCH_2_CO). Anal. Calcd. For C_17_H_14_N_4_O_3_ (322.1066): C, 63.35; H, 4.38; N, 17.38. Found: C, 63.38; H, 4.35; N, 17.41.

#### (E)-N'-(4-Hydroxybenzylidene)-2-(4-oxoquinazolin-3(4H)-yl)acetohydrazide (5i)

White solid; Yield: 43%. mp: 178.2–179.3 °C. *R_f_*=0.55 (DCM: MeOH = 14: 1). *^1^H-NMR (500 MHz, DMSO-d_6_, ppm)*: *δ* 11.64, 11.58 (s, 1H, CONH); 9.90 (s, 1H, OH); 8.37, 8.36 (s, 1H, H_2_); 8.16 (dd, *J* = 8.0, 1.0 Hz, 1H, H_5_); 8.13, 7.97 (s, 1H, N = CH); 7.86 (td, *J* = 7.5 Hz, 1.0 Hz, 1H, H_7_); 7.72 (d, *J* = 8.0 Hz, 1H, H_8_); 7.59–7.53 (m, 3H, H_6_, H_2′_, H_6′_); 6.84, 6.82 (d, *J* = 8.5 Hz, 2H, H_3′_, H_5′_); 5.20, 4.76 (∼78%, 22%) (s, 2H, NCH_2_CO). 13*C NMR (125 MHz, DMSO-d_6_, ppm): δ* 167.8 (CONH), 160.2 (C = O), 159.4 (C–OH), 148.6 (C_8_=C–N = C_2_), 148.1 (C_2_), 144.5 (N=CH), 134.4 (C_7_), 128.6 (C_2′_, C_6′_), 127.2 (C_6_), 127.0 (C_8_), 126.0 (C_5_), 124.9 (C_1′_), 121.4 (C_5_=C–C = O), 115.7 (C_3′_, C_5′_), 46.9 (NCH_2_CO). MS (ESI) *m/z* 323.2 [M + H]^+^. Anal. Calcd. For C_17_H_14_N_4_O_3_ (322.1066): C, 63.35; H, 4.38; N, 17.38. Found: C, 63.33; H, 4.40; N, 17.35.

#### (E)-N'-(4-Methoxybenzylidene)-2-(4-oxoquinazolin-3(4H)-yl)acetohydrazide (5j)

White solid; Yield: 41%. mp: 185.0–186.0 °C. *R_f_*=0.56 (DCM: MeOH = 14: 1). *^1^H-NMR (500 MHz, DMSO-d_6_, ppm)*: *δ* 11.97, 11.73 (∼23%, 77%) (s, 1H, CONH); 8.57, 8.56 (s, 1H, H_2_); 8.23, 8.04 (∼23%, 77%) (s, 1H, N = CH); 8.18, 8.17 (dd, *J* = 8.0 Hz, 1.0 Hz, H_5_); 7.90 (td, *J* = 8.0 Hz, 1.0 Hz, 1H, H_7_); 7.76 (d, *J* = 8.5 Hz, 1H, H_8_); 7.69, 7.65 (d, *J* = 8.0 Hz, 2H, H_2′_, H_6′_); 7.61 (t, *J* = 8.0 Hz, 1H, H_6_); 7.02, 7.01 (d, *J* = 8.5 Hz, 2H, H_3′_, H_5′_); 5.24, 4.82 (∼78%, 22%) (s, 2H, NCH_2_CO); 3.81, 3.80 (s, 3H, OCH_3_). 13*C NMR (125 MHz, DMSO-d_6_, ppm): δ* 167.7 (CONH), 160.9 (C = O), 159.9 (C_4__′_–OCH_3_), 149.0 (C_8_=C–N = C_2_), 147.2 (C_2_), 144.3 (N=CH), 134.7 (C_7_), 128.7 (C_6_), 128.5 (C_2′_, C_6′_), 127.4 (C_8_), 126.4 (C_1′_), 126.2 (C_5_), 121.2 (C_5_=C–C = O), 114.3 (C_3′_, C_5′_), 55.3 (OCH_3_), 47.1 (NCH_2_CO). MS (ESI) *m/z* 337.1 [M + H]^+^.

#### (E)-N'-(2,3-Dihydroxybenzylidene)-2-(4-oxoquinazolin-3(4H)-yl)acetohydrazide (5k)

White solid; Yield: 36%. mp: 172.1-173.6 °C. *R_f_*=0.57 (DCM: MeOH = 14: 1). *IR (KBr, cm^−1^):* 3564 (NH); 3452 (OH); 3259 (CH, aren); 2890 (CH, CH_2_); 1710 (C = O); 1614, 1566 (C = C). *^1^H-NMR (500 MHz, DMSO-d_6_, ppm)*: *δ* 12.20, 10.08 (∼50%, 50%) (s, 1H, 2′-OH); 10.80, 11.70 (∼26%, 74%) (s, 1H, CONH); 9.30, 9.50 (∼40%, 60%) (s, 1H, 3′-OH); 8.39 (d, 1H, *J* = 7.5 Hz, H_4’_); 8.37 (s, 1H, H_2_); 8.38 (s, 1H, N = CH); 8.17 (d, *J* = 8.0 Hz, 1H, H_5_); 7.87 (m, 1H, H_7_); 7.73 (m, 1H, H_8_); 7.26-7.31 (m, 1H, H_4′_); 7.00 (m, 1H, H_6′_); 6.72 (m, 1H, H_5′_); 5.20, 4.82 (∼22%, 78%) (s, 2H, NCH_2_CO). 13*C NMR (125 MHz, DMSO-d_6_, ppm): δ δ* 167.9 (CONH); 161.1 (C_2′_); 160.7 (C = O); 158.6 (C_4′_); 149.1 (N = CH); 148.6 (C_8_=C–N = C_2_); 143.0 (C_2_); 134.9 (C_7_); 131.5 (C_6_); 127.7 (C_5_); 127.6 (C_6_); 126.5 (C_8_); 121.9 (C_5_=C–C = O); 112.0 (C_1′_); 108.4 (C_5′_); 102.9 (C_3′_); 47.3 (NCH_2_CO). MS (ESI) *m/z* 338.9 [M + H]^+^.

#### (E)-N'-(2,4-Dihydroxybenzylidene)-2-(4-oxoquinazolin-3(4H)-yl)acetohydrazide (5l)

White solid; Yield: 42%. mp: 175.1–176.8 °C. *R_f_*=0.64 (DCM: MeOH = 14: 1). *IR (KBr, cm^−1^):* 3450 (NH); 3182 (OH); 3057 (CH, aren); 2978 (CH, CH_2_); 1732 (C = O); 1631, 1539 (C = C). *^1^H-NMR (500 MHz, DMSO-d_6_, ppm)*: *δ* 11.89, 11.54 (∼26%, 74%) (s, 1H, CONH); 11.09 (s, 1H, 2′-OH); 9.83 (s, 1H, 4′-OH); 8.39 (d, 1H, *J* = 7.5 Hz, H_4’_); 8.37 (s, 1H, H_2_); 8.24 (s, 1H, N = CH); 8.16 (d, *J* = 8.0 Hz, 1H, H_5_); 7.86 (t, 1H, *J* = 8.0 Hz, H_7_); 7.73 (t, 1H, *J* = 8.0 Hz, H_8_); 7.55–7.59 (m, 3H, H_6_, H_3’_, H_5’_); 6.33 (m, 1H, H_5’_); 5.16, 4.77 (∼22%, 78%) (s, 2H, NCH_2_CO). 13*C-NMR (125 MHz, DMSO-d_6_, ppm): δ* 168.3 (CONH), 163.3 (C_2’_), 161.0 (C_4’_), 160.7 (C = O), 148.9 (C_8_=C–N = C_2_), 148.5 (C_2_), 142.9 (N=CH), 134.9 (C_7_), 131.6 (C_6’_), 127.7 (C_6_), 127.5 (C_8_), 126.5 (C_5_), 121.9 (C_5_=C–C = O), 112.0 (C_1′_), 108.3 (C_5′_), 103.0 (C_3′_), 47.3 (NCH_2_CO). MS (ESI) *m/z* 338.9 [M + H]^+^. Anal. Calcd. For C_17_H_14_N_4_O_4_ (338.1015): C, 60.35; H, 4.17; N, 16.56. Found: C, 60.32; H, 4.14; N, 16.58.

#### (E)-N'-(2,5-Dihydroxybenzylidene)-2-(4-oxoquinazolin-3(4H)-yl)acetohydrazide (5m)

White solid; Yield: 38%. mp: 173.2–174.6 *R_f_*=0.56 (DCM: MeOH = 14: 1). *^1^H-NMR (500 MHz, DMSO-d_6_, ppm)*: *δ* 11.92, 11.78 (∼26%, 74%) (s, 1H, CONH); 11.78 (s, 1H, 2′-OH); 9.34 (s, 1H, 5′-OH); 8.38 (s, 1H, H_2_); 8.31, 8.39 (∼22%, 78%) (s, 1H, N = CH); 8.16 (d, *J* = 7.5 Hz, 1H, H_5_); 7.88 (t, 1H, *J* = 7.5 Hz, H_7_); 7.73 (t, 1H, *J* = 8.0 Hz, H_8_); 7.59 (t, 1H, *J* = 7.5 Hz, H_6_); 7.18 (s, 1H, H_6’_); 6.71–6.76 (m, 2H, H_3’_, H_4’_); 6.33 (m, 1H, H_5’_); 5.20, 4.79 (∼22%, 78%) (s, 2H, NCH_2_CO). 13*C-NMR (125 MHz, DMSO-d_6_, ppm): δ* 168.3 (CONH), 160.7 (C = O), 150.5 (C_2’_), 150.3 (C_2’_), 148.9 (C_8_=C-N = C_2_), 148.5 (C_2_), 142.0 (N=CH), 135.0 (C_7_), 127.6 (C_6_), 127.5 (C_8_), 127.4 (C_5_), 119.6 (C_1′_), 119.4 (C_4′_), 117.5 (C_6′_), 120.7 (C_3′_), 121.9 (C_5_=C-C = O), 47.2 (NCH_2_CO).

#### (E)-N'-(2-Hydroxy-4-methoxybenzylidene)-2-(4-oxoquinazolin-3(4H)-yl)acetohydrazide (5n)

White solid; Yield: 30%. mp: 177.1–178.3 °C. *R_f_*=0.63 (DCM: MeOH = 9: 1). *IR (KBr, cm^−1^):* 3466 (NH); 3226 (OH); 3057 (CH, aren); 2964 (CH, CH_2_); 1745 (C = O); 1612, 1562 (C = C). *^1^H-NMR (500 MHz, DMSO-d_6_, ppm)*: *δ* 11.90, 11.62 (∼26%, 74%) (s, 1H, CONH); 10.27 (s, 1H, 2’-OH); 8.38 (s, 1H, N = CH); 8.37 (s, 1H, H_2_); 8.16 (d, *J* = 8.0 Hz, 1H, H_5_); 7.89 (t, 1H, *J* = 8.0 Hz, H_7_); 7.73 (t, 1H, *J* = 8.0 Hz, H_8_);7.55 (t, 1H, *J* = 8.0 Hz, H_6_); 7.45 (d, 1H, *J* = 9.0 Hz, H_6’_); 6.47–6.52 (m, 2H, H_3’_, H_5’_); 5.20, 4.79 (∼22%, 78%) (s, 2H, NCH_2_CO); 3.77 (s, 3H, 4’-OCH_3_). 13*C-NMR (125 MHz, DMSO-d_6_, ppm):δ* 168.0 (CONH); 162.5 (C_4′_); 160.7 (C = O); 158.5 (C_2′_); 149.1 (N = CH); 148.6 (C_8_=C-N = C_2_); 142.5 (C_2_); 135.0 (C_7_); 131.4 (C_6′_); 127.7 (C_5_); 127.6 (C_6_); 126.5 (C_8_); 121.9 (C_5_=C–C = O); 113.5 (C_1′_); 107.0 (C_5′_); 101.4 (C_3′_); 55.7 (4′-OCH_3_); 47.3 (NCH_2_CO). MS (ESI) *m/z* 353.0 [M + H]^+^. Anal. Calcd. For C_18_H_16_N_4_O_4_ (352.1172): C, 61.36; H, 4.58; N, 15.90. Found: C, 61.32; H, 4.61; N, 15.93.

#### (E)-N'-(3-Hydroxy-4-methoxybenzylidene)-2-(4-oxoquinazolin-3(4H)-yl)acetohydrazide (5o)

White solid; Yield: 31%. mp: 175.2–176.5 °C. *R_f_*=0.58 (DCM: MeOH = 14: 1). *^1^H-NMR (500 MHz, DMSO-d_6_, ppm)*: *δ* 11.91, 11.62 (∼26%, 74%) (s, 1H, CONH); 9.26 (s, 1H, 3’-OH);8.36 (s, 1H, H_2_); 8.15 (d, *J* = 7.5 Hz, 1H, H_5_); 8.06, 7.91 (∼25%, 75%) (s, 1H, N = CH); 7.85 (t, 1H, *J* = 7.5 Hz, H_7_); 7.71 (t, 1H, *J* = 7.5 Hz, H_8_);7.56 (t, 1H, *J* = 7.5 Hz, H_6_); 7.22 (s, 1H, H_2’_); 7.05 (d, 1H, *J* = 8.0 Hz, H_6’_); 6.90 (d, 1H, *J* = 8.0 Hz, H_5’_); 5.19, 4.79 (∼22%, 78%) (s, 2H, NCH_2_CO); 3.80 (s, 3H, 4’-OCH_3_); 13*C-NMR (125 MHz, DMSO-d_6_, ppm): δ* 168.5 (CONH), 160.2 (C = O), 149.7 (C_4′_), 148.7 (C_3′_), 148.6 (C_8_=C–N = C_2_), 148.1 (C_2_), 144.4 (N=CH), 134.5 (C_7_), 127.2 (C_6_), 127.0 (C_8_), 126.0 (C_1′_), 121.7 (C_6′_), 121.4 (C_5_=C–C = O), 112.2 (C_2′_), 111.9 (C_6′_), 126.1 (C_5_), 55.5 (4’-OCH_3_), 47.8 (NCH_2_CO).

#### (E)-N'-(2,3,4-Trimethoxybenzylidene)-2-(4-oxoquinazolin-3(4H)-yl)acetohydrazide (5p)

White solid; Yield: 42%. mp: 183.5–184.6 °C. *R_f_*=0.60 (DCM: MeOH = 14: 1). *^1^H-NMR (500 MHz, DMSO-d_6_, ppm)*: *δ* 11.77, 11.48 (s, 1H, CONH); 8.40, 8.25 (∼25%, 75%) (s, 1H, N = CH); 8.36 (s, 1H, H_2_); 8.16 (d, *J* = 8.0 Hz, 1H, H_5_); 7.86 (t, *J* = 7.5 Hz, 1H, H_7_); 7.72 (d, *J* = 8.0 Hz, 1H, H_8_); 7.61, 7.54 (d, *J* = 8.5 Hz, 1H, H_6′_), 7.56 (t, *J* = 8.0 Hz, 1H, H_6_); 6.93, 6.91 (d, *J* = 8.5 Hz, 1H, H_2′_); 5.20, 4.76 (∼77%, 23%) (s, 2H, NCH_2_CO); 3.85 (s, 3H, OCH_3_); 3.85, 3.84 (s, 3H, OCH_3_); 3.79, 3.78 (s, 3H, OCH_3_). 13*C NMR (125 MHz, DMSO-d_6_, ppm): δ* 167.8 (CONH), 160.2 (C = O), 155.1 (C_4__′_-OCH_3_), 152.5 (C_2__′_-OCH_3_), 148.6 (C_8_=C–N = C_2_), 148.1 (C_2_), 141.6 (N=CH), 140.2 (C_3__′_-OCH_3_), 134.5 (C_7_), 127.2 (C_6_), 127.1 (C_8_), 126.0 (C_5_), 121.4 (C_5_=C–C = O), 120.5 (C_6′_), 120.0 (C_1′_), 108.7 (C_5′_), 61.7 (C_2′_-OCH_3_), 60.5 (C_3′_-OCH_3_), 56.0 (C_4′_-OCH_3_), 46.9 (NCH_2_CO).

#### (E)-N'-(3,4,5-Trimethoxybenzylidene)-2-(4-oxoquinazolin-3(4H)-yl)acetohydrazide (5q)

White solid; Yield: 48%. mp: 182.3–183.7 °C. *R_f_*=0.61 (DCM: MeOH = 14: 1). *^1^H-NMR (500 MHz, DMSO-d_6_, ppm)*: *δ* 11.79 (s, 1H, CONH); 8.65, 8.38 (s, 1H, H_2_); 8.16 (dd, *J* = 6.8, 1.0 Hz, 1H, H_5_); 8.36, 7.97 (s, 1H, N = CH); 7.86 (td, *J* = 7.5, 2.0 Hz, 1H, H_7_); 7.72 (d, *J* = 8.0 Hz, 1H, H_8_), 7.57 (td, *J* = 8.0 Hz, 2.0 Hz, 1H, H_6_); 7.22, 7.05 (∼24%, 76%) (s, 1H, H_2′_); 7.05, 7.02 (∼77%, 23%) (s, H, H_6′_); 5.24, 4.90 (∼79%, 21%) (s, 2H, NCH_2_CO); 3.84, 3.81 (∼88%, 12%) (s, 6H, C_3′_OCH_3_, C_5′_OCH_3_); 3.73, 3.71 (s, 3H, C_4′_OCH_3_). 13*C NMR (125 MHz, DMSO-d_6_, ppm): δ* 168.2 (CONH), 160.2 (C = O), 153.2 (C_3__′_-OCH_3_), 153.1 (C_5__′_-OCH_3_), 148.6 (C_8_=C–N = C_2_), 148.1 (C_2_), 144.1 (N=CH), 139.2 (C_4′_), 134.5 (C_7_), 129.4 (C_1′_), 127.2 (C_6_), 127.1 (C_8_), 126.0 (C_5_), 121.4 (C_5_=C–C = O), 104.4 (C_6′_), 104.2 (C_2′_), 60.2 (C_5′_-OCH_3_), 60.1 (C_3′_-OCH_3_), 55.9 (C_4′_-OCH_3_), 47.1 (NCH_2_CO).

#### (E)-N'-Benzylidene-2-(6-chloro-4-oxoquinazolin-3(4H)-yl)acetohydrazide (5r)

White solid; Yield: 32%. mp: 184.0–185.0 °C. *R_f_*=0.55 (DCM: MeOH = 14: 1). *IR (KBr, cm^−1^):* 3506 (NH); 3163 (OH); 3059 (CH, aren); 2980 (CH, CH_2_); 1710 (C = O); 1608, 1558 (C = C). *^1^H-NMR (500 MHz, DMSO-d_6_, ppm)*: *δ* 11.85 (s, 1H, CONH); 8.40 (s, 1H, H_2_); 8.01 (s, 1H, N = CH); 7.77 (s, 1H, H_5_); 7.92 (d, 1H, *J* = 7.0 Hz, H_7_); 7.72–7.74 (m, 3H, H_8_, H_2’_, H_6’_); 7.45–7.48 (m, 2H, H_4’_, H_5’_); 5.25, 4.79 (∼22%, 78%) (s, 2H, NCH_2_CO). 13*C-NMR (125 MHz, DMSO-d_6_, ppm): δ* 168.5 (CONH), 159.7 (C = O), 149.5 (C_8_=C–N = C_2_), 147.3 (C_2_), 144.8 (N=CH), 135.1 (C_6_), 134.3 (C_1′_), 131.9 (C_7_), 130.7 (C_4’_), 130.0 (C_5_), 129.3 (C_2’_, C_6’_), 127.3 (C_3’_, C_5’_), 125.4 (C_8_), 123.1 (C_5_=C–C = O), 47.6 (NCH_2_CO). MS (ESI) *m/z* 340.9 [M + H]^+^.

#### (E)-2-(6-Chloro-4-oxoquinazolin-3(4H)-yl)-N'-(4-fluorobenzylidene)acetohydrazide (5s)

White solid; Yield: 38%. mp: 185.0-186.0 °C. *R_f_*=0.67 (DCM: MeOH = 14: 1). *^1^H-NMR (500 MHz, DMSO-d_6_, ppm)*: *δ* 11.89, 11.83 (∼26%, 74%) (s, 1H, CONH); 8.40 (s, 1H, H_2_); 8.05 (s, 1H, H_5_); 8.01 (s, 1H, N = CH); 7.89 (d, 1H, *J* = 7.0 Hz, H_7_); 7.80 (d, 2H, *J* = 8.0 Hz, H_2’_, H_6’_); 7.57 (d, 1H, *J* = 7.0 Hz, H_8_); 7.28 (d, 2H, *J* = 8.0 Hz, H_3’_, H_5’_); 5.25 (s, 2H, NCH_2_CO). 13*C-NMR (125 MHz, DMSO-d_6_, ppm): δ* 167.9 (CONH), 164.0 (C_4′_), 159.3 (C = O), 149.0 (C_8_=C–N = C_2_), 146.8 (N=CH), 143.2 (C_2_), 134.6 (C_6_), 131.4 (C_7_), 130.4 (C_1′_), 129.5 (C_5_), 129.1 (C_2′_), 129.0 (C_6′_), 124.9 (C_8_), 122.6 (C_5_=C–C = O), 116.0 (C_3’_), 115.9 (C_5′_), 47.1 (NCH_2_CO). MS (ESI) *m/z* 357.1 [M-H]^-^.

#### (E)-2-(6-Chloro-4-oxoquinazolin-3(4H)-yl)-N'-(4-methoxybenzylidene)acetohydrazide (5t)

White solid; Yield: 40%. mp: 187.0–188.1 °C. *R_f_*=0.60 (DCM: MeOH = 14: 1). *IR (KBr, cm^−1^):* 3211 (N = C = H aromatic); 3159 (N = C-H hydrazon); 3061, 2993 (CH, aren); 2904, 2829 (CH, CH_2_); 1710, 1672, 1660, 1610, 1519 (C = C, C = N, C = O); 1242 (C-OCH_3_). *^1^H-NMR (500 MHz, DMSO-d_6_, ppm)*: *δ* 11.73, 11.70 (∼26%, 74%) (s, 1H, CONH); 8.40 (s, 1H, H_2_); 8.16 (s, 1H, N = CH); 8.09 (s, 1H, H_5_); 7.88 (d, 1H, *J* = 8.5 Hz, H_7_); 7.75 (d, 1H, *J* = 8.5 Hz, H_8_); 7.66 (d, 2H, *J* = 9.0 Hz, H_2’_, H_6’_); 7.01 (d, 1H, *J* = 9.0 Hz, H_3’_); 5.21, 4.77 (∼77%, 23%) (s, 2H, NCH_2_CO); 3.84 (s, 3H, 4’-OCH_3_); 13*C-NMR (125 MHz, DMSO-d_6_, ppm): δ* 168.5 (CONH), 160.8 (C = O), 159.2 (C_4’_), 149.0 (C_8_=C–N = C_2_), 146.2 (N=CH), 144.2 (C_2_), 134.6 (C_6_), 131.4 (C_7_), 129.5 (C_5_), 128.4 (C_2′_, C_6′_), 126.4 (C_1′_), 124.9 (C_8_), 122.6 (C_5_=C–C = O), 114.3 (C_3′_, C_5′_), 55.3 (4’-OCH_3_), 47.1 ((NCH_2_CO). MS (ESI) *m/z* 370.9 [M + H]^+^. Anal. Calcd. For C_18_H_15_ClN_4_O_3_ (370.0833): C, 58.31; H, 4.08; N, 15.11. Found: C, 58.34; H, 4.11; N, 15.08.

#### (E)-N'-Benzylidene-2-(6-methyl-4-oxoquinazolin-3(4H)-yl)acetohydrazide (5u)

White solid; Yield: 34%. mp: 180.0–181.0 °C. *R_f_*=0.68 (DCM: MeOH = 9: 1). *IR (KBr, cm^−1^):* 3450 (NH); 3223 (OH); 3080 (CH, aren); 2956 (CH, CH_2_); 1732 (C = O); 1608, 1570 (C = C). *^1^H-NMR (500 MHz, DMSO-d_6_, ppm)*: *δ* 11.88, 11.80 (∼26%, 74%) (s, 1H, CONH); 8.31 (s, 1H, H_2_); 8.24, 8.08 (∼22%, 78%) (s, 1H, N = CH); 7.96 (s, 1H, H_5_); 7.69 (d, 1H, *J* = 8.5 Hz, H_7_); 7.75 (d, 2H, *J* = 8.0 Hz, H_2’_, H_6’_); 7.62 (d, 1H, *J* = 8.5 Hz, H_8_); 7.43-7.49 (m, 3H, H_3’_, H_4’_, H_5’_); 5.23, 4.79 (∼78%, 22%) (s, 2H, NCH_2_CO); 2.47 (s, 3H, 6-CH_3_). 13*C-NMR (125 MHz, DMSO-d_6_, ppm): δ* 168.3 (CONH), 160.2 (C = O), 147.8 (C_8_=C–N = C_2_), 146.1 (C_2_), 144.3 (N=CH), 138.9 (C_6_), 135.7 (C_7_), 133.9 (C_1′_), 130.1 (C_4′_), 128.9 (C_2′_, C_6′_), 127.1 (C_5_), 126.9 (C_3′_, C_5′_), 125.3 (C_8_), 121.2 (C_5_=C–C = O), 47.0 (NCH_2_CO), 20.8 (6-CH_3_). MS (ESI) *m/z* 321.0 [M + H]^+^. Anal. Calcd. For C_16_H_18_N_4_O_2_ (320.1273): C, 67.49; H, 5.03; N, 17.49. Found: C, 58.34; H, 4.11; N, 15.08.

#### (E)-2-(6-Methyl-4-oxoquinazolin-3(4H)-yl)-N'-(2-nitrobenzylidene)acetohydrazide (5v)

White solid; Yield: 37%. mp: 182.2–183.4 °C. *R_f_*=0.72 (DCM: MeOH = 9: 1). *^1^H-NMR (500 MHz, DMSO-d_6_, ppm)*: *δ* 12.12, 12.06 (∼26%, 74%) (s, 1H, CONH); 8.63, 8.44 (∼23%, 77%) (s, 1H, N = CH); 8.30 (s, 1H, H_2_); 7.94 (s, 1H, H_5_); 8.12 (d, 1H, *J* = 8.0 Hz, H_6’_); 8.08 (d, 1H, *J* = 8.0 Hz, H_3’_); 7.80 (m, 1H, H_5’_); 7.69 (d, 1H, *J* = 8.0 Hz, H_7_); 7.67 (m, 1H, H_4’_); 7.62 (d, 1H, *J* = 8.0 Hz, H_8_); 5.20, 4.79 (∼77%, 23%) (s, 2H, NCH_2_CO); 2.50 (s, 3H, 6-CH_3_). 13*C-NMR (125 MHz, DMSO-d_6_, ppm): δ* 168.6 (CONH), 160.1 (C = O), 148.1 (C_8_=C–N = C_2_), 147.6 (C_2’_), 146.0 (C_2_), 143.0 (N=CH), 136.6 (C_6_), 135.7 (C_7_), 133.6 (C_5′_), 130.7 (C_4′_), 128.3 (C_6′_), 128.0 (C_1′_), 127.1 (C_5_), 125.3 (C_8_), 124.6 (C_3’_), 121.2 (C_5_=C–C = O), 46.8 (NCH_2_CO), 20.8 (6-CH_3_).

#### (E)-N'-(4-Fluorobenzylidene)-2-(6-methyl-4-oxoquinazolin-3(4H)-yl)acetohydrazide (5w)

White solid; Yield: 42%. mp: 183.2–184.5 °C. *R_f_*=0.66 (DCM: MeOH = 14: 1). *^1^H-NMR (500 MHz, DMSO-d_6_, ppm)*: *δ* 11.87, 11.80 (∼25%, 75%) (s, 1H, CONH); 8.31 (s, 1H, H_2_); 8.24, 8.07 (∼22%, 78%) (s, 1H, N = CH); 7.95 (s, 1H, H_5_); 7.77–7.82 (m, 2H, H_2’_, H_6’_); 7.68 (d, 1H, *J* = 8.0 Hz, H_7_); 7.62 (d, 1H, *J* = 8.0 Hz, H_8_); 7.29 (t, *J* = 8.0 Hz, 2H, H_3’_, H_5’_); 5.22, 4.78 (∼78%, 22%) (s, 2H, NCH_2_CO); 2.46 (s, 3H, 6-CH_3_). 13*C-NMR (125 MHz, DMSO-d_6_, ppm): δ* 168.2 (CONH), 164.1 (C_4′_), 160.2 (C = O), 147.7 (C_8_=C–N = C_2_), 146.1 (C_2_), 143.1 (N=CH), 136.8 (C_6_), 135.7 (C_7_), 130.5 (C_1′_), 129.1 (C_2′_, C_6′_), 127.1 (C_5_), 125.3 (C_8_), 121.2 (C_5_=C–C = O), 116.0 (C_3′_, C_5′_), 46.9 (NCH_2_CO), 20.8 (6-CH_3_). MS (ESI) *m/z* 337.1 [M-H]^-^.

#### (E)-N'-(4-Bromobenzylidene)-2-(6-methyl-4-oxoquinazolin-3(4H)-yl)acetohydrazide (5y)

White solid; Yield: 28%. mp: 182.6–183.5 °C. *R_f_*=0.70 (DCM: MeOH = 9: 1). *^1^H-NMR (500 MHz, DMSO-d_6_, ppm)*: *δ* 11.92, 11.85 (∼22%, 78%) (s, 1H, CONH); 8.29 (s, 1H, H_2_); 8.20, 8.03 (∼23%, 77%) (s, 1H, N = CH); 7.94 (s, 1H, H_5_); 7.69 (d, 1H, *J* = 8.5 Hz, H_7_); 7.65 (d, *J* = 8.5 Hz, 4H, H_3’_, H_5’,_ H_2’_, H_6’_); 7.60 (d, 1H, *J* = 8.5 Hz, H_8_); 5.20, 4.76 (∼77%, 23%) (s, 2H, NCH_2_CO); 2.45(s, 3H, 6-CH_3_). 13*C-NMR (125 MHz, DMSO-d_6_, ppm): δ* 168.3 (CONH), 160.1 (C = O), 147.7 (C_8_=C–N = C_2_), 146.1 (C_2_), 143.0 (N=CH), 136.8 (C_6_), 135.7 (C_7_), 133.2 (C_1′_), 131.8 (C_3′_, C_5′_), 128.8 (C_2′_, C_6′_), 127.1 (C_5_), 125.3 (C_8_), 123.3 (C_4′_), 121.2 (C_5_=C–C = O), 46.9 (NCH_2_CO), 20.8 (6-CH_3_).

#### (E)-N'-(4-Methoxybenzylidene)-2-(6-methyl-4-oxoquinazolin-3(4H)-yl)acetohydrazide (5x)

White solid; Yield: 40%. mp: 186.5–187.5 °C. *R_f_*=0.70 (DCM: MeOH = 9: 1). *IR (KBr, cm^−1^):* 3460 (NH); 3188 (OH); 3093 (CH, aren); 2839 (CH, CH_2_); 1772 (C = O); 1608, 1519 (C = C). *^1^H-NMR (500 MHz, DMSO-d_6_, ppm)*: *δ* 11.74, 11.67 (∼22%, 78%) (s, 1H, CONH); 8.31 (s, 1H, H_2_); 8.18, 8.01 (∼22%, 78%) (s, 1H, N = CH); 7.96 (s, 1H, H_5_); 7.01 (d, *J* = 8.5 Hz, 2H, H_3’_, H_5’_); 7.69 (d, 1H, *J* = 8.5 Hz, H_7_); 7.67 (d, *J* = 8.5 Hz, 2H, H_2’_, H_6’_);7.62 (d, 1H, *J* = 8.5 Hz, H_8_); 5.20, 4.77 (∼77%, 23%) (s, 2H, NCH_2_CO); 3.81 (s, 3H, 4’-OCH_3_); 2.51 (s, 3H, 6-CH_3_). 13*C-NMR (125 MHz, DMSO-d_6_, ppm): δ* 168.0 (CONH), 160.8 (C_4′_), 160.2 (C = O), 147.8 (C_8_=C-N = C_2_), 146.1 (C_2_), 144.1 (N=CH), 136.8 (C_6_), 135.7 (C_7_), 128.5 (C_2′_, C_6′_), 127.1 (C_5_), 126.5 (C_1′_), 125.3 (C_8_), 121.2 (C_5_=C-C = O), 114.3 (C_3′_, C_5′_), 55.3 (4’-OCH_3_), 46.9 (NCH_2_CO), 20.8 (6-CH_3_). MS (ESI) *m/z* 351.0 [M + H]^+^. Anal. Calcd. For C_19_H_18_N_4_O_2_ (350.1379): C, 65.13; H, 5.18; N, 15.99. Found: C, 65.15; H, 5.15; N, 15.62.

#### (E)-N'-(4-(dimethylamino)benzylidene)-2-(6-methyl-4-oxoquinazolin-3(4H)-yl)acetohydrazide (5z)

Light brown solid; Yield: 26%. mp: 186.0-187.0 °C. *R_f_*=0.68 (DCM: MeOH = 9: 1). *IR (KBr, cm^−1^):* 3439 (NH); 3348 (OH); 3122 (CH, aren); 2791 (CH, CH_2_); 1735 (C = O); 1610, 1537 (C = C). *^1^H-NMR (500 MHz, DMSO-d_6_, ppm)*: *δ* 11.54, 11.49 (∼27%, 73%) (s, 1H, CONH); 8.29 (s, 1H, H_2_); 8.07, 7.91 (∼23%, 77%) (s, 1H, N = CH); 7.94 (s, 1H, H_5_); 7.67 (d, 1H, *J* = 8.0 Hz, H_7_); 7.61 (d, 1H, *J* = 8.0 Hz, H_8_); 7.53 (d, 2H, *J* = 8.5 Hz, H_2’_,H_6’_); 6.74 (d, 2H, *J* = 8.5 Hz, H_3’_,H_5’_); 5.16, 4.72 (∼77%, 23%) (s, 2H, NCH_2_CO);3.97 (s, 6H, 4’-N(CH_3_)_2_); 2.42 (s, 3H, 6-CH_3_). 13*C-NMR (125 MHz, DMSO-d_6_, ppm): δ* 167.6 (CONH), 160.2 (C = O), 151.4 (C_4′_), 148.8 (C_8_=C–N = C_2_), 146.1 (C_2_), 145.0 (N=CH), 136.8 (C_6_), 135.7 (C_7_), 128.5 (C_1′_), 128.2 (C_2′_, C_6′_), 127.0 (C_5_), 125.3 (C_8_), 121.3 (C_5_=C–C = O), 111.8 (C_3′_, C_5′_), 46.8 (NCH_2_CO). 20.8 (6-CH_3_, 4’-N(CH_3_)_2_). MS (ESI) *m/z* 364.0 [M + H]^+^. Anal. Calcd. For C_20_H_21_N_5_O_2_ (363.1695): C, 66.10; H, 5.82; N, 19.27. Found: C, 66.14; H, 5.80; N, 19.30.

#### (E)-N'-(furan-2-ylmethylene)-2-(4-oxoquinazolin-3(4H)-yl)acetohydrazide (6a)

Light brown solid; Yield: 44%. mp: 181.0–182.3 °C. *R_f_*=0.68 (DCM: MeOH = 9: 1). *^1^H-NMR (500 MHz, DMSO-d_6_, ppm)*: *δ* 11.77 (s, 1H, CONH); 8.37, 8.36 (s, 1H, H_2_); 8.17 (dd, *J* = 8.0 Hz, 1.0 Hz, 1H, H_5_); 8.14, 7.96 (s, 1H, N = CH), 8.0 (dd, *J* = 13.5 Hz, 1.5 Hz, 1H, H_5′_); 7.87 (td, *J* = 8.5 Hz, 1.5 Hz, 1H, H_7_); 7.74 (d, *J* = 8.0 Hz, 1H, H_8_), 7.58 (td, *J* = 8.0 Hz, 1.0 Hz, 1H, H_6_); 6.96, 6.96 (d, *J* = 3.5 Hz, 1H, H_3′_); 6.65 (dd, *J* = 3.5 Hz, 1.5 Hz, 1H, H_4′_); 5.16, 4.79 (∼77%, 23%) (s, 2H, NCH_2_CO). 13*C NMR (125 MHz, DMSO-d_6_, ppm): δ* 168.1 (CONH), 160.3 (C = O), 148.9 (C_1′_), 148.6 (C_8_=C–N = C_2_), 148.1 (C_2_), 145.2 (C_5′_), 134.5 (C_7_), 134.4, 127.2 (C_6_), 127.1 (C_8_), 126.0 (C_5_), 121.4 (C_5_=C–C = O), 113.9 (C_3′_), 112.2 (C_4′_), 46.9 (NCH_2_CO). Anal. Calcd. For C_15_H_12_N_4_O_3_ (296.0909): C, 60.81; H, 4.08; N, 18.91. Found: C, 60.85; H, 4.11; N, 18.94.

#### (E)-2-(4-Oxoquinazolin-3(4H)-yl)-N'-(thiophen-2-ylmethylene)acetohydrazide (6b)

White solid; Yield: 26%. mp: 182.1–183.6 °C. *R_f_*=0.72 (DCM: MeOH = 9: 1). *^1^H-NMR (500 MHz, DMSO-d_6_, ppm)*: *δ* 11.84, 11.81 (s, 1H, CONH); 8.45, 8.26 (∼25%, 75%) (s, 1H, N = CH); 8.37 (s, 1H, H_2_); 8.17 (dd, *J* = 8.0 Hz, 1.5 Hz, 1H, H_5_); 7.89 (td, *J* = 8.0 Hz, 1.5 Hz, 1H, H_7_); 7.74 (d, *J* = 8.0 Hz, 1H, H_8_); 7.69 (d, *J* = 5.5 Hz, 1H, H_5′_); 7.60 (td, *J* = 8.0 Hz, 1.0 Hz, 1H, H_6_); 7.50 (dd, *J* = 8.5 Hz, 1.0 Hz, 1H, H_3′_); 7.17 (dd, *J* = 5.0 Hz, 3.5 Hz, 1H, H_4′_); 5.15, 4.78 (∼75%, 25%) (s, 2H, NCH_2_CO). 13*C NMR (125 MHz, DMSO-d_6_, ppm): δ* 167.9 (CONH); 160.3 (C = O); 148.6 (C_8_=C–N = C_2_); 148.1 (C_2_); 139.5 (N = CH); 138.5 (C_2′_); 134.5 (C_7_); 130.9 (C_3′_); 128.8 (C_5′_); 128.0 (C_4′_); 127.2 (C_6_); 127.1 (C_8_); 126.0 (C_5_); 121.5 (C_5_=C–C = O); 46.8 (NCH_2_CO). Anal. Calcd. For C_15_H_12_N_4_O_2_S (312.0681): C, 57.68; H, 3.87; N, 17.94. Found: C, 57.64; H, 3.91; N, 17.97.

#### (E)-N'-((1-Methyl-1H-pyrrol-2-yl)methylene)-2–(4-oxoquinazolin-3(4H)-yl)acetohydrazide (6c)

White solid; Yield: 58%. mp: 184.5–185.2 °C. *R_f_*=0.70 (DCM: MeOH = 9: 1). *^1^H-NMR (500 MHz, DMSO-d_6_, ppm)*: *δ* 11.54, 11.48 (∼18%, 82%) (s, 1H, CONH); 8.36 (s, 1H, H_2_); 8.17 (dd, *J* = 8.0 Hz, 1.0 Hz, 1H, H_5_); 7.97 (s, 1H, N = CH); 7.88 (td, *J* = 8.0 Hz, 1.5 Hz, 1H, H_7_); 7.73 (d, *J* = 8.0 Hz, 1H, H_8_); 7.58 (td, *J* = 8.0 Hz, 1.0 Hz, 1H, H_6_); 7.00 (s, 1H, H_5′_); 6.51 (dd, *J* = 3.5 Hz, 1.5 Hz, 1H, H_3′_), 6.11 (dt, *J* = 3.5 Hz, 2.5 Hz, 1H, H_4′_); 5.15, 4.76 (∼88%, 12%) (s, 2H, NCH_2_CO); 3.89, 3.82 (∼82%, 18%) (s, 3H, CH_3_). 13*C NMR (125 MHz, DMSO-d_6_, ppm): δ* 168.0 (CONH); 160.8 (C = O); 149.1 (C_8_=C–N = C_2_); 148.6 (C_2_); 138.1 (N = CH); 134.9 (C_7_); 128.8 (C_2′_); 127.7 (C_5′_); 127.6 (C_6_); 127.2 (C_8_); 126.5 (C_5_); 121.9 (C_5_=C–C = O); 115.8 (C_3′_); 108.6 (C_4′_); 47.4 (NCH_2_CO); 37.0 (CH_3_). Anal. Calcd. For C_16_H_15_N_5_O_2_ (309.1226): C, 62.13; H, 4.89; N, 22.64. Found: C, 62.09; H, 4.92; N, 22.67.

#### (E)-2–(4-Oxoquinazolin-3(4H)-yl)-N'-(pyridin-2-ylmethylene)acetohydrazide (6d)

White solid; Yield: 36%. mp: 183.0–184.0 °C. *R_f_*=0.34 (DCM: MeOH = 9: 1). *^1^H-NMR (500 MHz, DMSO-d_6_, ppm)*: *δ* 12.11, 12.03 (∼20%, 80%) (s, 1H, CONH); 8.64 (d, *J* = 8.0 Hz, 1H, H_6′_); 8.40 (s, 1H, H_2_); 8.18 (d, *J* = 8.0 Hz, 1H, H_5_); 8.26, 8.12 (∼20%, 80%) (s, 1H, N = CH); 8.03 (d, *J* = 8.0 Hz, 1H, H_3′_); 7.93-7.86 (m, 2H, H_7_, H_4′_); 7.74 (d, *J* = 8.0 Hz, 1H, H_8_); 7.60 (td, *J* = 7.5 Hz, 2.0 Hz, 1H, H_6_); 7.46 (td, *J* = 5.0 Hz, 1.0 Hz, 1H, H_5′_), 5.28, 4.83 (∼81%, 19%) (s, 2H, NCH_2_CO). 13*C NMR (125 MHz, DMSO-d_6_, ppm): δ* 169.0 (CONH); 160.8 (C = O); 153.2 (C_2′_); 150.1 (C_6′_); 149.0 (C_8_=C–N = C_2_); 148.6 (C_2_); 145.2 (N = CH); 137.4 (C_4′_); 135.1 (C_7_); 127.7 (C_6_); 127.6 (C_8_); 126.5 (C_5_); 125.0 (C_5′_); 121.9 (C_5_=C–C = O); 120.3 (C_3′_); 47.4 (NCH_2_CO). Anal. Calcd. For C_16_H_13_N_5_O_2_ (307.1069): C, 62.53; H, 4.26; N, 22.79. Found: C, 62.57; H, 4.23; N, 22.82.

#### (E)-2–(4-Oxoquinazolin-3(4H)-yl)-N'-(pyridin-3-ylmethylene)acetohydrazide (6e)

White solid; Yield: 39%. mp: 182.5–183.6 °C. *R_f_*=0.36 (DCM: MeOH = 9: 1). *^1^H-NMR (500 MHz, DMSO-d_6_, ppm)*: *δ* 12.00 (s, 1H, CONH); 8.91, 8.86 (∼80%, 20%) (d, *J* = 2.0 Hz, 1H, H_2′_); 8.63, 8.61 (∼88%, 12%) (dd, *J* = 5.0 Hz, 1.5 Hz, 1H, H_6′_); 8.39, 8.31 (∼83%, 17%) (s, 1H, H_2_); 8.18 (dd, *J* = 8.0 Hz, 1.5 Hz, 2H, H_5_, H_4′_); 8.12 (s, 1H, N = CH); 7.89 (td, *J* = 8.0 Hz, 1.5 Hz, 1H, H_7_); 7.74 (d, *J* = 8.0 Hz, 1H, H_8_); 7.60 (td, *J* = 8.0 Hz, 2.0 Hz, 1H, H_6_); 7.51 (dd, *J* = 8.0 Hz, 4.5 Hz, 1H, H_5′_), 5.27, 4.82 (∼79%, 21%) (s, 2H, NCH_2_CO). 13*C NMR (125 MHz, DMSO-d_6_, ppm): δ* 168.9 (CONH); 160.7 (C = O); 151.1 (C_4′_); 149.3 (C_2′_, C_8_=C–N = C_2_); 148.6 (C_2_); 142.0 (N = CH); 135.0 (C_6’_); 133.9 C_7_); 130.3 (C_1’_); 127.7(C_6_); 127.6 (C_8_); 126.5 (C_5_); 124.4 (C_5′_); 121.9 (C_5_=C–C = O); 47.5 (NCH_2_CO). Anal. Calcd. For C_16_H_13_N_5_O_2_ (307.1069): C, 62.53; H, 4.26; N, 22.79. Found: C, 62.50; H, 4.27; N, 22.74.

#### (E)-2–(4-Oxoquinazolin-3(4H)-yl)-N'-(pyridin-4-ylmethylene)acetohydrazide (6f)

White solid; Yield: 27%. mp: 181.2–182.5 °C. *R_f_*=0.40 (DCM: MeOH = 9: 1). *^1^H-NMR (500 MHz, DMSO-d_6_, ppm)*: *δ* 12.14, 12.08 (∼25%, 75%) (s, 1H, CONH); 8.64 (d, 2H, *J* = 5.0 Hz, H_2’_, H_6’_); 8.30 (s, 1H, H_2_); 8.15 (d, 1H, *J* = 8.0 Hz, H_5_); 8.05 (s, 1H, N = CH); 7.87 (t, 1H, *J* = 8.0 Hz, H_7_); 7.72 (d, 1H, *J* = 8.0 Hz, H_8_); 7.69 (d, 2H, *J* = 5.0 Hz, H_2’_, H_6’_); 7.57 (t, 1H, *J* = 8.0 Hz, H_6_); 5.26, 4.81 (∼75%, 25%) (s, 2H, NCH_2_CO). 13*CN-MR (125 MHz, DMSO-d_6_, ppm): δ* 168.6 (CONH), 160.2 (C = O), 150.2 (C_3′_, C_5′_), 148.5 (C_8_=C–N = C_2_), 148.4 (C_2_), 144.4 (N=CH), 141.9 (C_1’_), 134.5 (C_7_), 127.2 (C_6_), 127.1 (C_8_), 126.0 (C_5_), 121.4 (C_5_=C–C = O), 120.8 (C_2′_, C_6′_), 46.9 (NCH_2_CO). Anal. Calcd. For C_16_H_13_N_5_O_2_ (307.1069): C, 62.53; H, 4.26; N, 22.79. Found: C, 62.51; H, 4.28; N, 22.76.

#### (E)-N'-((5-Methylfuran-2-yl)methylene)-2–(4-oxoquinazolin-3(4H)-yl)acetohydrazide (6g)

White solid; Yield: 39%. mp: 180.9–181.7 °C. *R_f_*=0.70 (DCM: MeOH = 9: 1). *^1^H-NMR (500 MHz, DMSO-d_6_, ppm)*: *δ* 11.72, 11.66 (∼25%, 75%) (s, 1H, CONH); 8.35 (s, 1H, H_2_); 8.15 (d, 1H, *J* = 8.0 Hz, H_5_); 8.01, 7.86 (∼22%, 78%) (s, 1H, N = CH); 7.86 (t, 1H, *J* = 8.0 Hz, H_7_); 7.72 (d, 1H, *J* = 8.0 Hz, H_8_); 7.54 (t, 1H, *J* = 8.0 Hz, H_6_); 6.83 (d, 1H, *J* = 3.5 Hz, H_4’_); 6.27 (d, 1H, *J* = 3.5 Hz, H_3’_); 5.14, 4.76 (∼75%, 25%) (s, 2H, NCH_2_CO); 2.36 (s, 3H, 2’-CH_3_). 13*C-NMR (125 MHz, DMSO-d_6_, ppm): δ* 167.9 (CONH), 160.2 (C = O), 154.7 (C_3′_), 148.6 (C_8_=C–N = C_2_), 148.1 (C_2_), 147.5 (C_1′_), 136.9 (N=CH), 134.5 (C_7_), 127.2 (C_6_), 127.1 (C_8_), 126.0 (C_5_), 121.4 (C_5_=C–C = O), 115.7 (C_5′_), 108.6 (C_4′_), 46.8 (NCH_2_CO), 13.5 (3′-CH_3_). Anal. Calcd. For C_16_H_14_N_4_O_3_ (310.1066): C, 61.93; H, 4.55; N, 18.06. Found: C, 61.97; H, 4.59; N, 18.09.

#### (Z)-N'-(2-Oxoindolin-3-ylidene)-2–(4-oxoquinazolin-3(4H)-yl)acetohydrazide (7a)

Light yellow solid; Yield: 44%. mp: 183.3–184.0 °C. *R_f_*=0.72 (DCM: MeOH = 9: 1). *^1^H-NMR (500 MHz, DMSO-d_6_, ppm)*: *δ* 12.71 (s, 1H, NH-isatin); 11.30 (s, 1H, CONH); 8.40 (s, 1H, H_2_); 8.16 (d, 1H, *J* = 8.0 Hz, H_5_); 7.87 (t, 1H, *J* = 8.0 Hz, H_7_); 7.73 (d, 1H, *J* = 8.0 Hz, H_8_); 7.59 (d, 1H, *J* = 7.5 Hz, H_7’_); 7.57 (t, 1H, *J* = 8.0 Hz, H_6_); 7.41 (t, 1H, *J* = 7.5 Hz, H_5’_); 7.12 (t, 1H, *J* = 7.0 Hz, H_6’_); 6.97 (d, 1H, *J* = 7.5 Hz, H_4’_); 5.37 (s, 2H, NCH_2_CO); 13*C-NMR (125 MHz, DMSO-d_6_, ppm): δ* 168.9 (CONH), 162.4 (C_2’_=O),160.3 (C_4_=O), 148.3 (C_8_=C–N = C_2_), 148.0 (C_2_), 142.7 (C_7’_–C–NH), 135.4 (N=CH), 134.6 (C_7_, C_2’_), 131.9 (C_4’_), 127.3 (C_6_, C_6’_), 126.0 (C_5_, C_8_), 122.6 (C_5’_), 121.3 (C_5_=C–C = O), 120.8 (C_3’_–C–C_4’_), 111.2 (C_7’_), 46.5 (NCH_2_CO). Anal. Calcd. For C_18_H_13_IN_5_O_3_ (347.1018): C, 62.24; H, 3.77; N, 20.16. Found: C, 62.29; H, 3.81; N, 20.19.

#### (Z)-2-(6-Iodo-4-oxoquinazolin-3(4H)-yl)-N'-(2-oxoindolin-3-ylidene)acetohydrazide (7b)

Light yellow solid; Yield: 57%. mp: 183.7–184.5 °C. *R_f_*=0.73 (DCM: MeOH = 9: 1). *IR (KBr, cm^−1^):* 3450 (NH); 3184 (OH); 3049 (CH, aren); 2902 (CH_2_); 1724 (C = O); 1685, 1523 (C = C). *^1^H-NMR (500 MHz, DMSO-d_6_, ppm)*: *δ* 11.71 (s, 1H, NH-isatin); 10.87 (s, 1H, CONH); 8.43 (s, 2H, H_2_, H_5_); 8.17 (d, 2H, *J* = 8.25 Hz, H_7_, H_8_); 7.53 (d, 1H, *J* = 8.5 Hz, H_7’_); 7.40 (d, 1H, *J* = 6.85 Hz, H_5’_); 7.04 (br, 1H, H_6’_); 6.92 (d, 1H, *J* = 7.95 Hz, H_4’_); 5.30 (s, 2H, NCH_2_CO); 13*C-NMR (125 MHz, DMSO-d_6_, ppm): δ* 170.8 (CONH), 164.3 (C_2’_=O),158.9 (C_4_=O), 149.1 (C_8_=C–N = C_2_), 147.3 (C_2_), 144.0 (C_7_), 143.0 (C_7’_–C–NH); 134.3 (N=CH, C_5_), 132.9 (C_6’_), 129.5 (C_4’_, C_8_), 126.2 (C_5_), 123.1 (C_5’_), 121.8 (C_5_=C–C = O), 115.1 (C_3’_–C–C_4’_), 110.7 (C_7’_), 92.3 (C_6_), 47.5 (NCH_2_CO). Anal. Calcd. For C_16_H_12_IN_5_O_3_ (363.1695): C, 66.10; H, 5.82; N, 19.27. Found: C, 66.14; H, 5.80; N, 19.30.

### 2.2. Cytotoxicity assay

The cytotoxicity of the synthesised compounds was evaluated against three human cancer cell lines, including SW620 (colon cancer), PC3 (prostate cancer), and NCI-H23 (lung cancer). The cell lines were purchased from a Cancer Cell Bank at the Korea Research Institute of Bioscience and Biotechnology (KRIBB). The media, sera and other reagents that were used for cell culture in this assay were obtained from GIBCO Co. Ltd. (Grand Island, New York, USA). The cells were culture in DMEM (Dulbecco’s Modified Eagle Medium) until confluence. The cells were then trypsinized and suspended at 3 × 10^4^ cells/mL of cell culture medium. On day 0, each well of the 96-well plates was seeded with 180 μL of cell suspension. The plates were then incubated in a 5% CO_2_ incubator at 37 °C for 24 h. Compounds were initially dissolved in dimethyl sulfoxide (DMSO) and diluted to appropriate concentrations by culture medium. Then 20 μL of each compounds’ samples, which were prepared as described above, were added to each well of the 96-well plates, which had been seeded with cell suspension and incubated for 24-h, at various concentrations. The plates were further incubated for 48 h. Cytotoxicity of the compounds was measured by the colorimetric method, as described previously^18^ with slight modifications^19–22^. The IC_50_ values were calculated using a Probits method^23^ and were averages of three independent determinations (the standard deviation: SD ≤ 10%).

### 2.3. Caspase-3 activation assay

U937 cells (5 × 10^5^ cells/well) were plated in 6-well plate, and allowed to be stabilised overnight. The cells were treated with PAC-1 or compounds (50 μM in 0.1% dimethyl sulfoxide). After 24 h incubation, cells were harvested and washed twice with PBS. The cells were lysed by 50 μL of chilled cell lysis buffer for 10 min on 4 °C. Cell lysate (200 μg/100 μL/well) were mixed with Ac-DEVD-pNA (200 μM). ODs at 405 nm were measured every 30 min for 12 h. The slope of the linear portion for each well was determined as the enzyme activity.

### 2.4. Docking studies

The docking simulations were carried out using the Molecular Operating Environment version MOE 2009.10^24^. The 3 D structure of PAC-1 was directly obtained from Pubchem (https://pubchem.ncbi.nlm.nih.gov/compound/pac-1) and the chemical structures of the other compounds were constructed using the Builder module in MOE. All compounds were solvated with H_2_O as solvent layer width to 10 Å. The potential energy was minimised up to 0.0001 gradients using the MMFF94x force field cut-off. For protein preparation, the crystal structures of pseudo-activated procaspase-3 (3ITN)^25^ and zinc-bound caspase-6 (4FXO)^26^ enzymes were retrieved from the Protein Data Bank (www.rcsb.org) and prepared by employing the QuickPrep module. The binding site was determined on the basis of the PLB (Propensity for Ligand Binding) score in the Site Finder module of MOE^24^. All the water molecules in the active site were kept for docking. Docking assays were performed by MOE Dock, using the default flexible ligand/rigid receptor protocol, Triangle Matcher placement, as previously reported^27^. We used London dG as first rescoring function with force field (MMFF94x) refinement, and GBVI/WSA as a second one to estimate the negative binding free energy profile of the complex (S value, kCal/mol). The best pose with the highest negative S value was selected for each ligand. Biovia DS Visualiser can be utilised as a visualisation tool to show up the potential interactions of the ligands to the residues in the binding sites of caspase enzymes^28^.

## 3. Results and discussions

### 3.1. Chemistry

The target acetohydrazides incorporating quinazolin-4(3*H*)-one **(5a**–**z**, **6a–g**, **7a**–**b)** were synthesised via four step pathway, as illustrated in scheme 1. The first step was a Niementowski condensation of anthranilic acid (**1a**) or 5-substituted-2-aminobenzoic acid (**1b–d**) and formamide at 120 °C to obtain quinazoline-4(3*H*)-one derivatives (**2a–d**) in quantitative yield (93–97%). In the second step, an acylation between quinazoline-4(3*H*)-one derivatives (**2a–d**) and ethyl chloroacetate under basic conditions (K_2_CO_3_) in acetone with a catalytic amount of KI gave the selectively *N_3_-*alkylated intermediate esters **3a–d** ^29^. These esters **3a–d** participated in futher acyl nucleophile substitution with hydrazine monohydrate at the third step. This reaction proceeded smoothly in ethanol under refluxing condition. The final step in the pathway involved an aldol condensation of hydrazids **4a–d** with benzaldehydes or isatins. The desired products **5a–z**, **6a–g**, **7a**–**b** were obtained in moderate overall yields.

The structures of the synthesised compounds were determined straightforwardly based on analysis of spectroscopic data, including IR, MS, ^1^H and ^13^C NMR. Full NMR and MS spectra can be found in Supporting Information. Quinazoline-4(3*H*)-ones (**2a–d**) could react with ethyl chloroacetate to form both *N*_3_- and *O*-alkylated products, depending on the reaction conditions. However, it has been demonstrated that, when acetone was used as the reaction solvent, the alkylation reaction gave only *N*_3_-alkylated products^29^. The formation of *N*_3_-alkylated products was evidenced by NMR spectroscopic data. Generally, in the ^1^H NMR spectra of the final products (**5a–z**, **6a–g**, **7a**–**b**), the singlet peaks attributable for two methylene protons appeared at around 4.8–5.4 ppm, corresponding to the methylene protons of *N*_3_-alkylated compounds^29^. For the *O*-alkylated products, these methylene protons normally appear more downfield (5.6–5.7 ppm) in the ^1^H NMR spectra. In the ^13^C NMR spectra, one peak appeared at around 167–169 ppm was attributable for C = O of the CONHN = group. Other peak appeared at around 160–161 ppm was attributable for C_4_=O of the *N*_3_-alkylated products. For the *O*-alkylated products, the carbon of C_4_-O functionality should appear more downfield at around 167–168 ppm in the ^13^C NMR spectra^29^. It means that, if the products were *O*-alkylated, there should be two peaks at around 167–169 ppm in the ^13^C NMR spectra. Additionally, in the in the ^13^C NMR spectra of all final products, there was a peak around 47 ppm, which was also typical of the methylene carbon from the -NCH_2_CO- moiety. In case of *O*-alkylated products, a peak for the carbon of this moiety should appear at around 77 ppm^22^.

**Figure 2.**
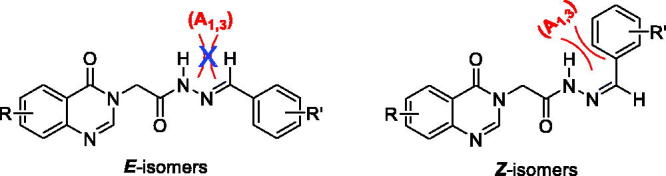
Structure of *E/Z* isomers of acetohydrazides.

**Figure 3.**
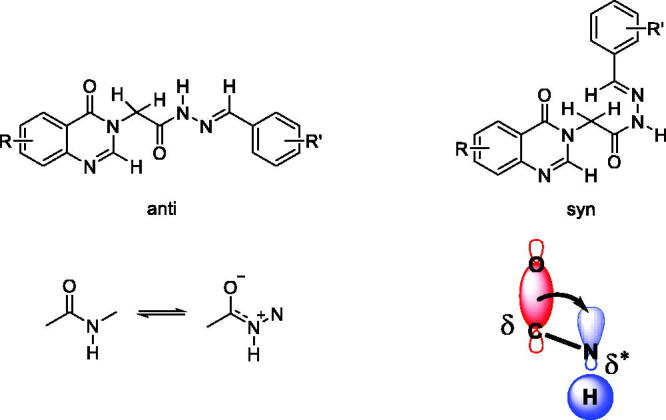
Structure of *anti-E/syn-E* isomers of acetohydrazides.

Regarding the representation of the amide and imine functional groups, the acetohydrazides (**5a–z**, **6a–g**, **7a**–**b**) could form 4 isomers: *anti-Z*, *anti-E*, *syn-Z*, and *syn-E*. In the cases of aromatic aldehydes-condensed *N*-acylhydrazones, ^1^H-NMR spectra in DMSO-*d_6_* showed 2 peaks of the -CH_2_CO group at around 5.24 ppm and 4.49 ppm with a ratio of 3:1. It could be attributed to the presence of two possible *syn-anti* isomers of the amide bond or *Z-E* isomers of the imine bond. To confirm the structures, we decided to proceed with NOESY experiment. The NOESY spectrum indicated that each diagonal signal from a conformer showed a cross-correlation with the corresponding signal of the other conformer. Thus, the cross-correlation was caused by multi-conformers with a rotation bond (*syn-* and *anti-*isomers), not by a non-rotatable imine bond (*Z* and *E* isomers). Moreover, because of A (1,3) strain in *Z*-isomer (Figure 2), the *E-*isomer is more stable than *Z*-isomer. Therefore, the hydrogen of NH amide and C = O can be *syn-* or *anti-* position in their relationship in an *E* imine configuration (Figure 3).

As it has been known, an amide bond is a partial double bond with a partial oxygen anion. Because the oxygen anion has *sp2* hybridisation, the partial anion should be located in the *sp2* orbital with high *s* character for stabilisation. This orbital makes a sigma (δ) bond with a carbon atom and will be stabled by *anti*-bond sigma* (δ*) at *anti-* position. When a hydrogen atom is at *anti-* position, partial anion will donate electron to the hydrogen atom causes increasing density electron on the hydrogen and the proton is shifted upfield (Figure 3). Combining with ^1^H-NMR spectra, it was clearly shown that the major peak of NH in the upfield was *anti-*isomer. This observation was in agreement with the study of Palla and coworkers^30^, where upfield chemical shift of NH was *anti-*isomer. Thus, it could be concluded that the acetohydrazides (**5a–z**, **6a–g**, **7a**–**b**) formed *anti-E* and *syn-E* isomers with a ratio of about 3:1, respectively.

### 3.2. Bioactivity

**Figure 4.**
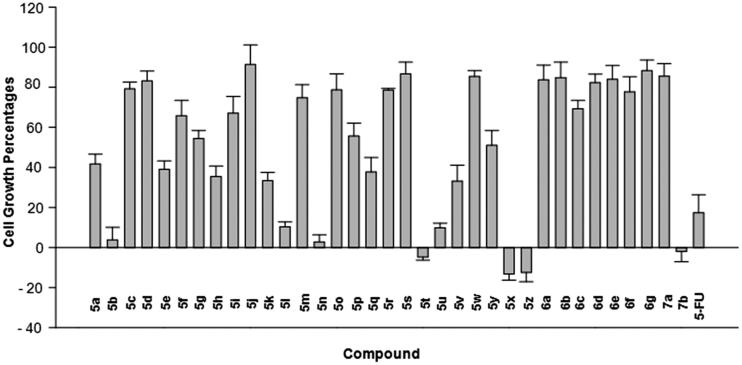
Effects of the compounds at 30 µg/mL on the SW620 cells growth.

The synthesised compounds were subjected to the cytotoxicity assay using SRB method as described previously^18^ with slight modifications^19–22^. In the first screening, we evaluated the effects of the compounds on the growth of SW620 (colon cancer) cell line. The cell growth percentages (CGP) of the cells in the presence of respective compounds at 30 µg/mL were measured and presented in Table 1 and Figure 4. The results were averages of three separate measurements. 5-Fluorouracil was used as a positive control.

**Table 1.** Effects of the compounds on the growth of SW620 human colon cancer cells. 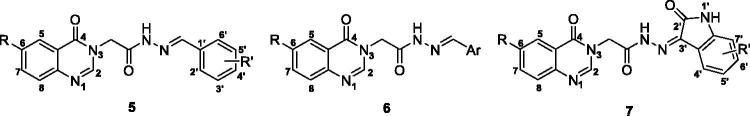

Cpd	R	R’/Ar	CGP (%)^a^	Cpd	R	R’/Ar	CGP (%)^a^
**5a**	-H	H	41.70 ± 4.89	**5r**	-Cl	-H	78.58 ± 0.81
**5b**	-H	2-Cl	**3.88 ± 6.23**	**5s**	-Cl	4-F	86.71 ± 5.86
**5c**	-H	2-NO_2_	79.18 ± 3.46	**5t**	-Cl	4-OCH_3_	**−4.75 ± 1.51**
**5d**	-H	3-Cl	83.23 ± 4.86	**5u**	-CH_3_	-H	**9.91 ± 2.26**
**5e**	-H	4-Cl	39.09 ± 4.17	**5v**	-CH_3_	2-NO_2_	33.21 ± 7.89
**5f**	-H	4-F	65.77 ± 7.72	**5w**	-CH_3_	4-F	85.41 ± 2.89
**5g**	-H	4-Br	54.46 ± 3.97	**5y**	-CH_3_	4-Br	51.05 ± 7.38
**5h**	-H	2-OH	35.50 ± 5.15	**5x**	-CH_3_	4-OCH_3_	**−13.24 ± 3.12**
**5i**	-H	4-OH	67.09 ± 8.29	**5z**	-CH_3_	4-(NCH_3_)_2_	**−12.43 ± 4.66**
**5j**	-H	4-OCH_3_	91.40 ± 9.78	**6a**	-H		83.73 ± 7.37
**5k**	-H	2,3-(OH)_2_	33.40 ± 4.15	**6b**	-H		84.82 ± 7.72
**5l**	-H	2,4-(OH)_2_	**10.38 ± 2.44**	**6c**	-H		69.25 ± 4.17
**5m**	-H	2,5-(OH)_2_	74.70 ± 6.61	**6d**	-H		82.31 ± 4.31
**5n**	-H	2-OH-4-OCH_3_	**2.81 ± 3.61**	**6e**	-H		84.00 ± 6.92
**5o**	-H	3-OH-4-OCH_3_	78.68 ± 8.05	**6f**	-H		77.79 ± 7.49
**5p**	-H	2,3,4-(OCH_3_)_3_	55.67 ± 6.37	**6g**	-H		88.32 ± 5.28
**5q**	-H	3,4,5-(OCH_3_)_3_	37.76 ± 7.16	**7a**	-H	-H	85.58 ± 6.17
**5-FU**^b^			17.35 ± 9.01	**7b**	-I	-H	**−1.99 ± 5.14**

^a^Cell growth percentage, compounds were assayed at 30 µg/mL; ^b^5-FU: 5-Fluorouracil, a positive control.

Because this is our first investigation into the compounds of acetohydrazide type incorporating quinazolin-4(3*H*)-4-one moiety as potential anticancer agents, we initially designed 26 compounds (**5a–5z**) to get a glance on general structural features required for antitumor cytotoxicity. Seventeen compounds (**5a–5q**), bearing different substituents (R’) on the phenyl part and no substituent on the 4-oxoquinazolin-4(3*H*) skeleton, were designed to investigate the effects of the substituents on the phenyl moiety. Three compounds **5r–5t** and five compounds **5u–5z** were designed to preliminarily investigate the effects of the electron-withdrawing and electron-releasing substituents on the quinazolin-4(3*H*)-4-one moiety towards the cytotoxicity of compounds in series **5**. The synthesis of compound bearing 3-allyl-3-hydroxy substituent on the phenyl ring in series **5** was not attempted at this stage of investigation because structure-activity relationships study of PAC-1 previously indicated that the 3-allyl substituent on the phenyl ring of C-region in the structure of PAC-1 was not very important for PAC-1 bioactivity^7^ ^,^ ^20^. In addition, 3-allyl-2-hydroxybenzaldehyde was not readily accessible.

It can be seen from Table 1 that compounds **5a–5q**, with few exceptions, only moderately inhibited the growth of SW620 cells. In general, substitution at position 2 seemed to be more favourable for inhibition of cell growth. For example, compound **5 b** at 30 µg/mL displayed very strong cellular inhibition towards the growth of SW620 cells (CGP = 3.88 ± 6.23%), while compounds **5d** and **5e** exhibited much weaker effects (CGP = 83.23 ± 4.86 and 39.09 ± 4.17%, respectively). Compound **5 h** with 2-OH substituent was also significantly more potent (CGP = 35.50 ± 5.15%) than compound **5i** (CGP = 67.09 ± 8.29%), which was 4-OH substituted. In case of compound **5 h**, the addition of one extra OH group at position 3 or 5 did not enhance the cytotoxicity. However, it was found that, addition of an extra OH group at position 4 significantly increased the cytotoxicity, as observed with compound **5 l** (CGP = 10.38 ± 2.44%). Especially, addition of the methoxy group at position 4 into compound **5 h** resulted in compound **5n**, which was the most potent among the compounds **5a–5q**. Multimethoxylated compounds **5p** and **5q** were not very active. From these results, three substituents, including 2-OH-4-OCH_3_, 2,4-(OH)_2_, and 2-Cl, were found to be most potential for the cytotoxicity.

Introduction of the 6-Cl substituent on the quinazolin-4(3*H*)-4-one part of compounds **5a** and **5f** did not enhance the cytotoxicity of these compounds (as seen with the high CGP values of the resulting compounds **5r** and **5 s**, Table 1). However, when the 6-Cl substituent was added on the quinazolin-4(3*H*)-4-one moiety of compound **5j**, the cytotoxicity of the resulting compound **5t** was substantially increased (as demonstrated by the CGP value of this compound (−4.75 ± 1.51%, Table 1) in comparison to that of compound **5j** (91.40 ± 9.78%, Table 1). Interestingly, compound **5x** which has the same 4-methoxy group on the phenyl ringside and 6-methyl substituent on the 4-oxoquinazolin-4(3*H*) part was also found to be strongly inhibitory against the growth of SW620 cells at the tested concentration of 30 µg/mL. Compound **5z** with the 4-dimethylamino group instead of 4-methoxy one was similarly potent. Introduction of the 6-methyl substituent on the quinazolin-4(3*H*)-4-one part of compound **5a** was also found to enhance the cytotoxicity of the resulting compound **5 u**. Thus, collectively, from the above results, it could be suggested that for compounds of general structure **5**, substituents like 2-chloro, 2-OH-4-CH_3_, 2,4-(OH)_2_ or 4-methoxy and 4-dimethylamino on the phenyl part were found to be favourable for cytotoxicity. Introduction of different substituents on the quinazolin-4(3*H*)-4-one moiety could also potentially increase the cytotoxicity of the resulting compounds. Replacement of the substituted phenyl moiety by isatin could also induce potential compounds (e.g. compound **7 b**, which completely inhibited the growth of SW620 cells when present at 30 µg/mL). However, replacement of the substituted phenyl moiety by different heterocyclic rings like furane, thiophene, pyrrole, or pyridine, all proved to be detrimental for cytotoxicity of the resulting compounds (**6a–6g**).

From the screening on SW620 cells, 8 compounds including **5 b, 5 l, 5n, 5t, 5 u, 5x, 5z** and **7 b** were found to have potential cytotoxicity (Table 1, Figure 4). These compounds were subjected to further evaluation against three cancer cell lines, including SW620, PC-3 (prostate cancer), and NCI-H23 (lung cancer), to get the IC_50_ values. The results are presented in Table 2. It was found that all these 8 compounds exhibited good cytotoxicity against three cell lines tested with IC_50_ values in low µM range. Except for compounds **5 l**, all other 7 compounds were more potent than 5-FU, which was used as a positive control. Compound **5t** was the most potent in the series with IC_50_ values of 3- to 5-fold lower than that of 5-FU. Compared to PAC-1, compounds **5 l** and **5n** were less cytotoxic in three human cancer cell lines tested. Compounds **5x**, **5z** were equally potent compared to PAC-1 while four compounds, including **5 b, 5t, 5 u**, and **7 b**, were more potent. All compounds have logP values in a favourable range for oral administration (Table 2). In term of logP values, these compounds appeared to be more ideal than PAC-1 (logP, 3.43). Our results clearly demonstrate the potentials of the quinazolin-4(3*H*)-4-one-based acetohydrazides as anticancer agents.

**Table 2.** Cytotoxicity of the selected compounds against some human cancer cell lines. 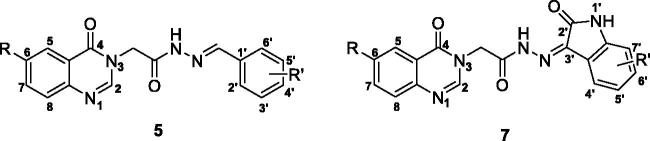

Cpd code	R	R’/Ar	MW	LogP^a^	Cytotoxicity (IC_50_,^b^ μM)/Cell lines^c^		
					SW620	PC3	NCI-H23
**5b**	-H	2-Cl	340.76	1.52	3.14 ± 0.56	3.99 ± 0.15	4.49 ± 0.24
**5l**	-H	2,4-(OH)_2_	338.32	0.63	9.43 ± 0.27	16.52 ± 0.24	18.97 ± 3.43
**5n**	-H	2-OH-4-OCH_3_	352.34	1.19	6.10 ± 0.68	8.46 ± 1.28	9.85 ± 1.28
**5t**	-Cl	4-OCH_3_	370.79	1.60	2.45 ± 0.03	3.10 ± 0.32	3.34 ± 0.32
**5u**	-CH_3_	-H	320.35	1.42	3.52 ± 0.03	5.78 ± 0.06	9.77 ± 0.31
**5x**	-CH_3_	4-OCH_3_	350.37	1.50	4.99 ± 0.37	4.22 ± 0.63	4.05 ± 0.31
**5z**	-CH_3_	4-(NCH_3_)_2_	363.41	1.60	4.68 ± 0.30	4.60 ± 0.33	3.58 ± 0.11
**7b**	-I	-H	473.22	1.21	3.97 ± 0.32	3.25 ± 0.15	2.81 ± 0.17
**5-FU**^d^			130.08	−0.81	8.84 ± 1.92	13.61 ± 0.46	13.45 ± 3.92
**PAC-1**			392.49	3.43	5.82 ± 0.20	4.16 ± 0.52	5.32 ± 0.21

^a^Calculated by EPI 320 software; ^b^The concentration (µM) of compounds that produces a 50% reduction in enzyme activity or cell growth, the numbers represent the averaged results from triplicate experiments with deviation of less than 10%.; ^c^Cell lines: SW620, colon cancer; PC3, prostate cancer; NCI-H23, lung cancer; ^d^5-FU: 5-Fluorouracil, a positive control.

**Figure 5.**
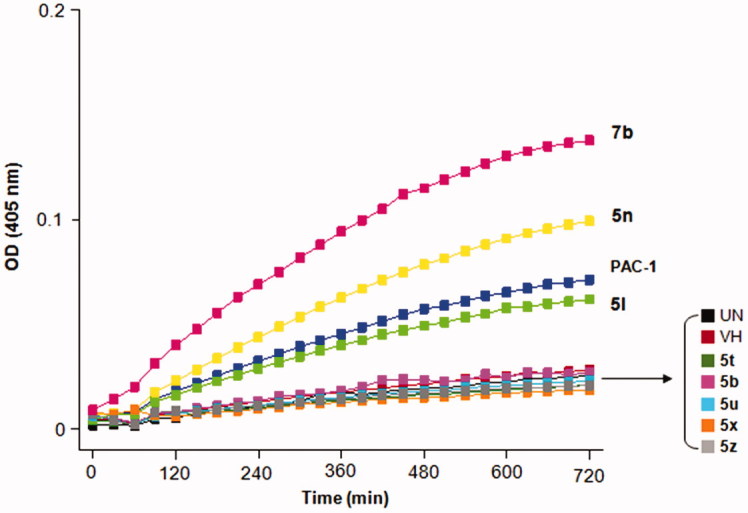
Caspases activation activity of some representative compounds. UN, untreated; VH, vehicle.

**Figure 6.**
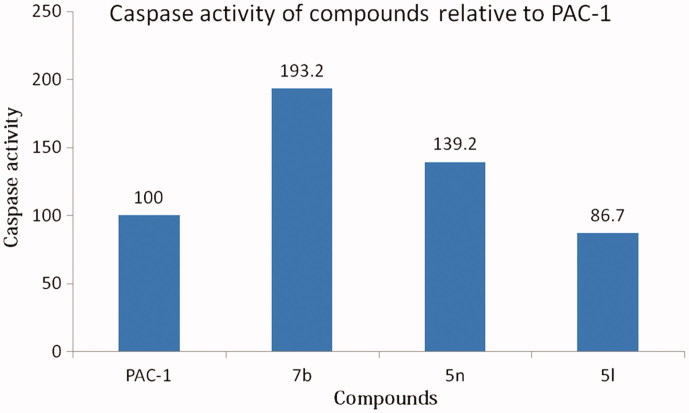
Relative caspases activation activity of some compounds in comparison to PAC-1. Compounds were tested at 50 µM.

Eight compounds including **5 b, 5 l, 5n, 5t, 5 u, 5x, 5z** and **7 b** were preliminarily evaluated for their ability to induce caspases activity using U937 cells. The compounds or PAC-1, which was used as a positive control, were incubated with U937 cells (5 × 10^5^ cells/well) for 24 h. The cells were then harvested and lysed. The cell lysates (200 μg/100 μL/well) were mixed with Ac-DEVD-pNA (a substrate of caspases 3, 6 and 7). The OD values were then measured at 405 nm every 30 min for 12 h. The slope of the linear portion for each well was determined as the enzyme activity and presented in Figure 5. Noteworthy, three compounds including **7 b**, **5n**, and **5 l** caused significant elevation in caspases activity. Especially, compound **7 b** activated caspases activity by almost 200% in comparison to that of PAC-1 (Figure 6). The caspases activation activity of compound **5n** was approximately 1.4-fold stronger than that of PAC-1. Compound **5 l** was slightly less potent than PAC-1 in term of caspases activation. From these results, it was likely that the 2-hydroxy substituents on the phenyl ring might play an important role for caspases activation of compounds with a general structure **5**.

In this study, however, it was difficult to delineate a correlation between caspases activation and cytotoxicity of the compounds. Compounds **7 b** and **5t** were found to be similarly potent in term of cytotoxicity but compound **7 b** was the most potent as a caspases activator while the caspases activation activity of compound **5t** was not very significant. Also, it was found that despite five compounds **5 b, 5t, 5 u, 5x,** and **5z** exhibited potent cytotoxicity, they showed only minimal caspases activation activity. Thus, more in-depth studies need to be carried out to fully understand the cytotoxic mechanisms of these compounds.

### 3.3. Molecular docking studies of 5 b, 5n, 5t, and 7b

As previously identified, compounds **5 b**, **5t**, and **7 b** provided two to five-fold increases in cytotoxicity against different cell-lines compared to reference compound 5-FU. Especially, two compounds **7 b** and **5n** have emerged as potential caspases activators and anticancer agents compared to PAC-1. It is of great interest to probe the binding ability of four-hit candidates **5 b**, **5n**, **5t**, and **7 b** towards procaspase-3 by using molecular docking simulation and then compare these results with those of PAC-1.

According to the catalytic mechanism, these compounds, as derived from PAC-1, would activate procaspase-3 by chelating zinc *via* the key *ortho*-hydroxy *N*-acyl hydrazone moiety, thus relieving the zinc-mediated inhibition^16^. Unfortunately, a crystal structure of entire zinc-bound procaspase-3 has not been determined so far, then it is difficult to directly study the role of zinc binding in ligand-induced procaspase activation. Therefore, we decided to perform the docking assays using another executer caspase highly homologous to caspase-3: caspase-6 locked by zinc whose crystal complex structure was recently solved by Delgado and Hardy^26^. In addition, to confirm that all the compounds can bind to the allosteric site of procaspase-3 which also play a key role in the active site formation at the dimer interface of the enzyme^31^ ^,^ ^32^, we studied the interactions between **5 b**, **5n**, **5t**, and **7 b** towards procaspase-3 by using the X-ray crystallographic structure of the V266E procaspase-3 mutant (replacement of Val with Glu at position 266) reported by Walters et al.^25^

**Figure 7.**
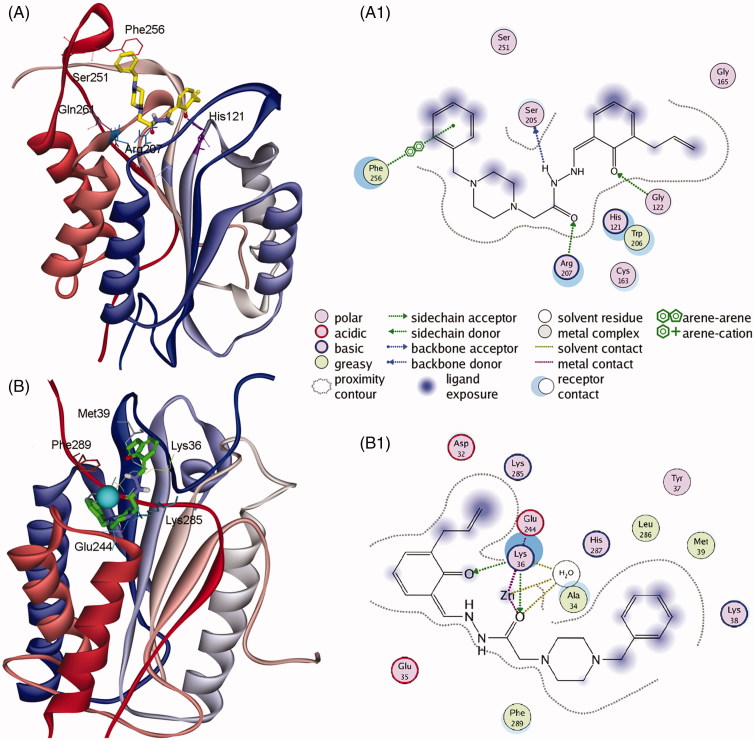
Representations of the reference activator PAC-1 docked into procaspase-3 (PDB: 3ITN) showing the **(A)** 3D conformation and **(A1)** 2D interactions in the allosteric site, and PAC-1 docked into zinc-bound caspase-6 (PDB: 4FXO) showing **(B)** 3D orientation and **(B1)** 2D interactions in the allosteric inhibitory site. The zinc ion is represented as a light blue sphere.

Firstly, PAC-1 was docked into the allosteric site of procaspase-3. The results showed that PAC-1 could strictly interact with numerous residues at this site, including Ser205, Arg207, Asn208, Ser251, and Phe256 (Figure 7(A, A1)). Analysing the H-bonding network also revealed the significant contribution of water-mediated H-bond between benzylpiperazine moiety and Arg207, Ser209, and Glu246. The binding affinity was −4.96 kCal/mol. Subsequently, for the first time, PAC-1 was docked into the zinc binding site of caspase-6. In the crystal structure, the zinc is found in an exosite and was liganded by Lys36, Glu244, and His287 with a water molecule serving as the fourth ligand, forming a distorted tetrahedral ligation sphere^26^. We sought to determine the ability of PAC-1 to chelate zinc and relieve this ion from this allosteric inhibitory site of caspase-6. As the results of docking, PAC-1 was able to chelate zinc and more importantly perturbated the tetrahedral coordination geometry of zinc to Lys36, Glu244, and His287 (Figure 7(B1)). The carbonyl oxygens of PAC-1 can also form multiple H-bond interactions with Lys36 and water molecule, suggesting the higher affinity of PAC-1 to this allosteric site compared to zinc ion.

**Figure 8.**
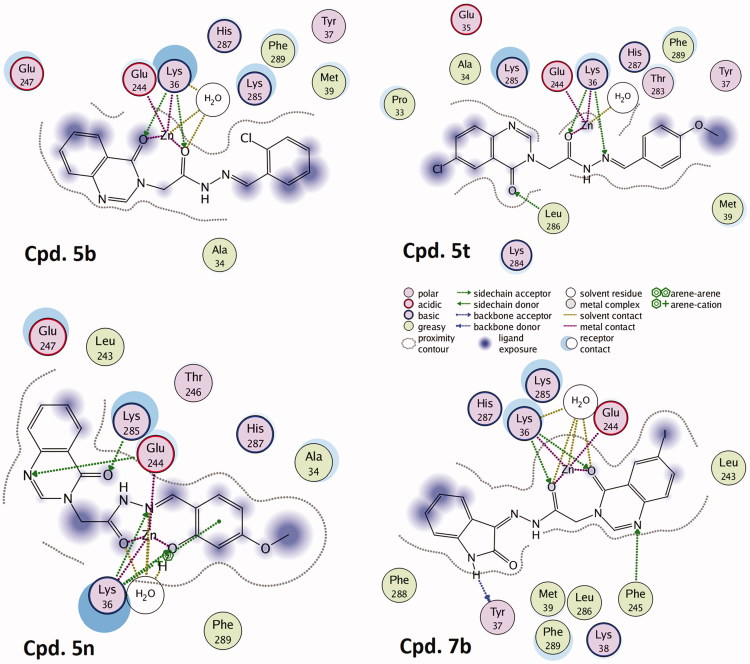
Topological interactions between **5b**, **5n**, **5t**, and **7b** with caspase-6 showing the role of the *ortho*-hydroxy *N*-acyl hydrazone moiety in chelating zinc in the allosteric site.

The next steps involved in docking selected compounds **5 b**, **5n**, **5t**, and **7 b** into the allosteric site of procaspase-3. The 3 D binding modes of these compounds were depicted in Figure 8. At first sight, all compounds **7 b** displayed the highest affinity (−5.94 kCal/mol) among four analogues (S values of **5 b**, **5n** and **5t** were −4.46, −5.09 and −4.23 kCal/mol, respectively), and this value is higher than that of PAC-1. In particular, compound **5n** and **7 b** provided extensive interactions with residues in the active site of procaspase-3, such as Arg208 and Phe250 (with 4-oxoquinazoline), and Cys163, Tyr204, and Phe256 (with arylidene moieties). We can also identify that the allosteric binding site principally consists of hydrophobic as well as H-bond acceptor residues^32^, which in nature is favourable for the interaction with acetihydrazide derivatives having substituents as complex aromatic systems (e.g. quinazoline or oxoindoline) and H-bond donor groups (e.g. -NH or -OH).

**Figure 9.**
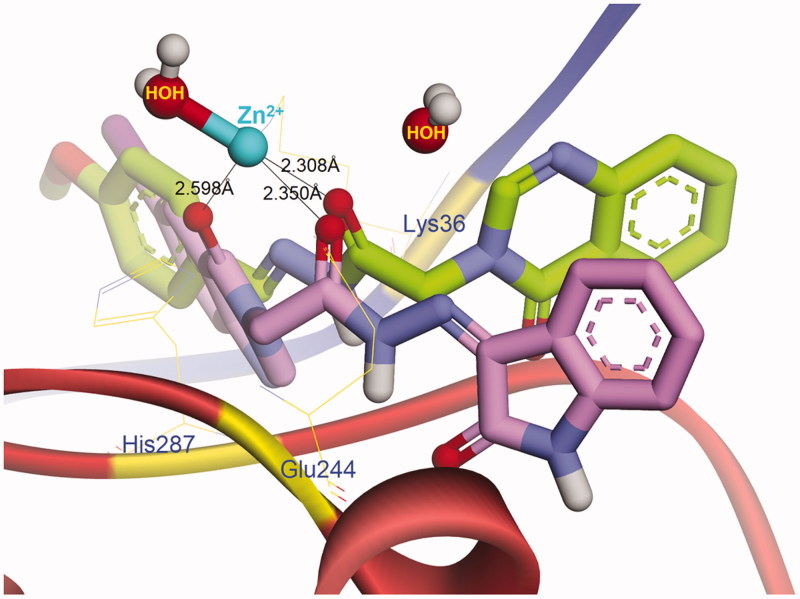
Conformations of **5n** (green carbons) and **7b** (purple carbons) docked into the allosteric inhibitory site of caspase-6.

We continued investigating the role of *ortho*-hydroxy *N*-acyl hydrazone motif in chelating zinc ion that binds to the allosteric inhibitory site of caspase-6. The topological docking representation in Figure 8 showed that all the compounds could bind to zinc as anticipated. Two carbonyl and hydroxyl oxygens of compounds **5 b** and **7 b** formed a bidentate chelation with the metal ion, meanwhile, **5t** and **5n** interacted with zinc in the same manner of PAC-1. The close distances from the carbonyl oxygens of **5n** and **7 b** to zinc ion (see Figure 9) are favourable for metal chelating. Compound **5n** and **7 b** also displayed more complex H-bonding interaction network with the central residues in the allosteric site of caspase-6, such as Lys36, Tyr37, Glu244, Phe245, Lys285 and His287, suggesting their higher binding affinity toward caspase zymogens compared to **5 b**, **5t** and PAC-1.

## 4. Conclusions

In conclusion, we have reported a series of 35 (*E*)-*N'*-arylidene-2–(4-oxoquinazolin-4(3*H*)-yl)acetohydrazides with significant cytotoxicity against three human cancer cell lines, including SW620 (human colon cancer), PC-3 (prostate cancer), and NCI-H23 (lung cancer). The most potent compound **5t** displayed cytotoxicity up to 5-fold more potent than 5-FU. Structure-activity relationship analysis revealed that when the aryl part was phenyl, substituents like 2-chloro, 2-OH-4-CH_3_, 2,4-(OH)_2_ or 4-methoxy and 4-dimethylamino were favourable for cytotoxicity of the compounds. In term of caspases activation activity, several compounds were found to exhibit potent effects, (e.g. compounds **7 b**, **5n**, and **5l**). Especially, compound **7 b** activated caspases activity by almost 200% in comparison to that of PAC-1. Subsequent docking simulation also revealed that this compound is a potent allosteric inhibitor of procaspase-3, and the central role of the *ortho*-hydroxy *N*-acyl hydrazone moiety in chelating zinc in the allosteric site. Taking all above mentioned into account, several compounds, such as **5n** and **7 b**, have emerged as potential hits for further design and development of caspases activators and anticancer agents. Further investigation of the substituents on the quinazolin-4(3*H*)-4-one part is needed to optimise the bioactivity.

## Supplementary Material

Supplemental Material
