# Supplementary material for: (E)-N'-Arylidene-2-(4-oxoquinazolin-4(3H)-yl) acetohydrazides: Synthesis and evaluation of antitumor cytotoxicity and caspase activation activity
Source: J Enzyme Inhib Med Chem. 2019 Jan 17;34(1):465–78. doi: 10.1080/14756366.2018.1555536 (PMC6338265; doi:10.1080/14756366.2018.1555536)
Supplement: Supplemental Material [file IENZ_A_1555536_SM1546.pdf]

SUPPLEMENTAL MATERIALS

for

(*E*)-*N'*-Arylidene-2-(4-oxoquinazolin-4(3*H*)-yl)acetohydrazides: Synthesis and Evaluation of Caspase Activation Activity and Cytotoxicity

Le Cong Huan,[a] Cao Viet Phuong,[a] Le Cong Truc,[a] Vo Nguyen Thanh,[a] Pham-The Hai,[a] Le-Thi-Thu Huong,[b] Nguyen Thi Thuan,[a] Eun Jae Park,[c] A Young Ji,[c] Jong Soon Kang,[d] Sang-Bae Han,[*,c] Phuong-Thao Tran,[*,a] Nguyen-Hai Nam[*,a]

[a]*Hanoi University of Pharmacy, 13-15 Le Thanh Tong, Hanoi, Vietnam*
[b]*School of Medicine and Pharmacy, Vietnam National University, 144 XuanThuy, Hanoi, VietNam*
[c]*College of Pharmacy, Chungbuk National University, 194-31, Osongsaengmyung-1, Heungdeok, Cheongju, Chungbuk, 28160, Republic of Korea*
[d]*Bio-Evaluation Center, Korea Research Institute of Bioscience and Biotechnology, Cheongju, Chungbuk, Republic of Korea*

Keywords: Acetohydrazides, quinazolin-4-one, cytotoxicity, caspase activation.

***Corresponding authors:** Tel.: +84-4-39330531; Fax: +84-4-39332332; Emails: namnh@hup.edu.vn (N.H. Nam); thaotp119@gmail.com (T.P. Thao).

ALL ^{1}H & ^{13}C NMR SPECTRA OF THE COMPOUNDS

^{1}H NMR of compound 5a

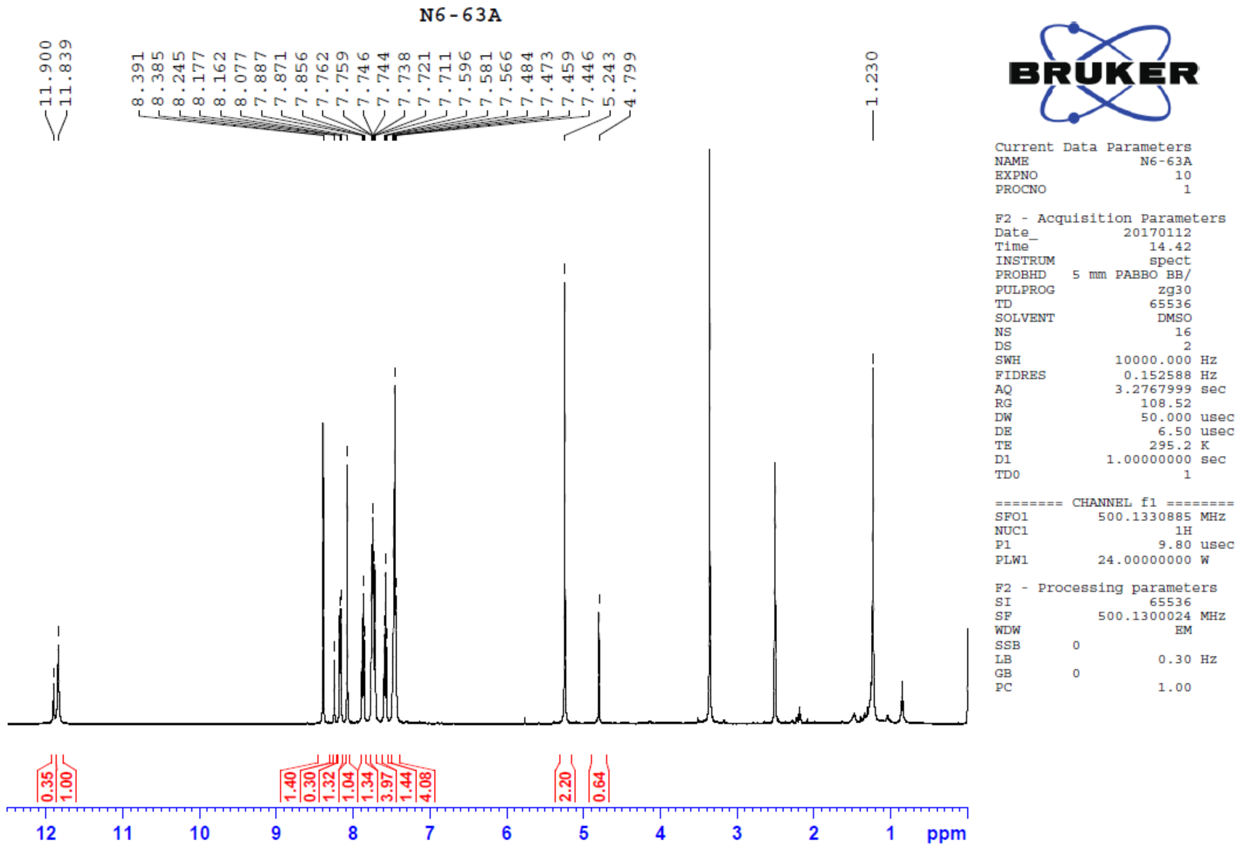

^{13}C NMR of compound 5a

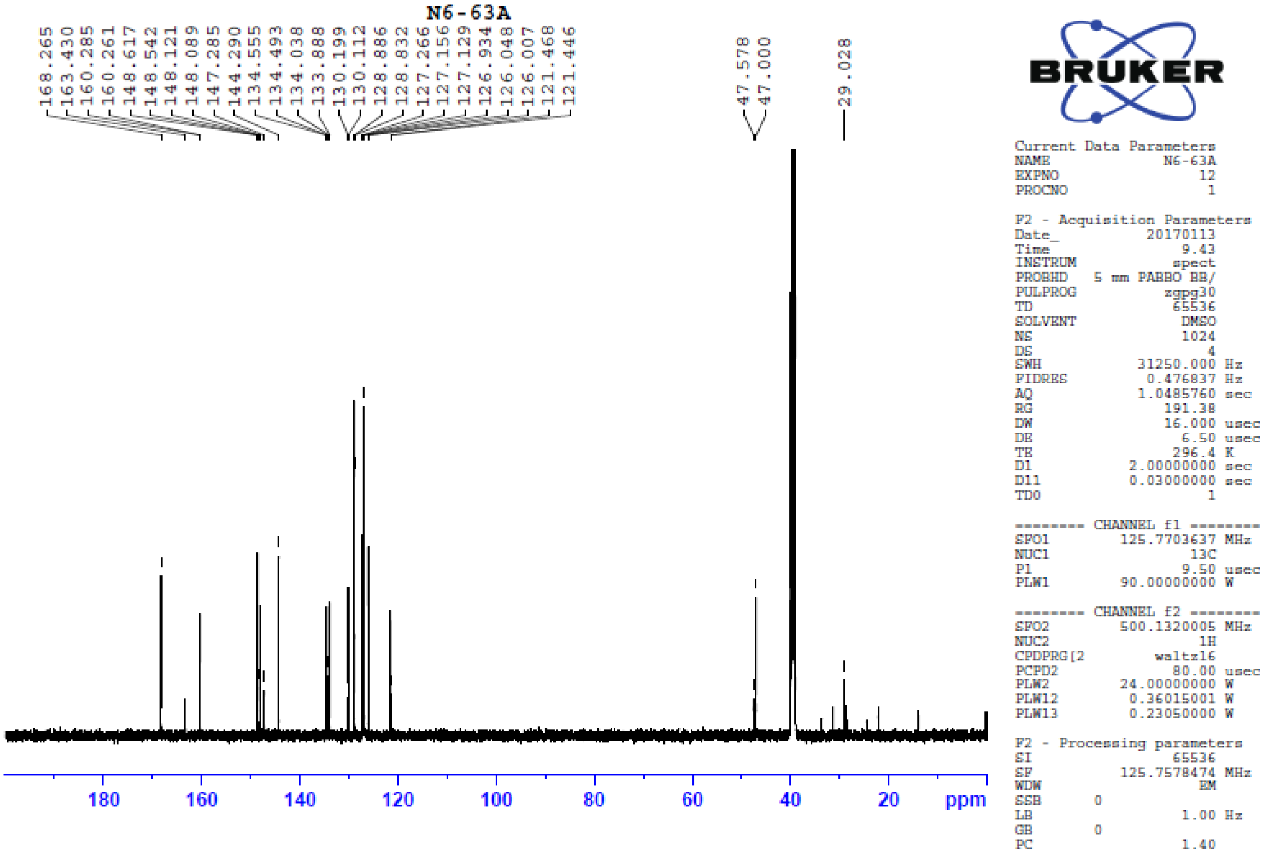

NOESY-1 of compound 5a

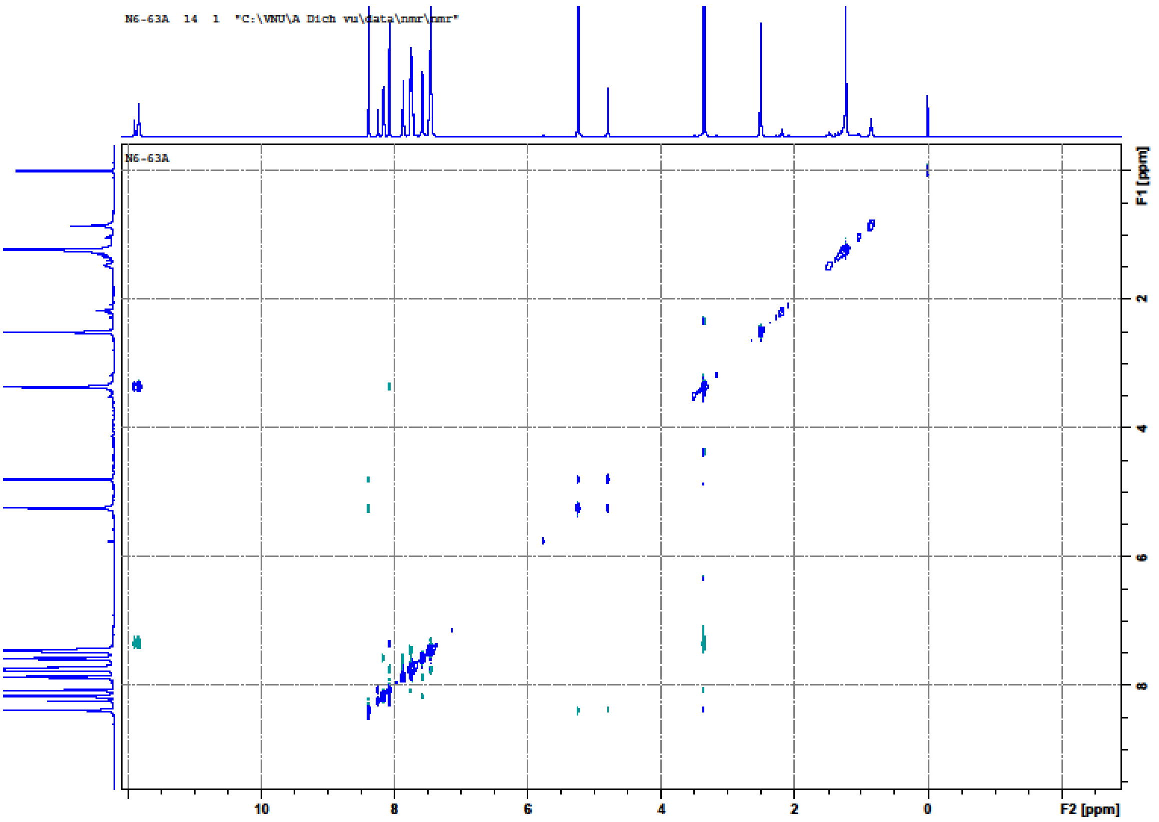

NOESY-2 of compound 5a

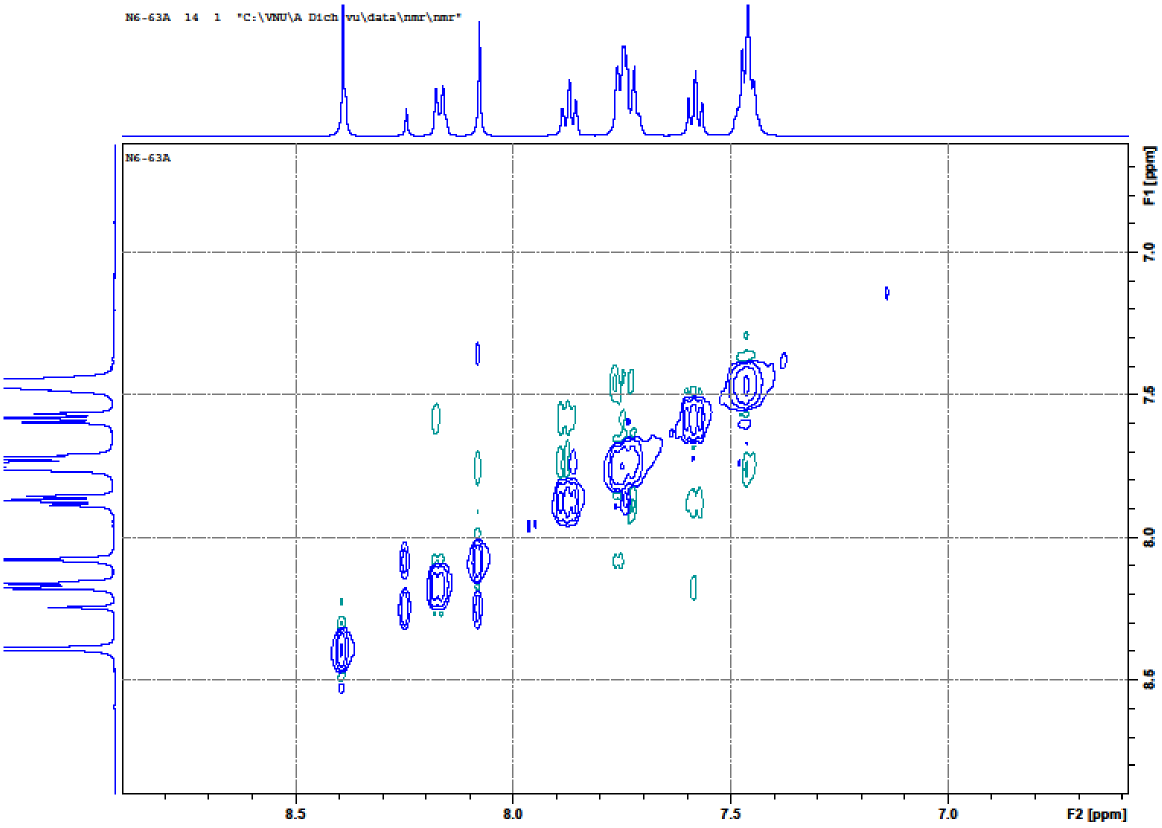

^{1}H NMR of compound 5b

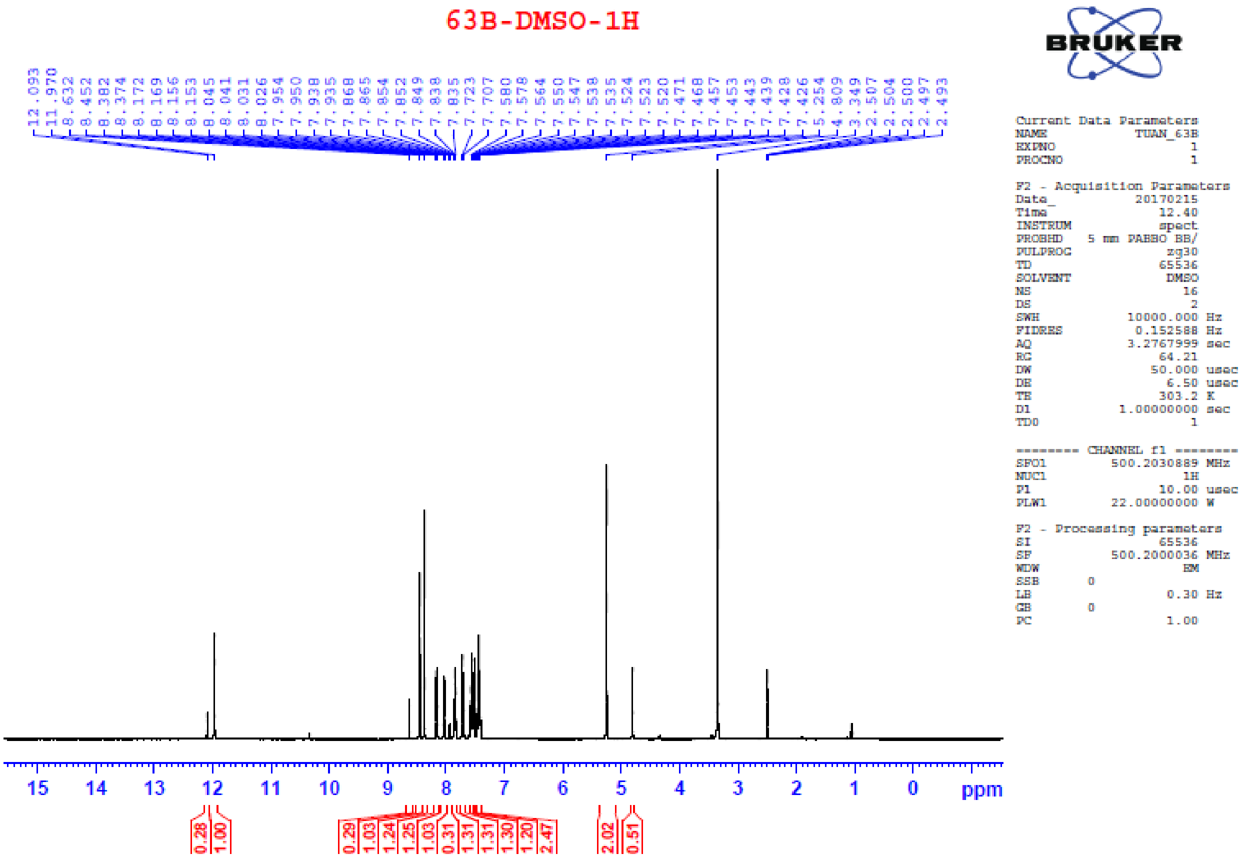

^{13}C NMR of compound 5b

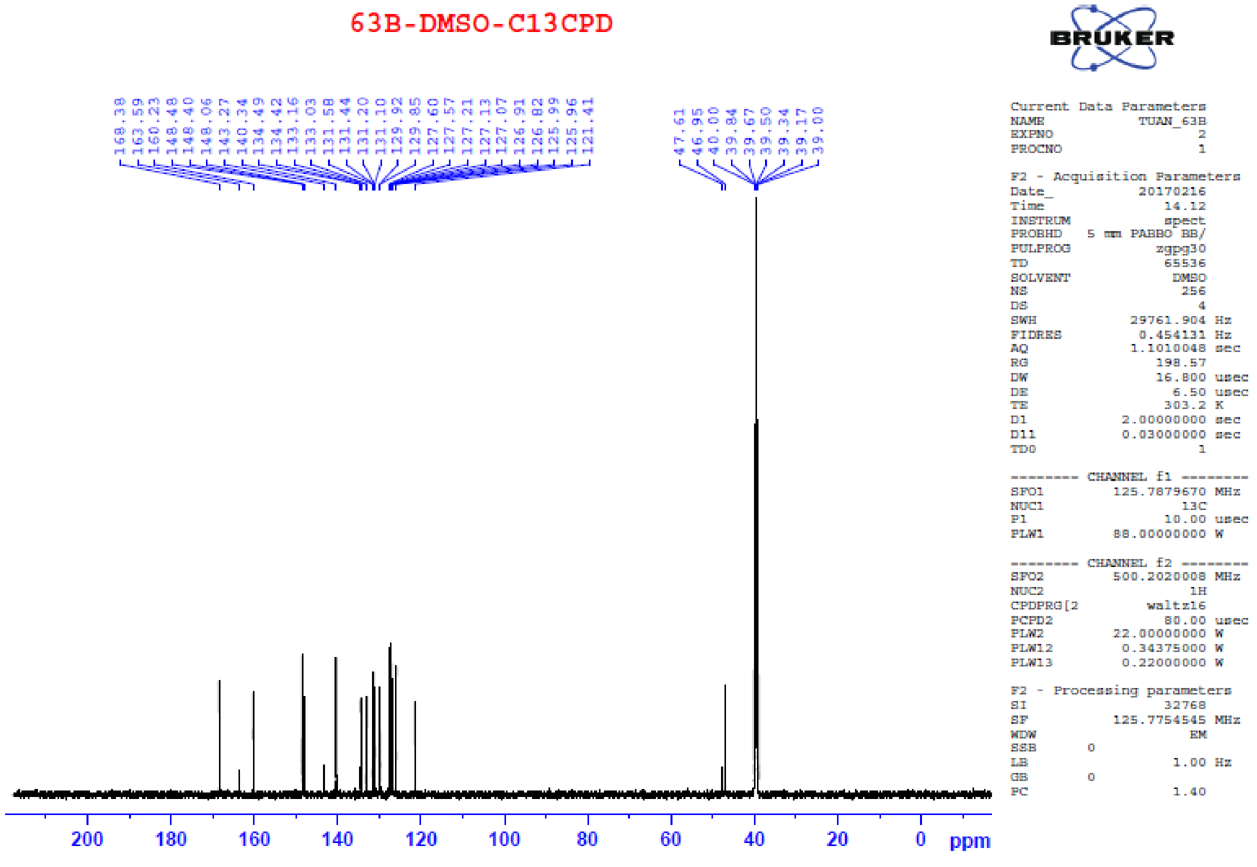

^{1}H NMR of compound 5c

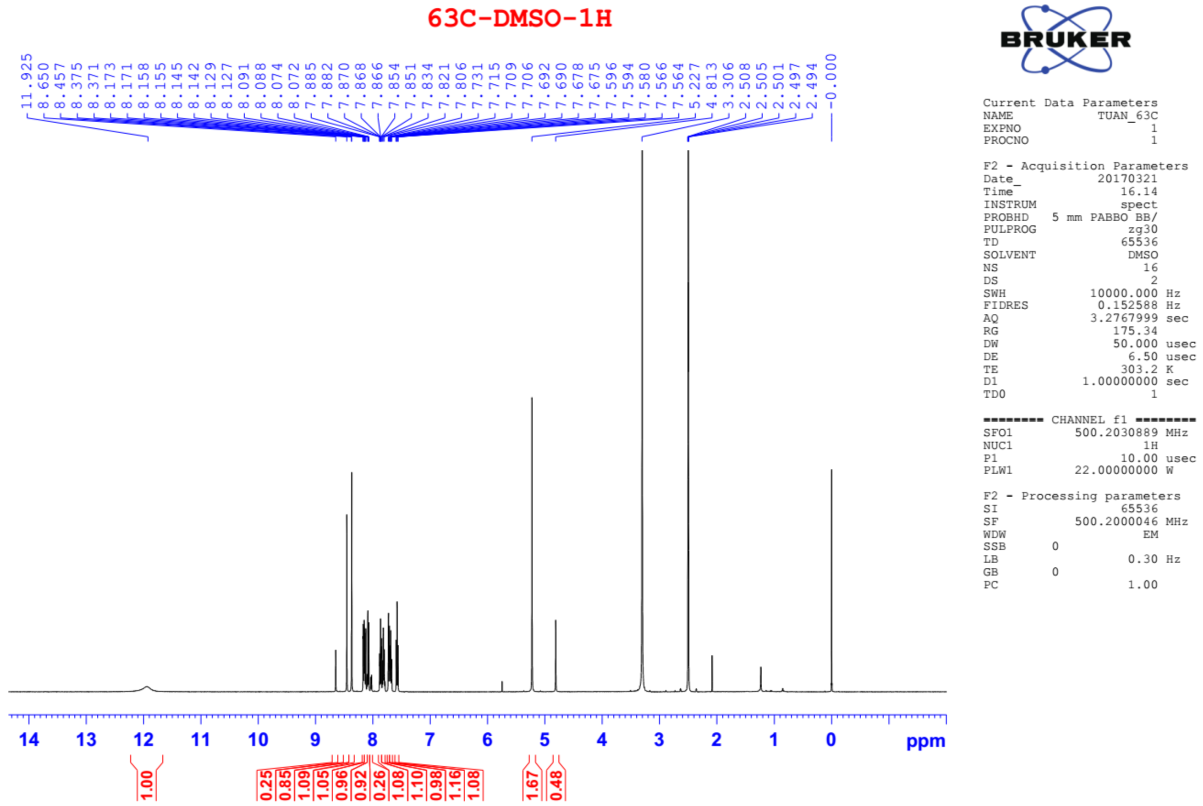

^{13}C NMR of compound 5c

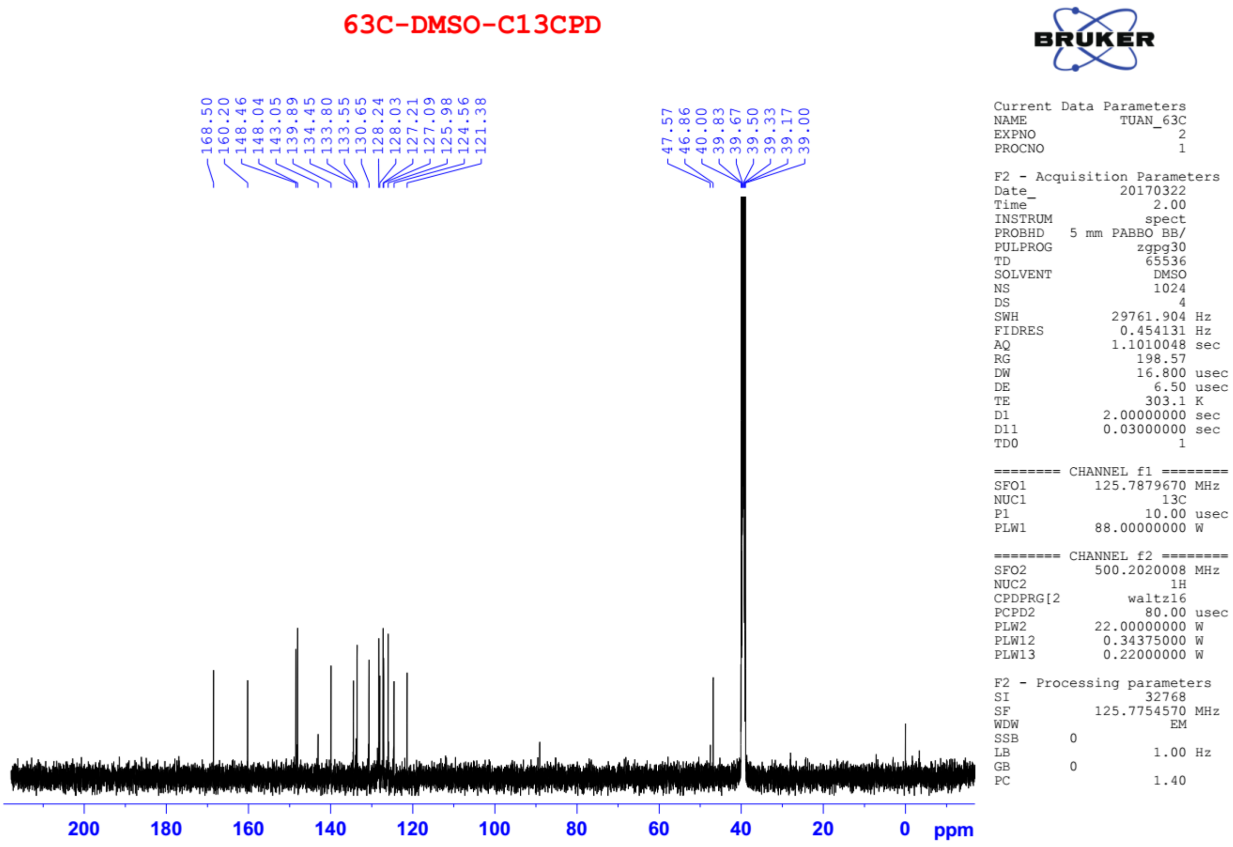

^{1}H NMR of compound 5d

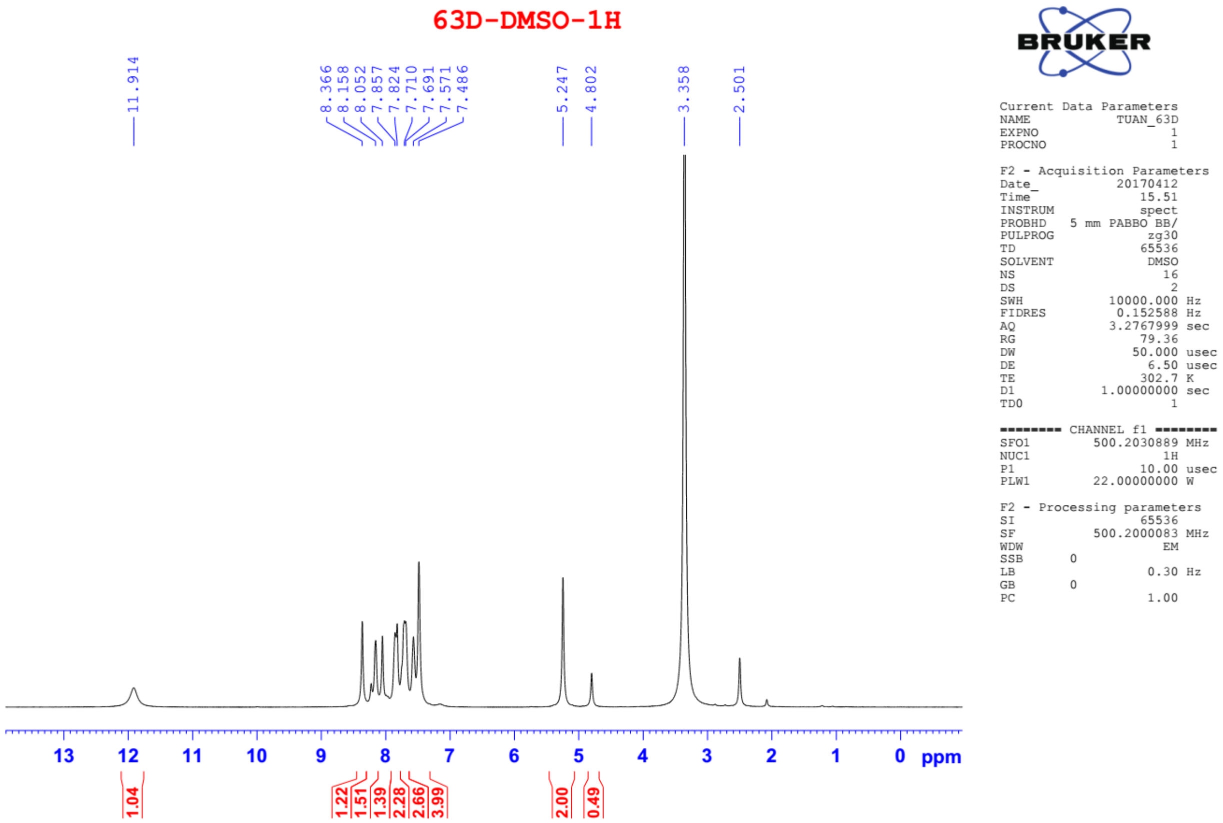

^{13}C NMR of compound 5d

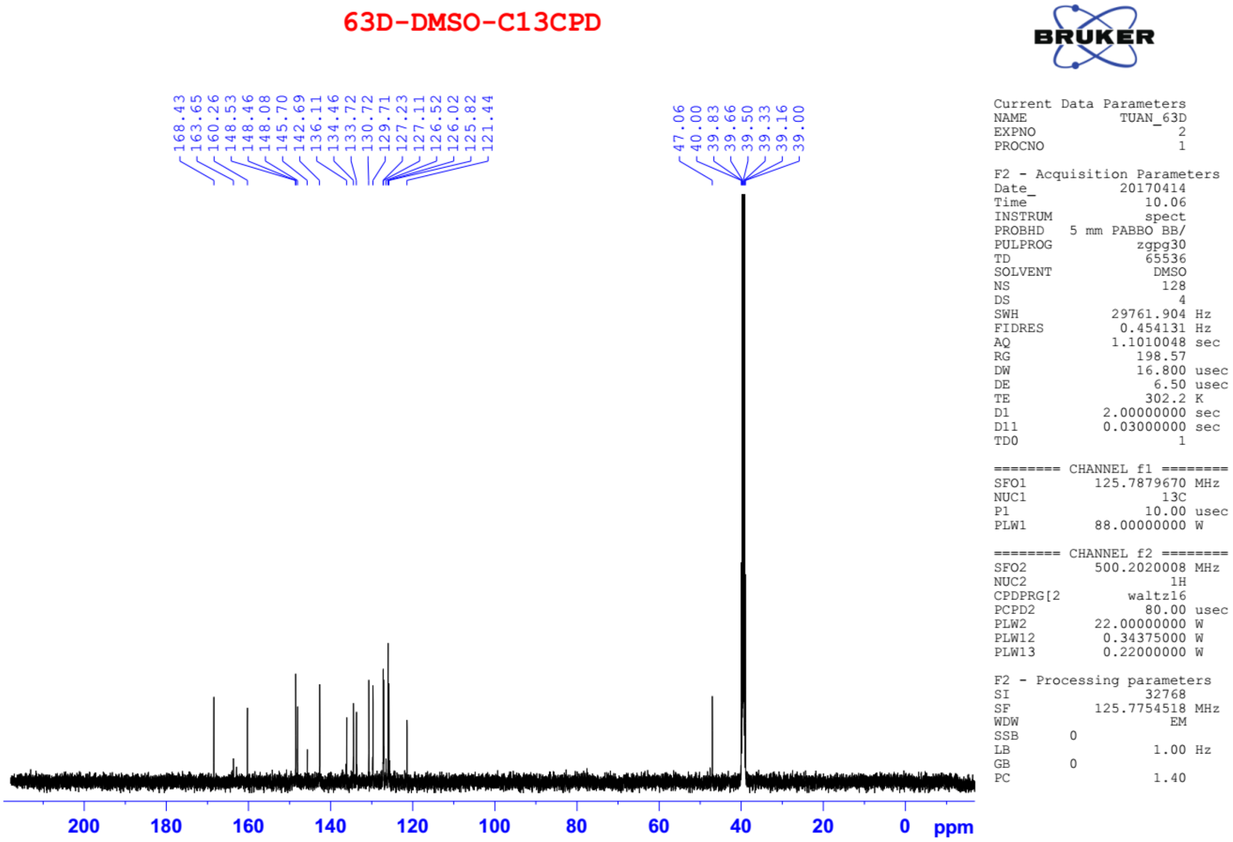

^{1}H NMR of compound 5e

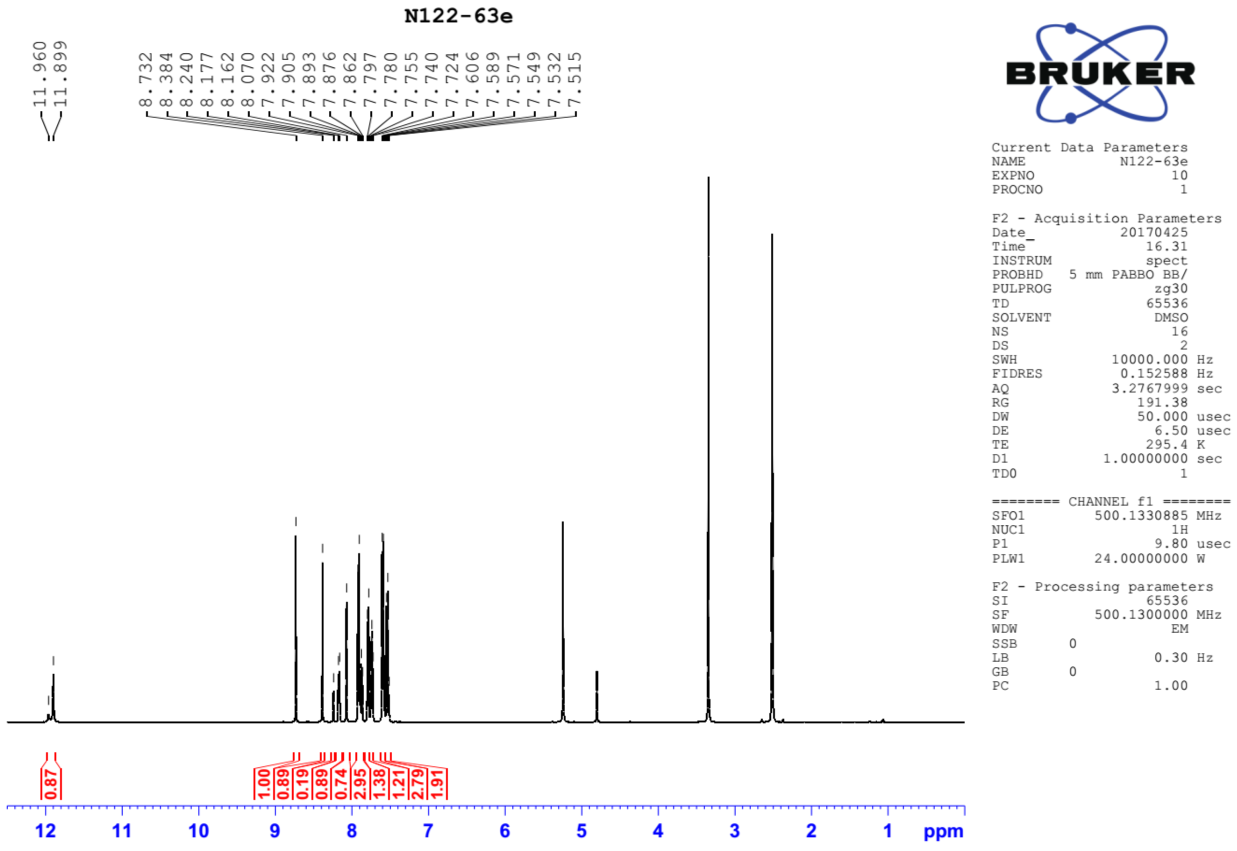

^{13}C NMR of compound 5e

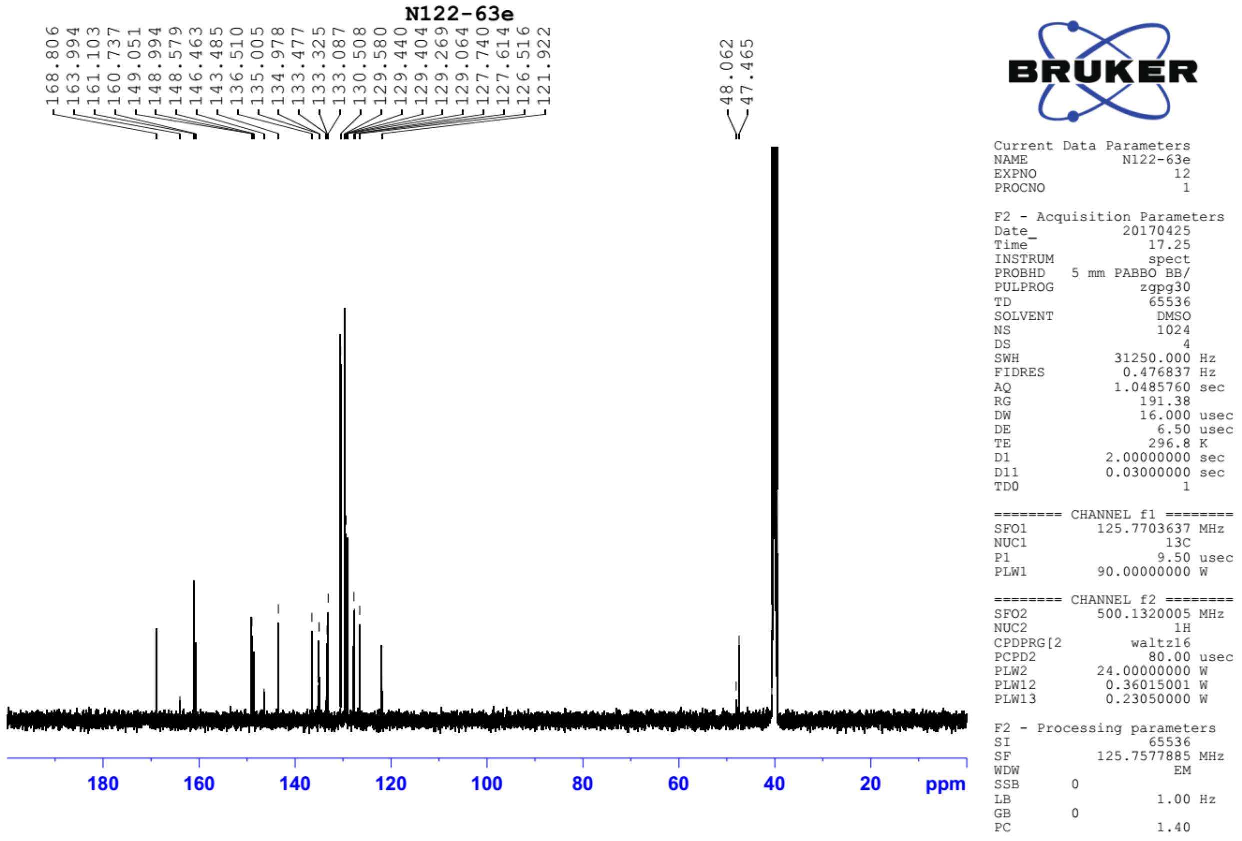

^{1}H NMR of compound 5f

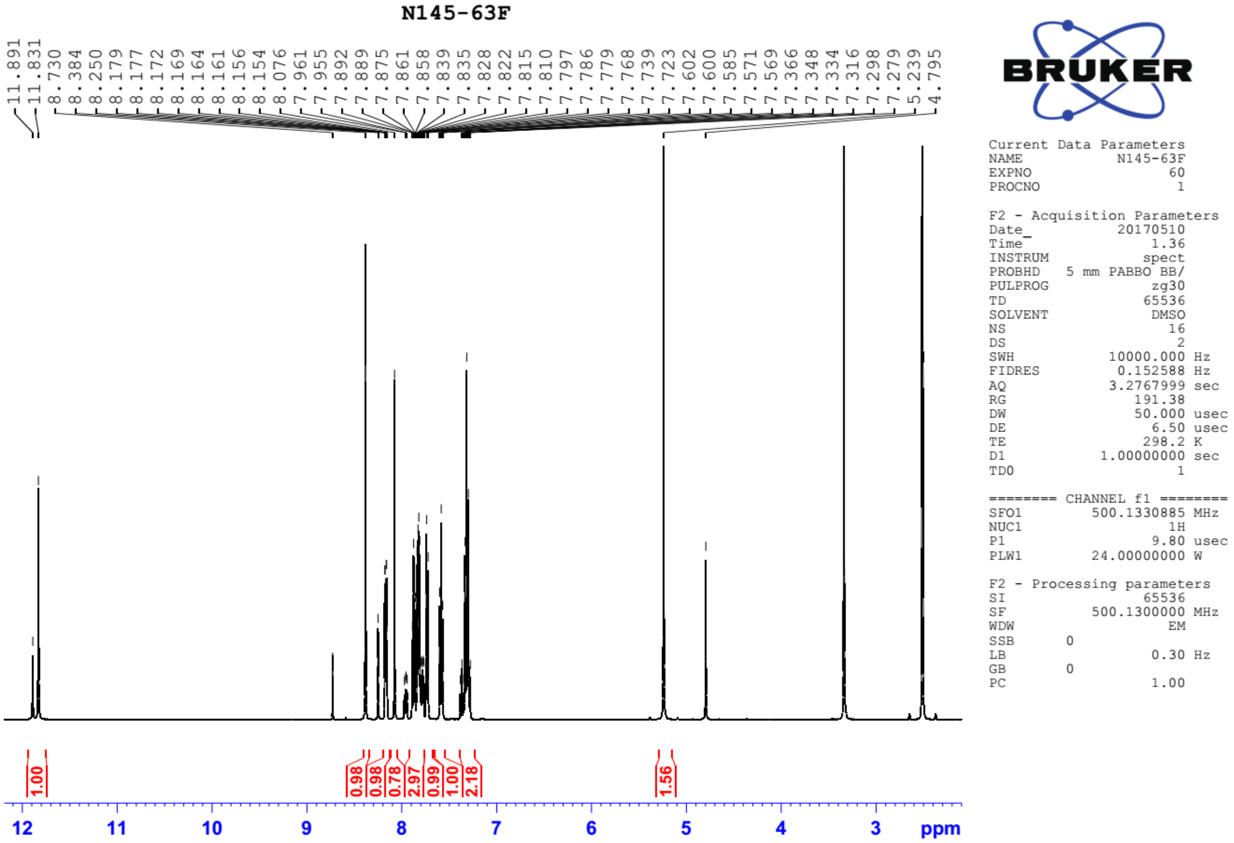

^{13}C NMR of compound 5f

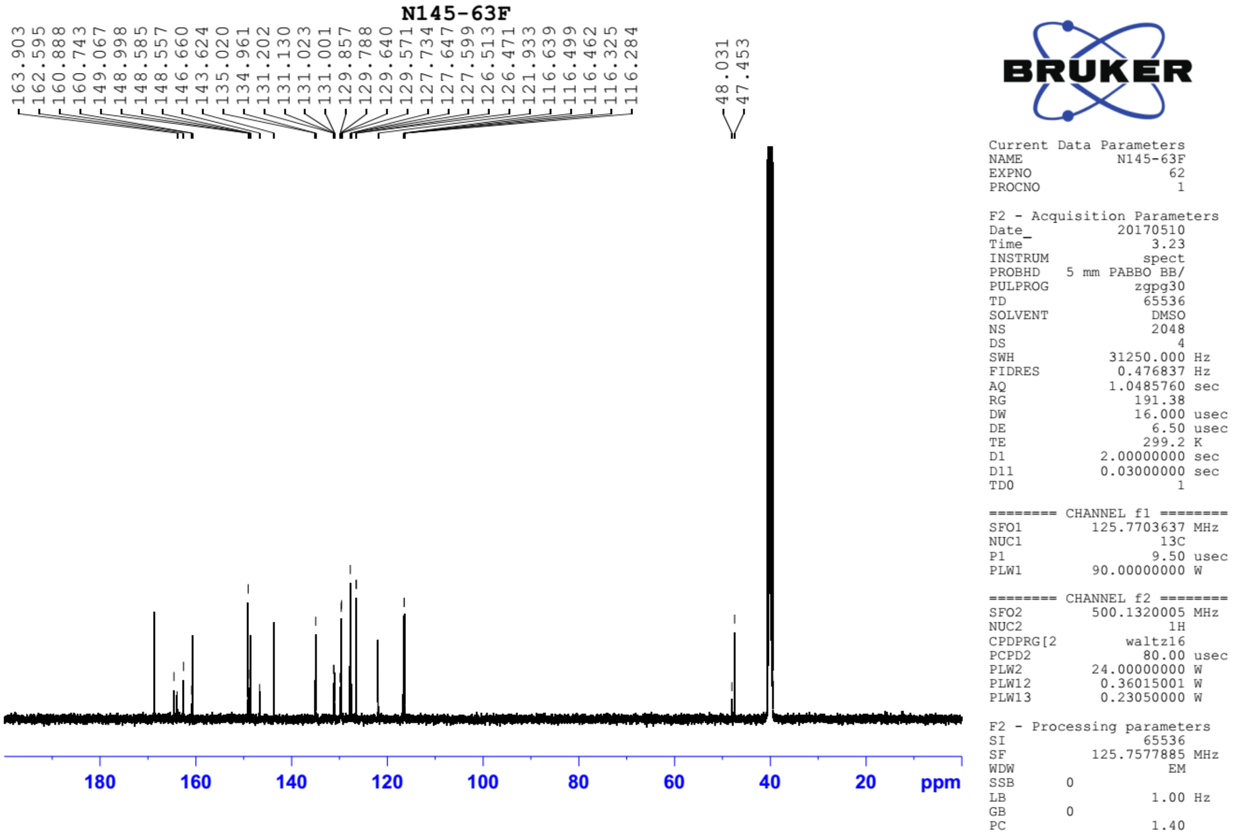

^{1}H NMR of compound 5g

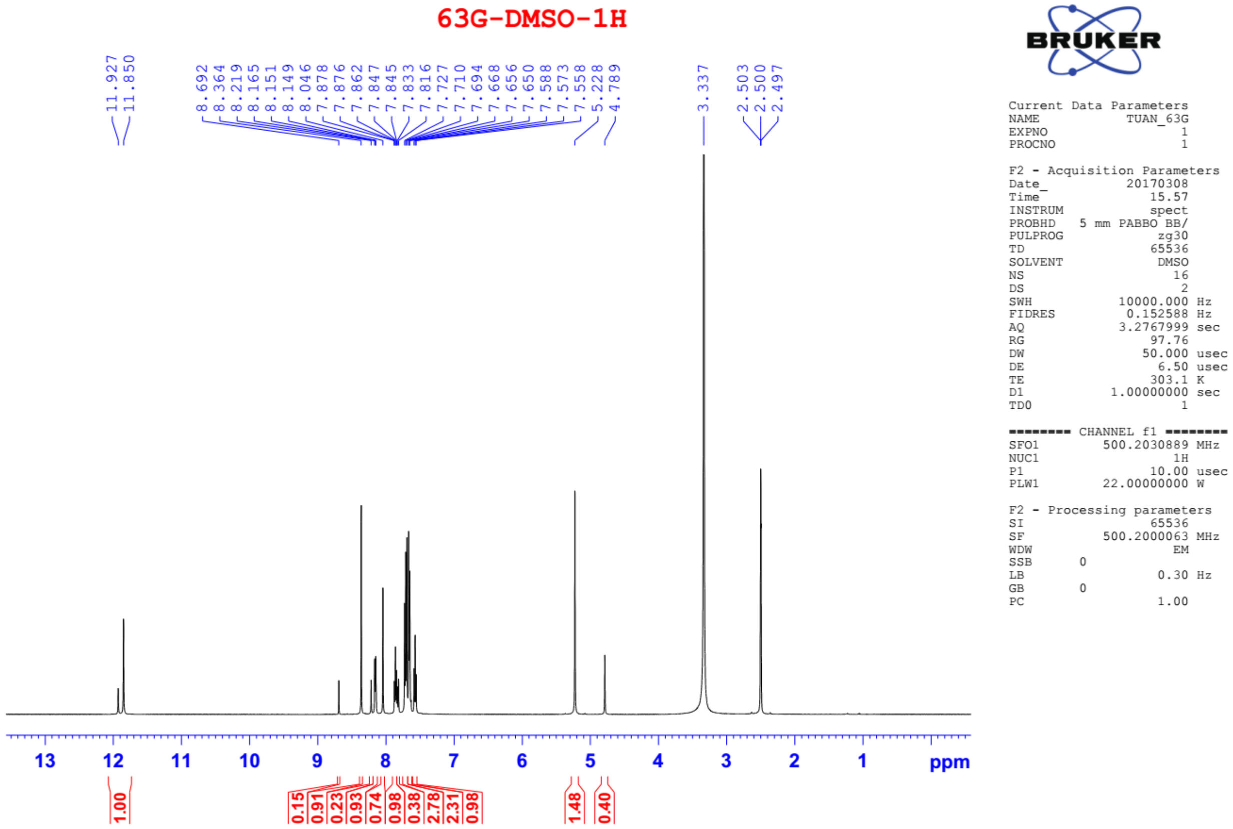

^{13}C NMR of compound 5g

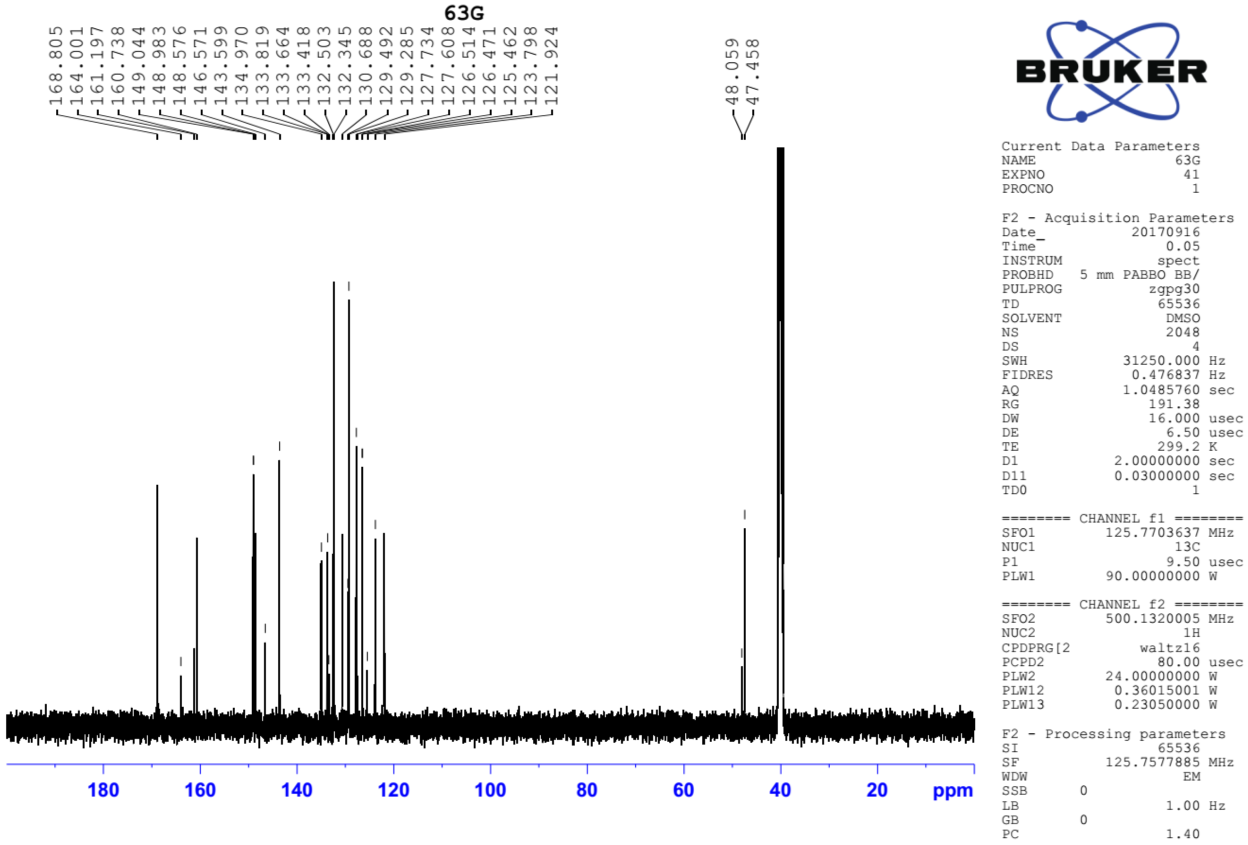

^{1}H NMR of compound 5h

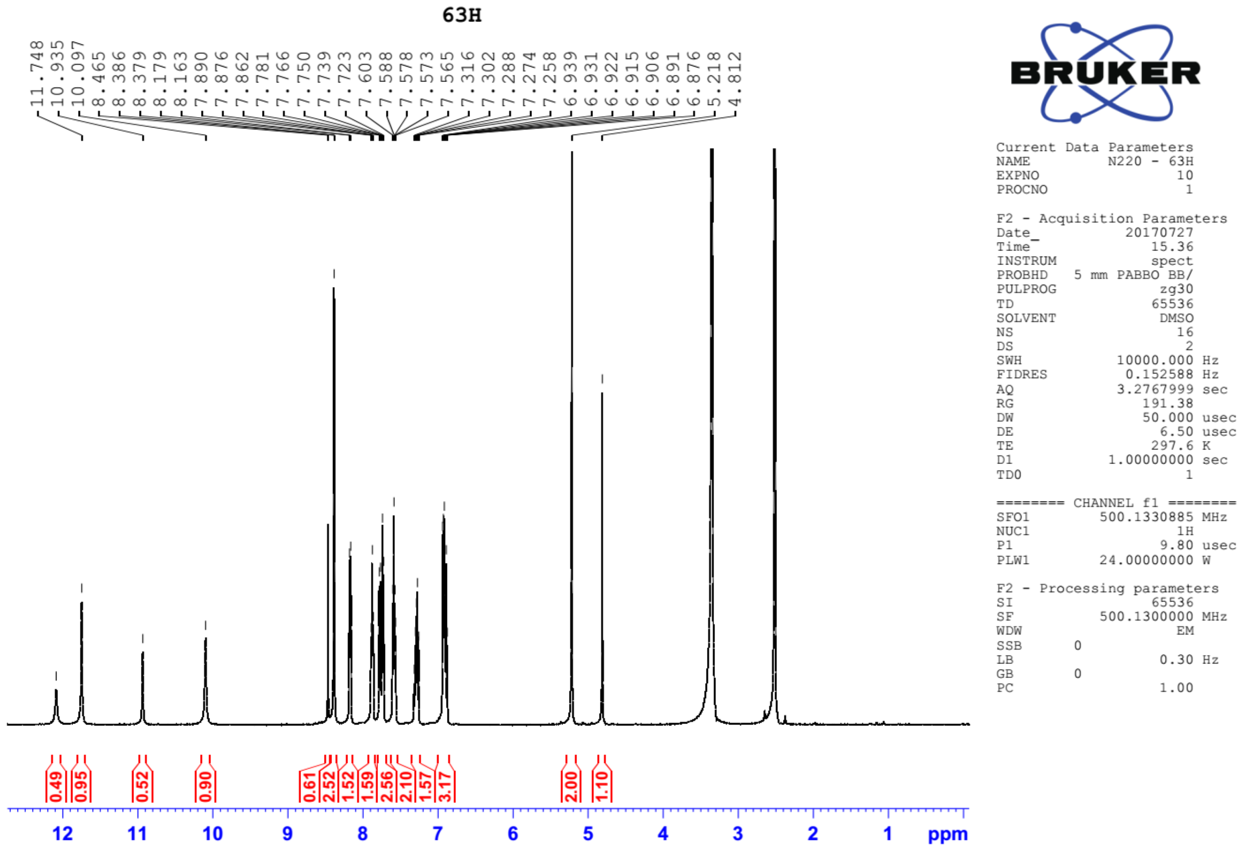

^{13}C NMR of compound 5h

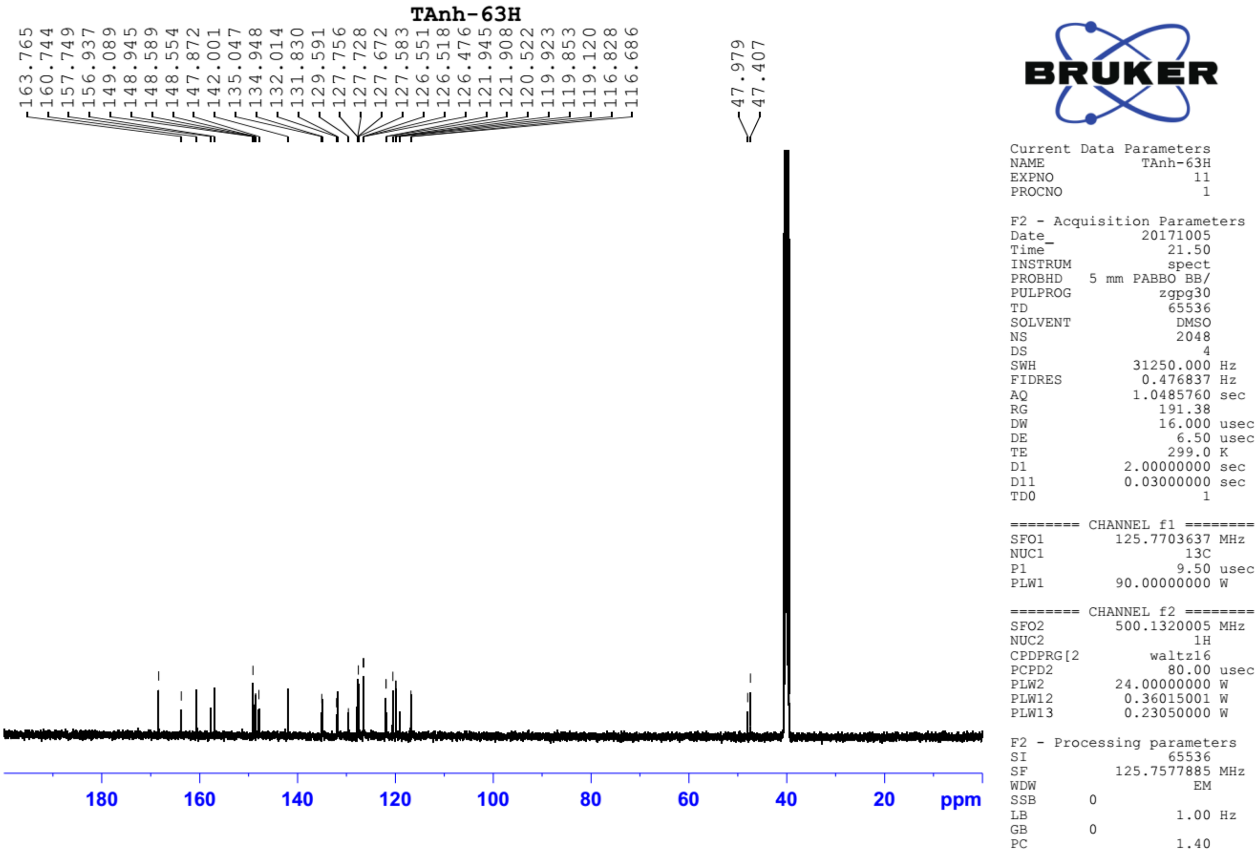

^{1}H NMR of compound 5i

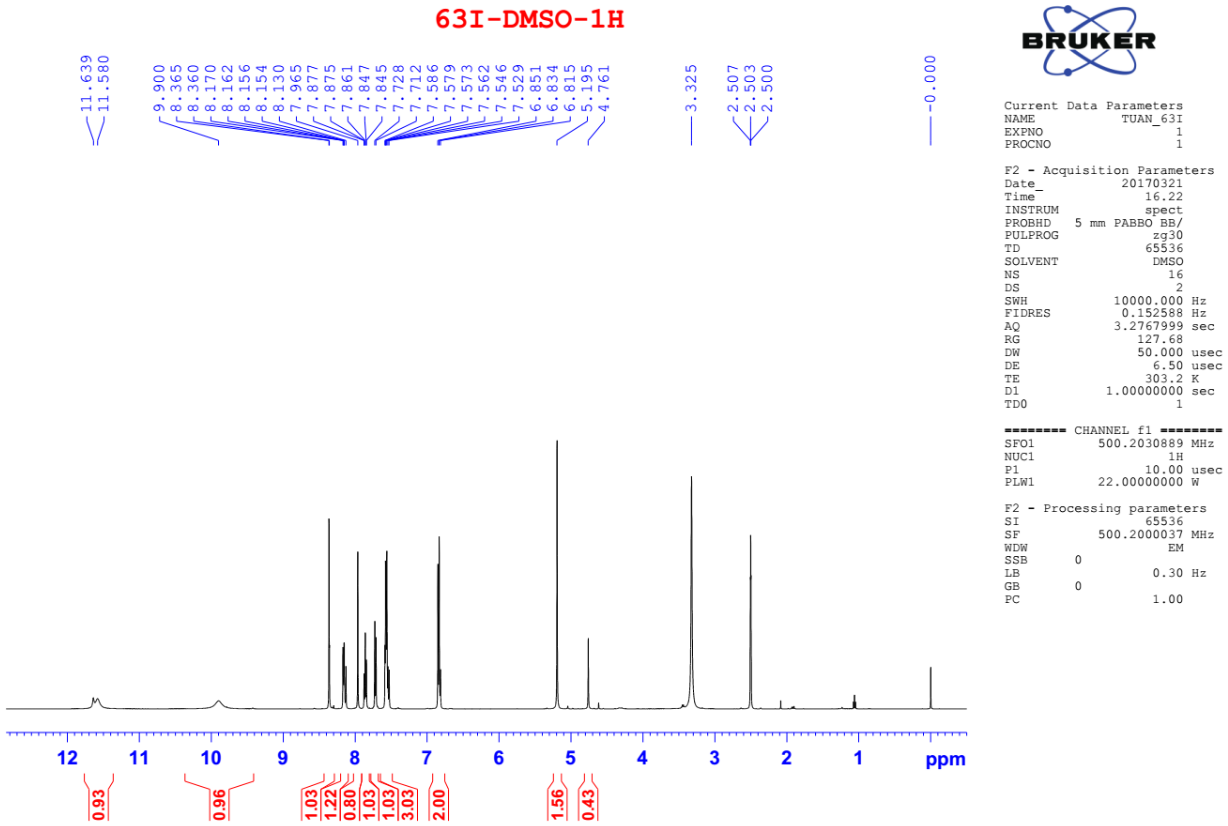

^{13}C NMR of compound 5i

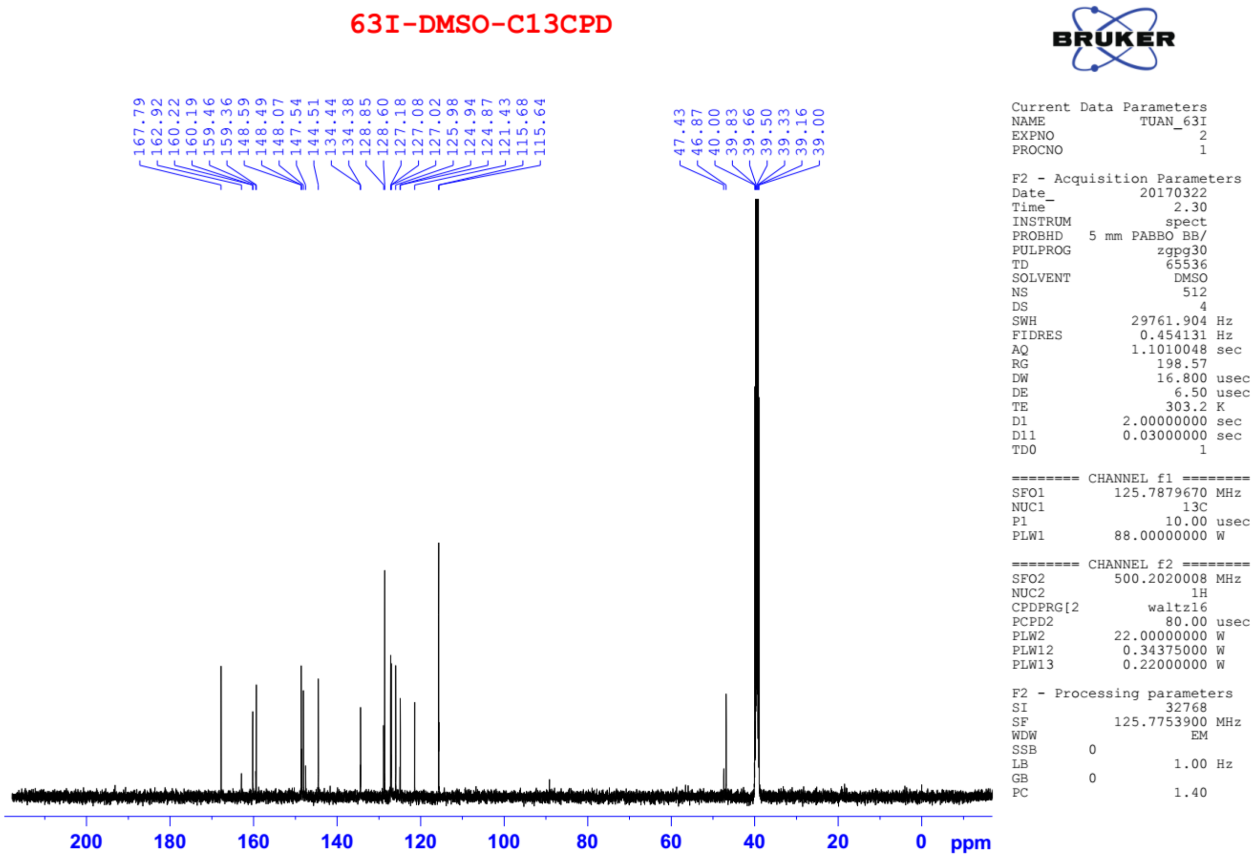

^{1}H NMR of compound 5j

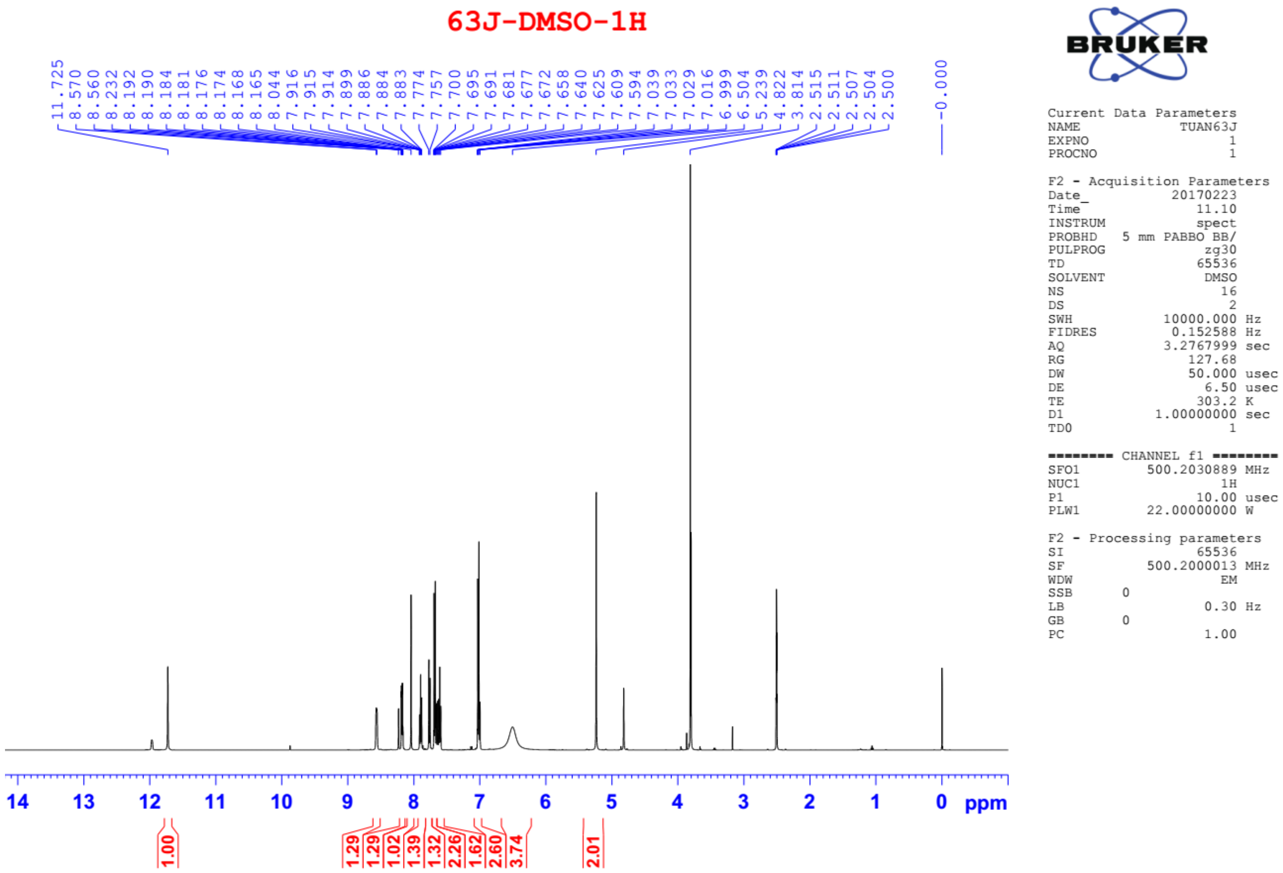

¹³C NMR of compound 5j

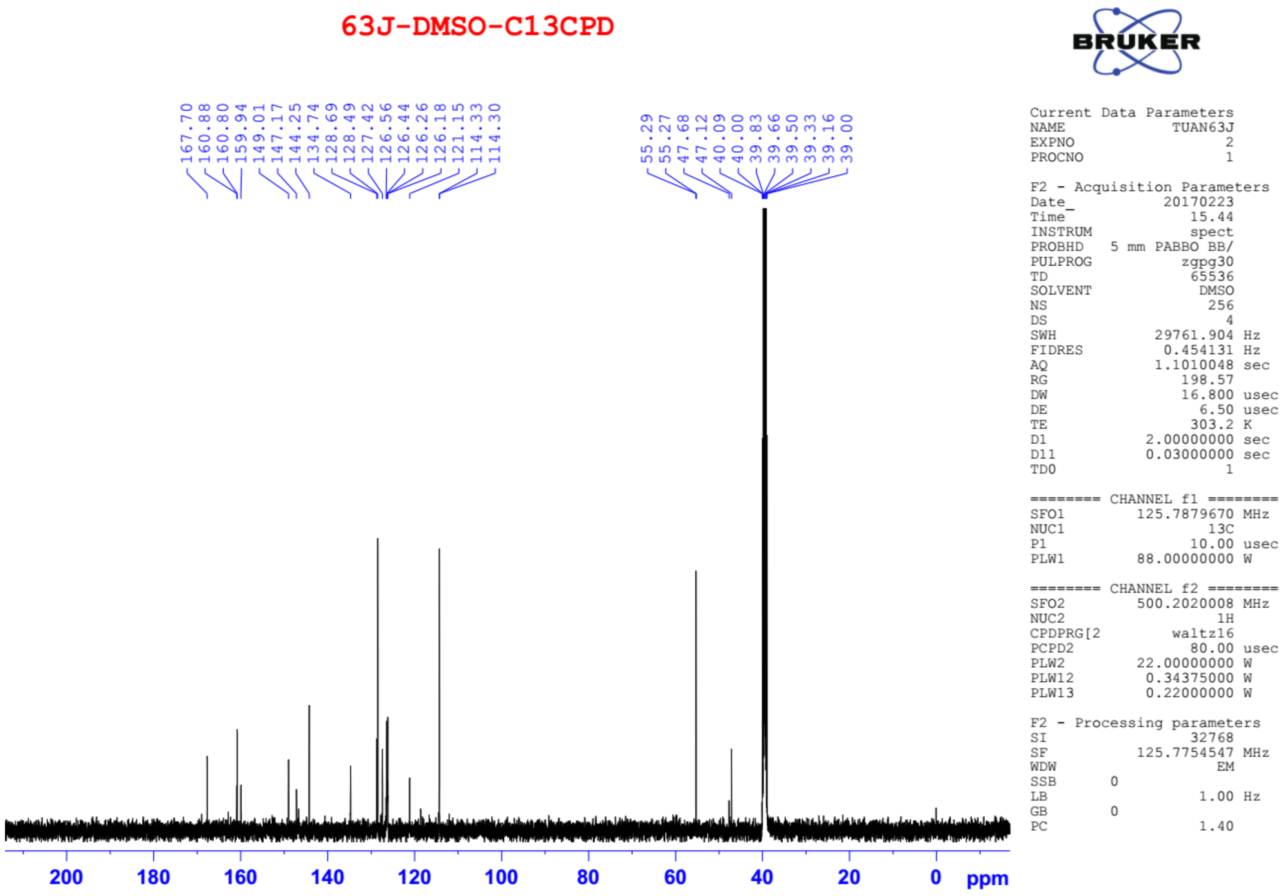

¹H NMR of compound 5k

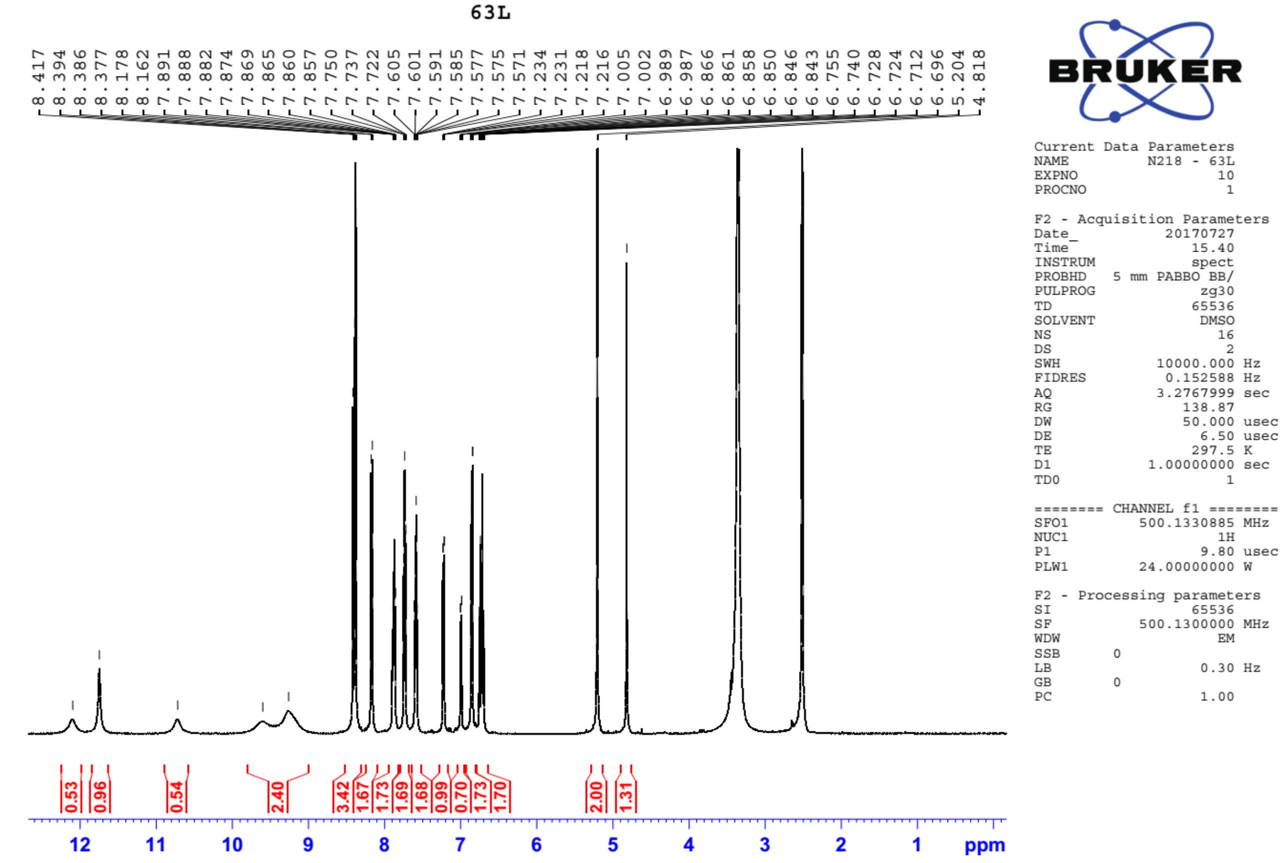

^{13}C NMR of compound 5k

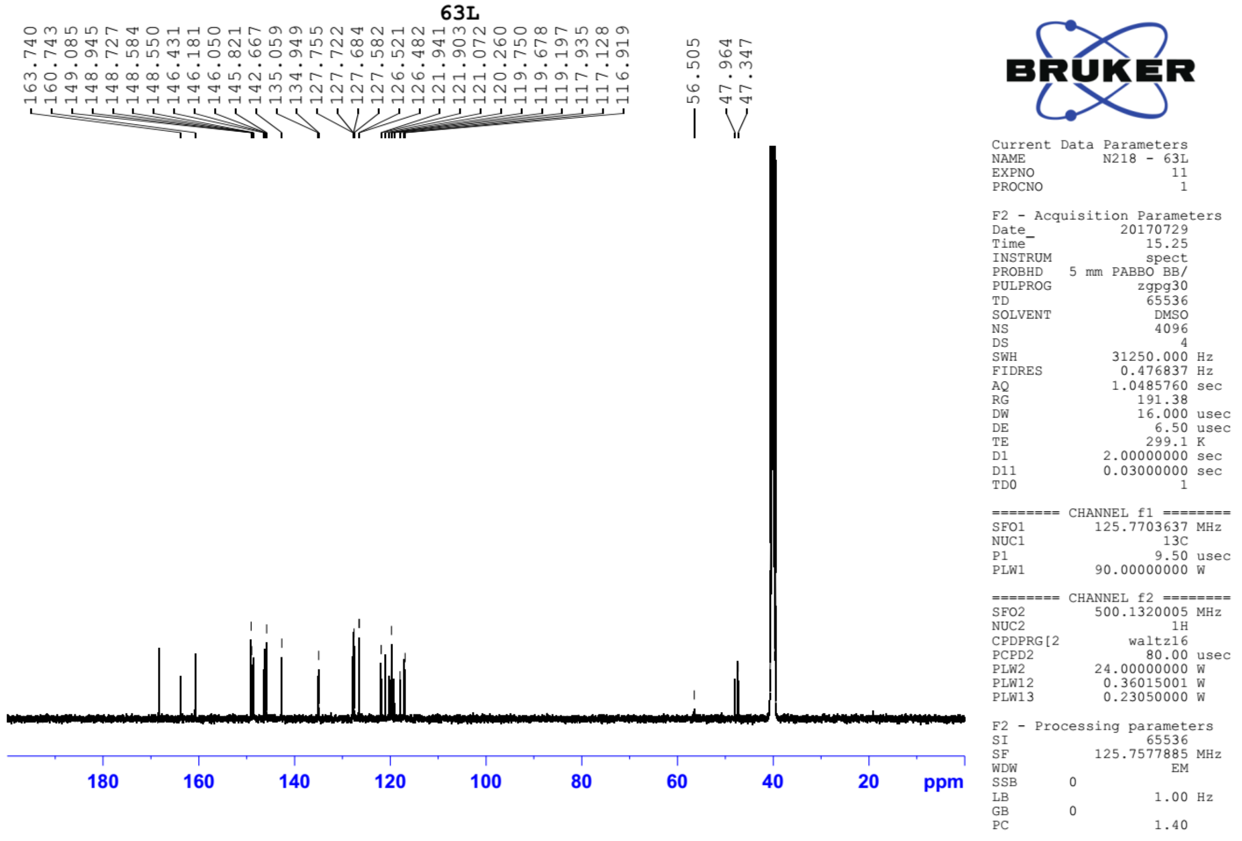

^{1}H NMR of compound 5l

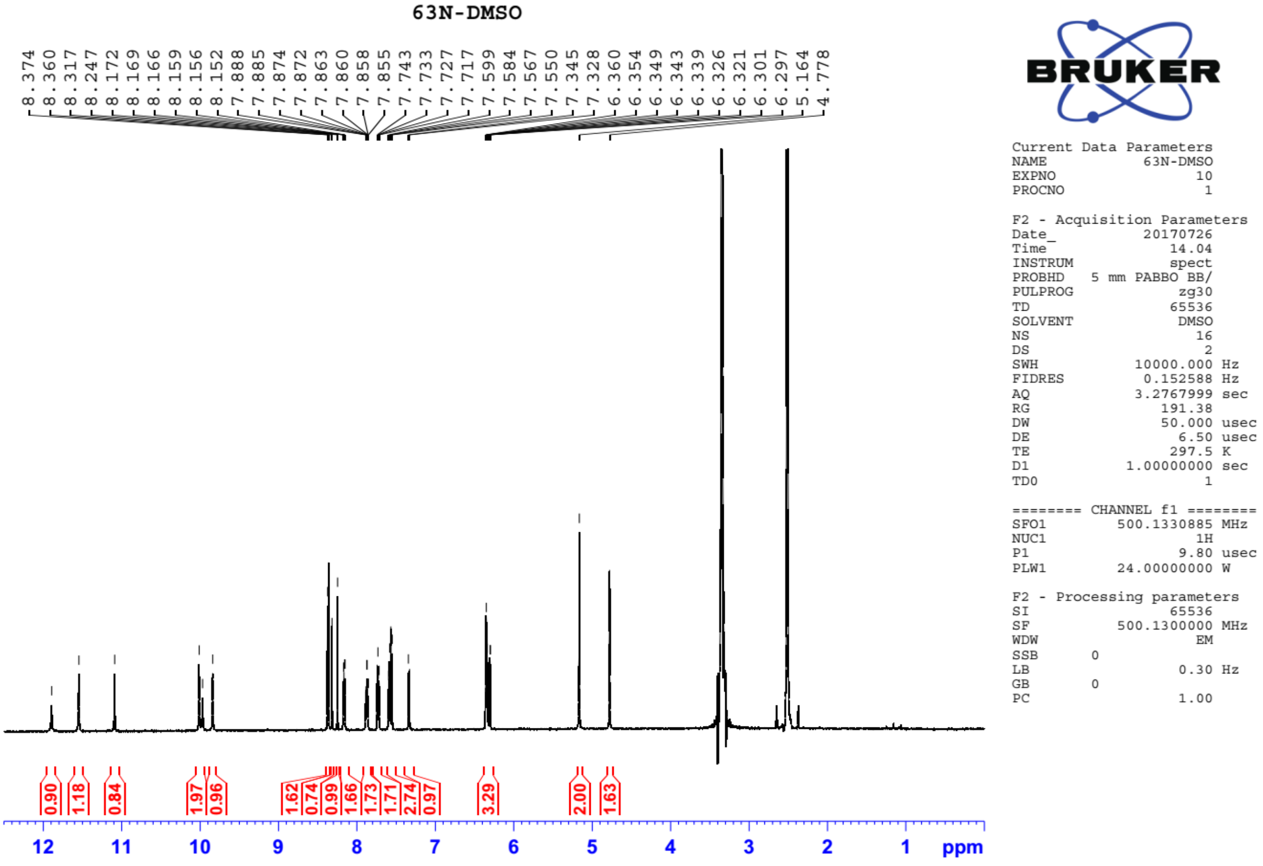

^{13}C NMR of compound 5l

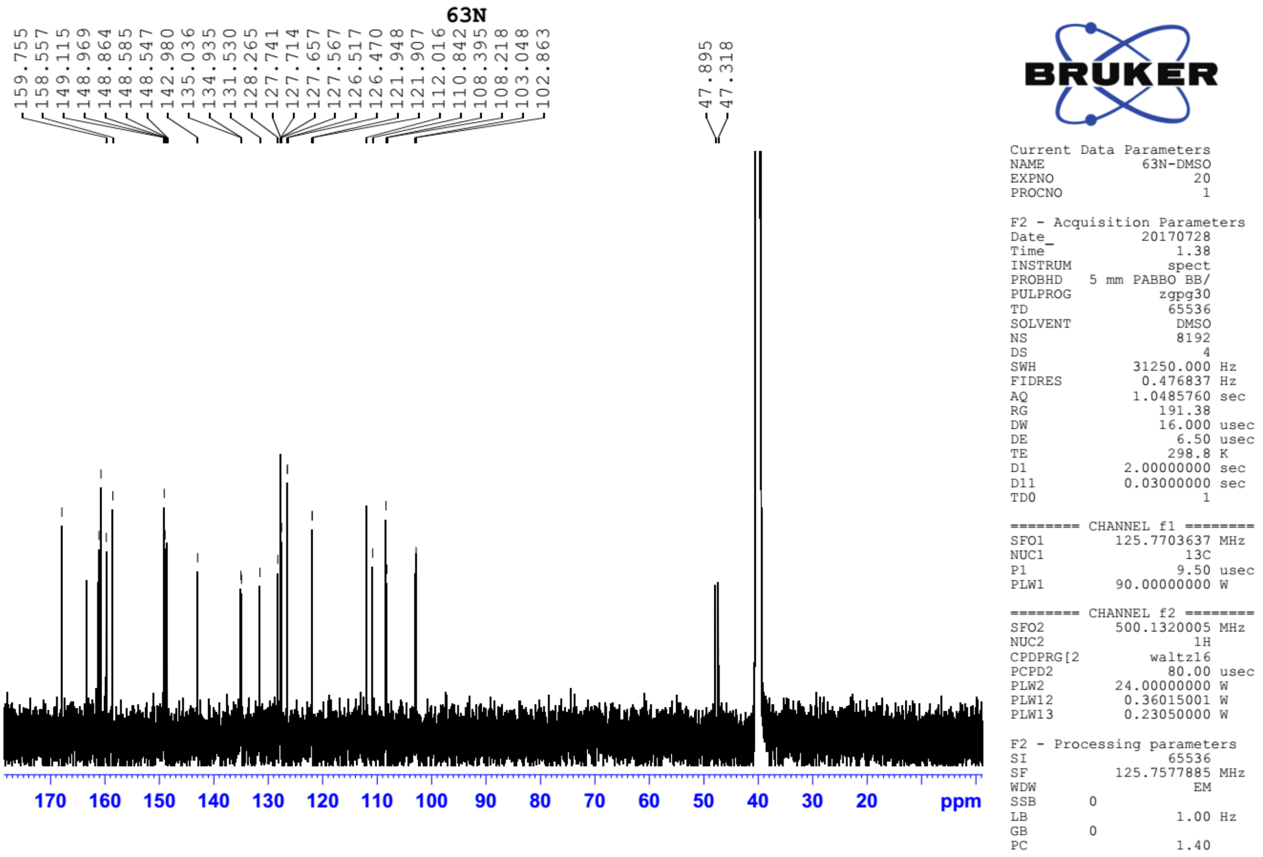

^{1}H NMR of compound 5m

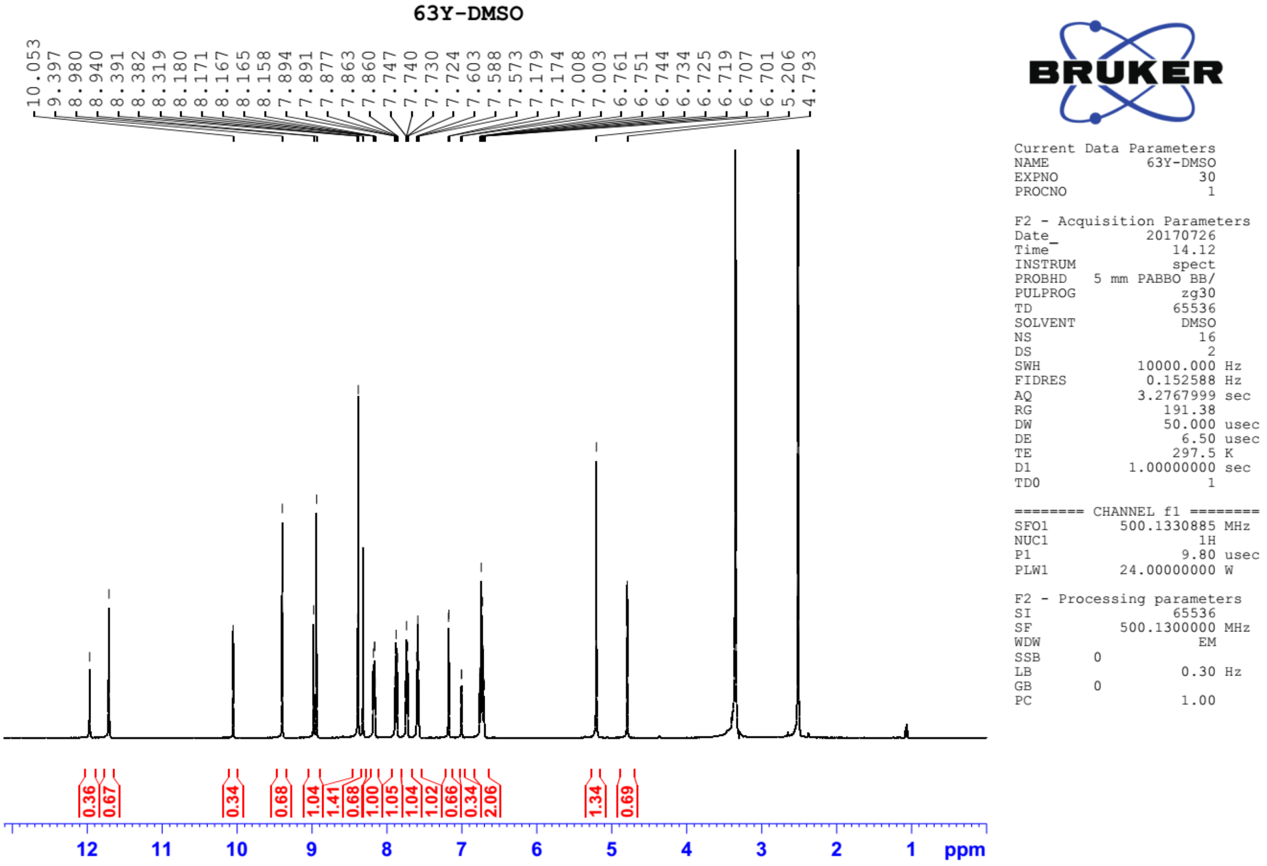

^{13}C NMR of compound 5m

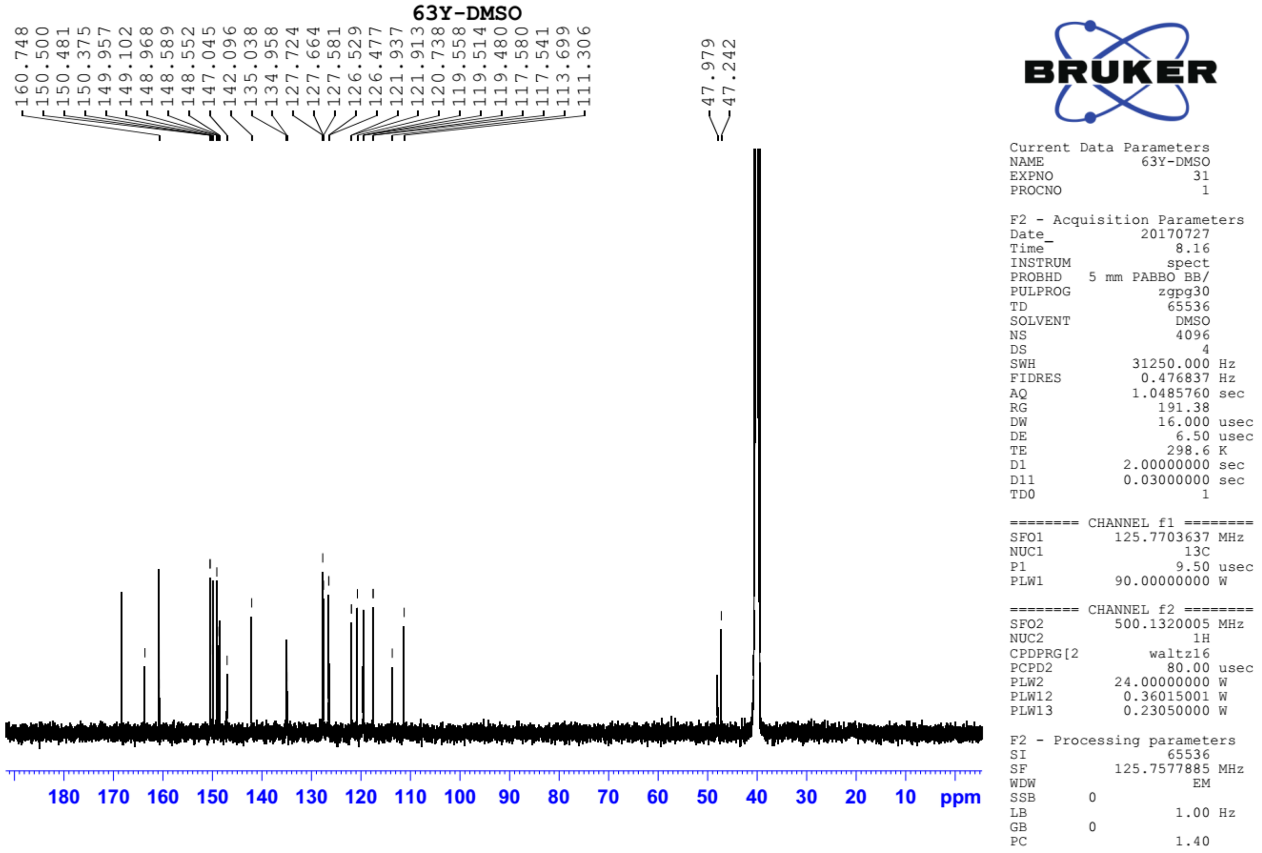

^{1}H NMR of compound 5n

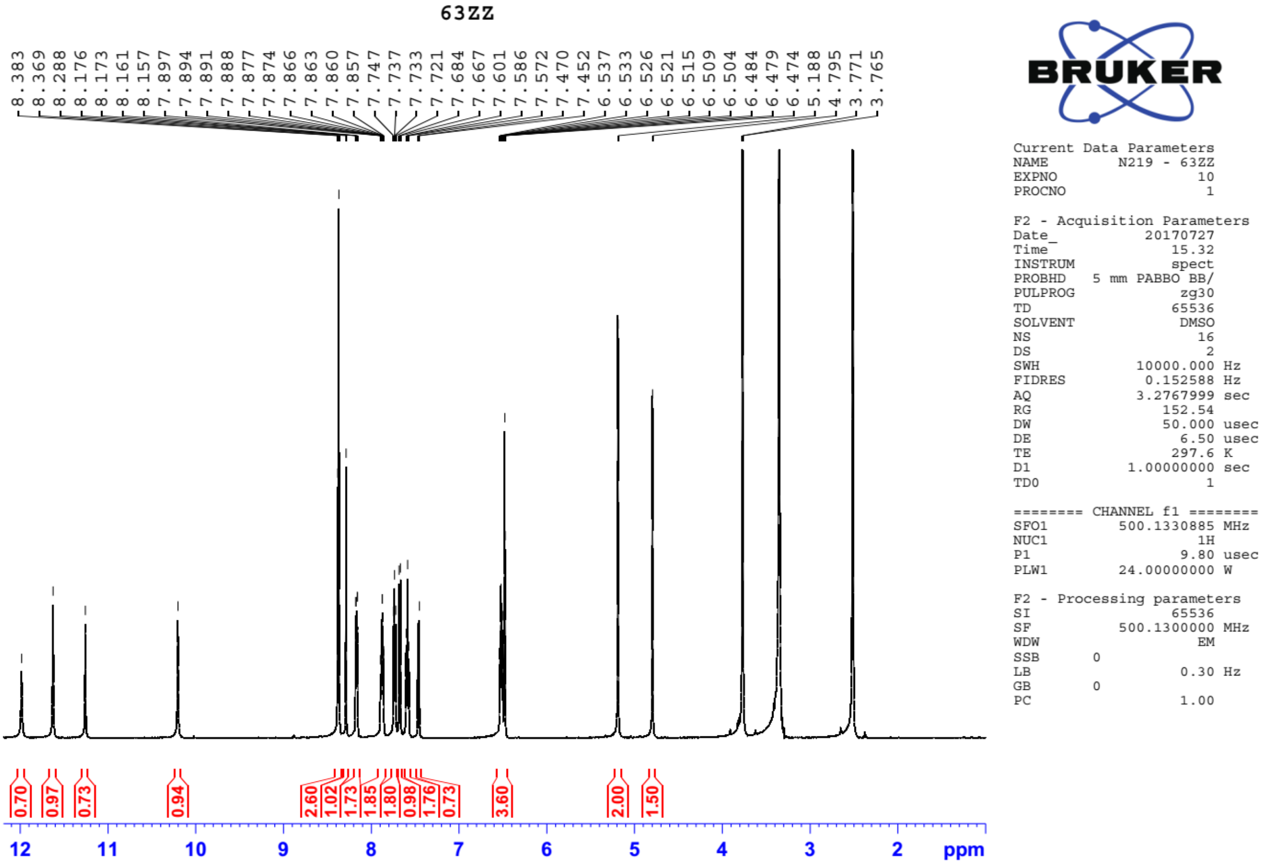

^{13}C NMR of compound 5n

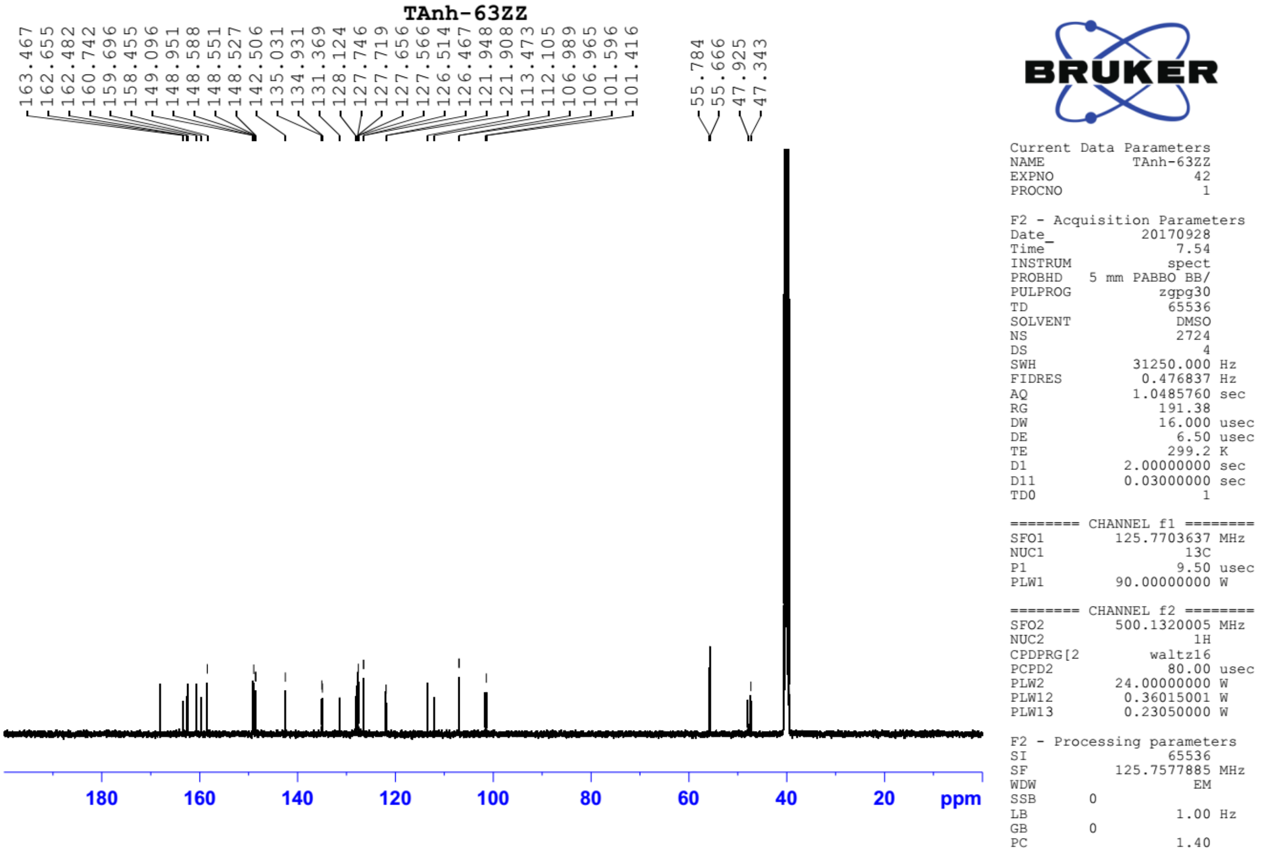

^{1}H NMR of compound 5o

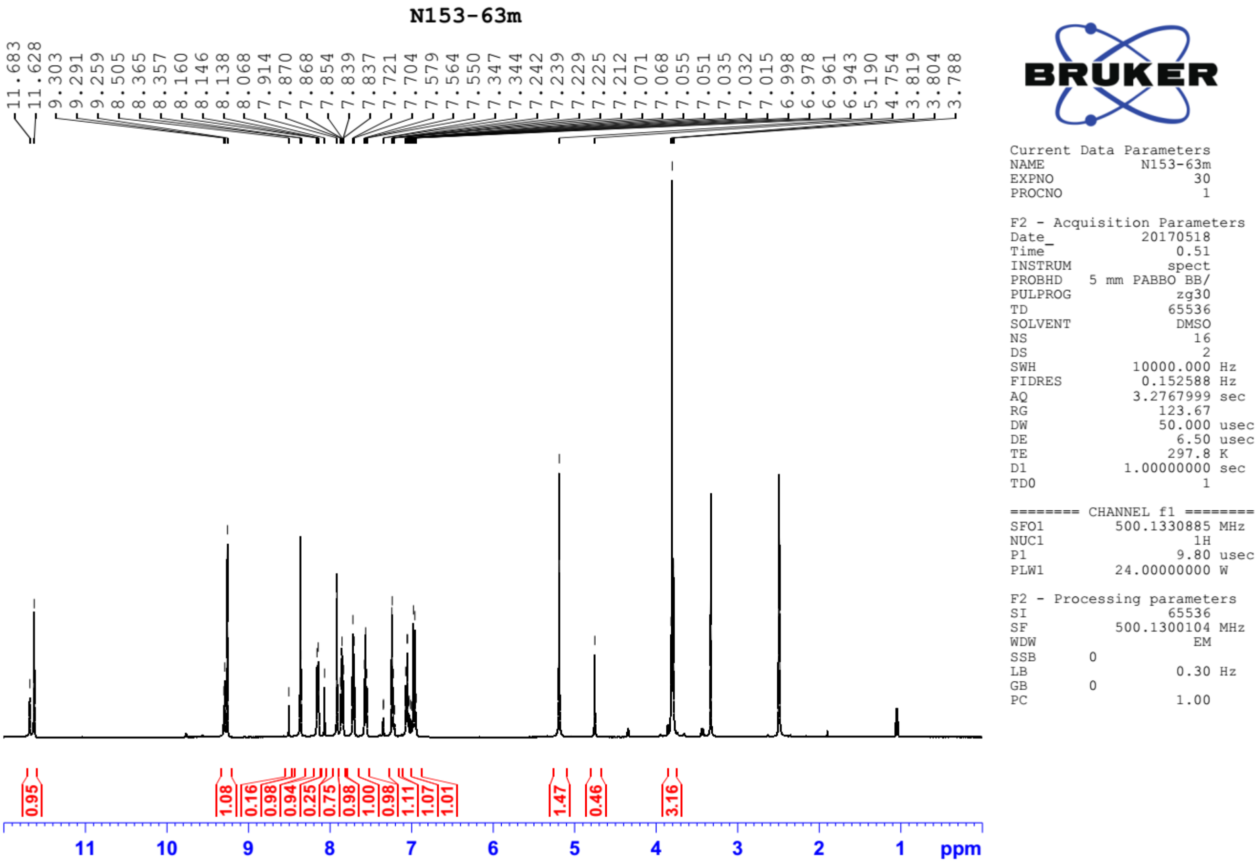

^{13}C NMR of compound 5o

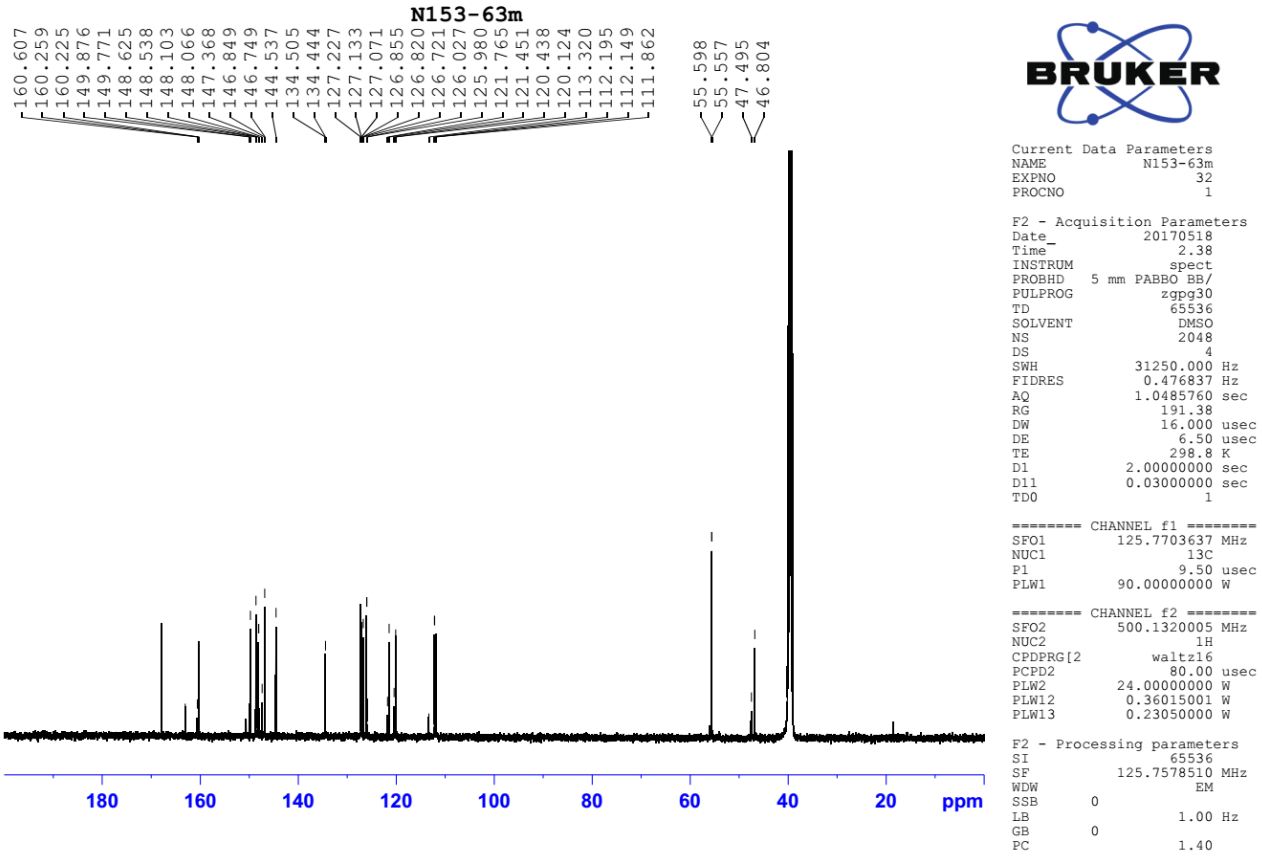

^{1}H NMR of compound 5p

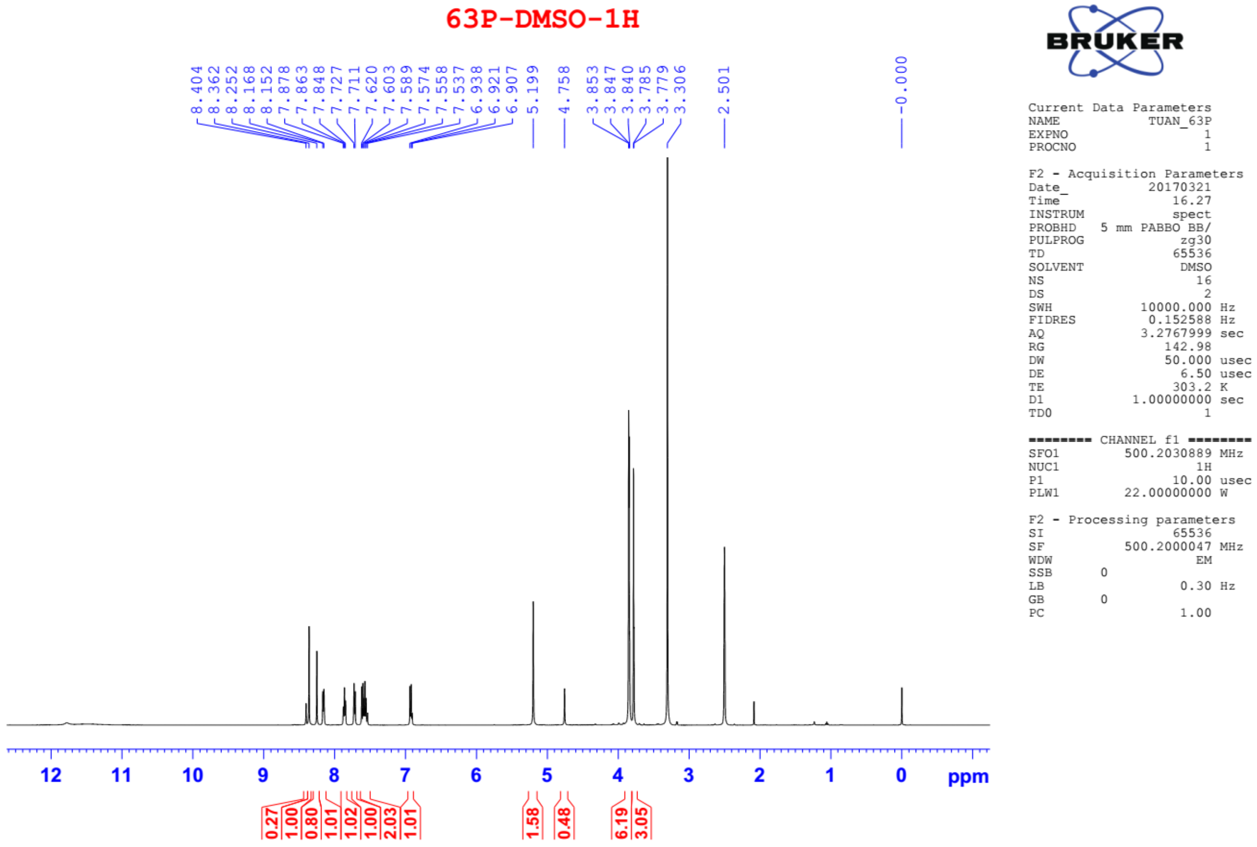

^{13}C NMR of compound 5p

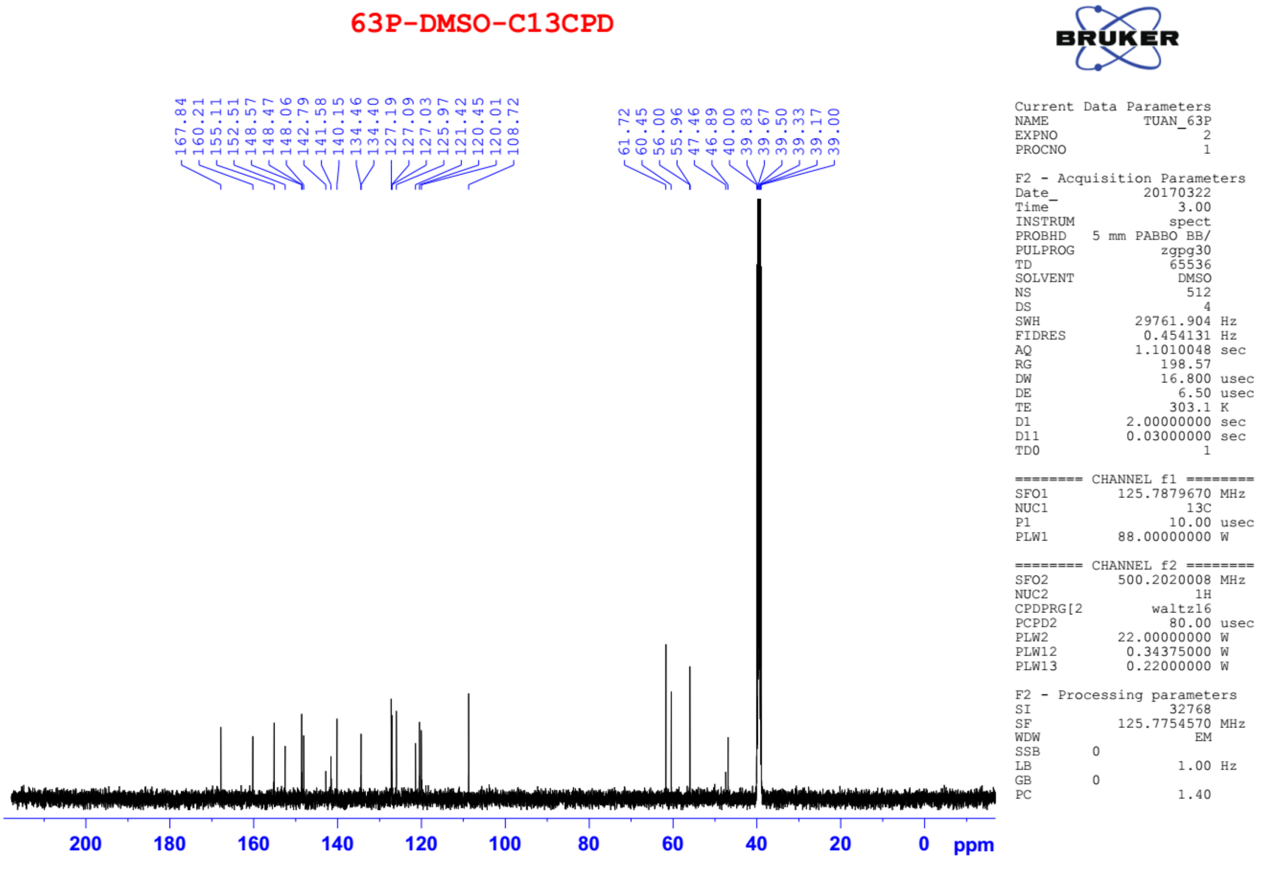

^{1}H NMR of compound 5q

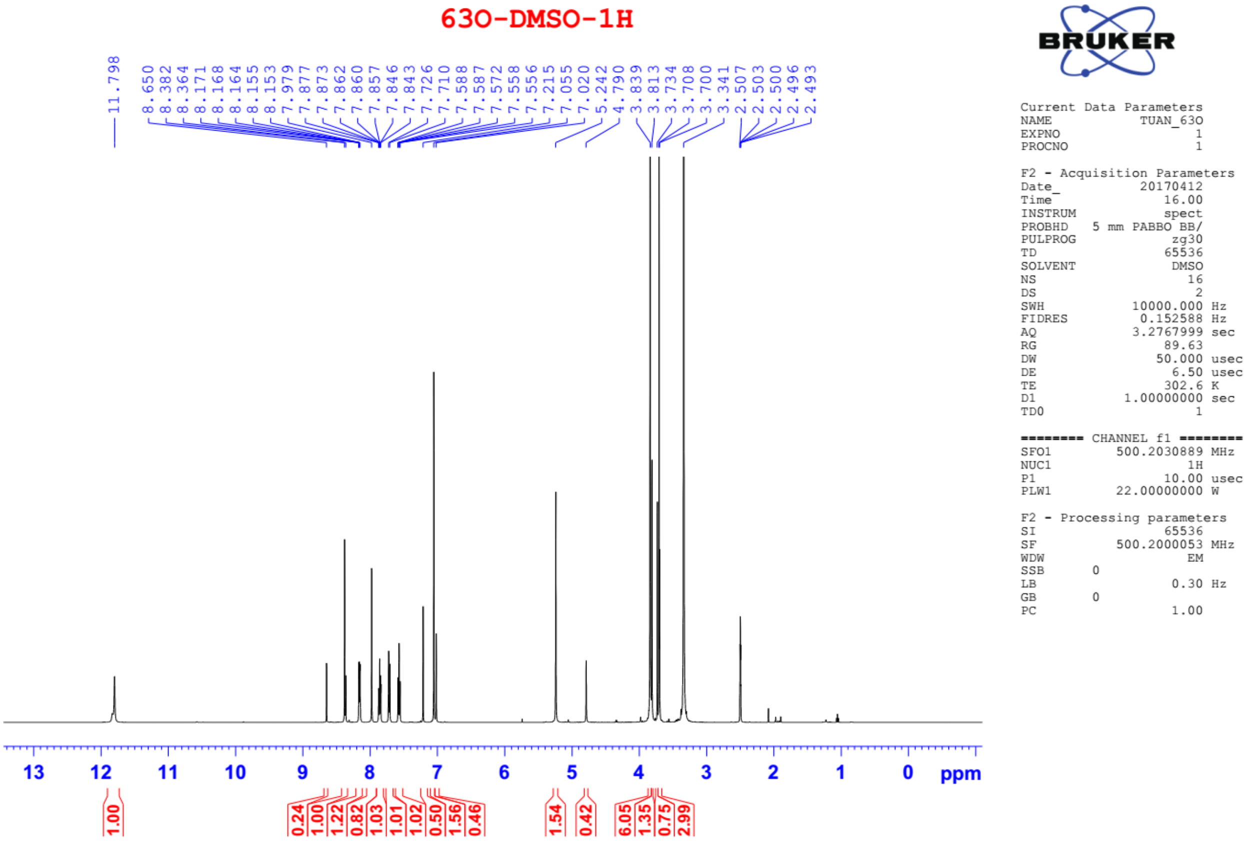

^{13}C NMR of compound 5q

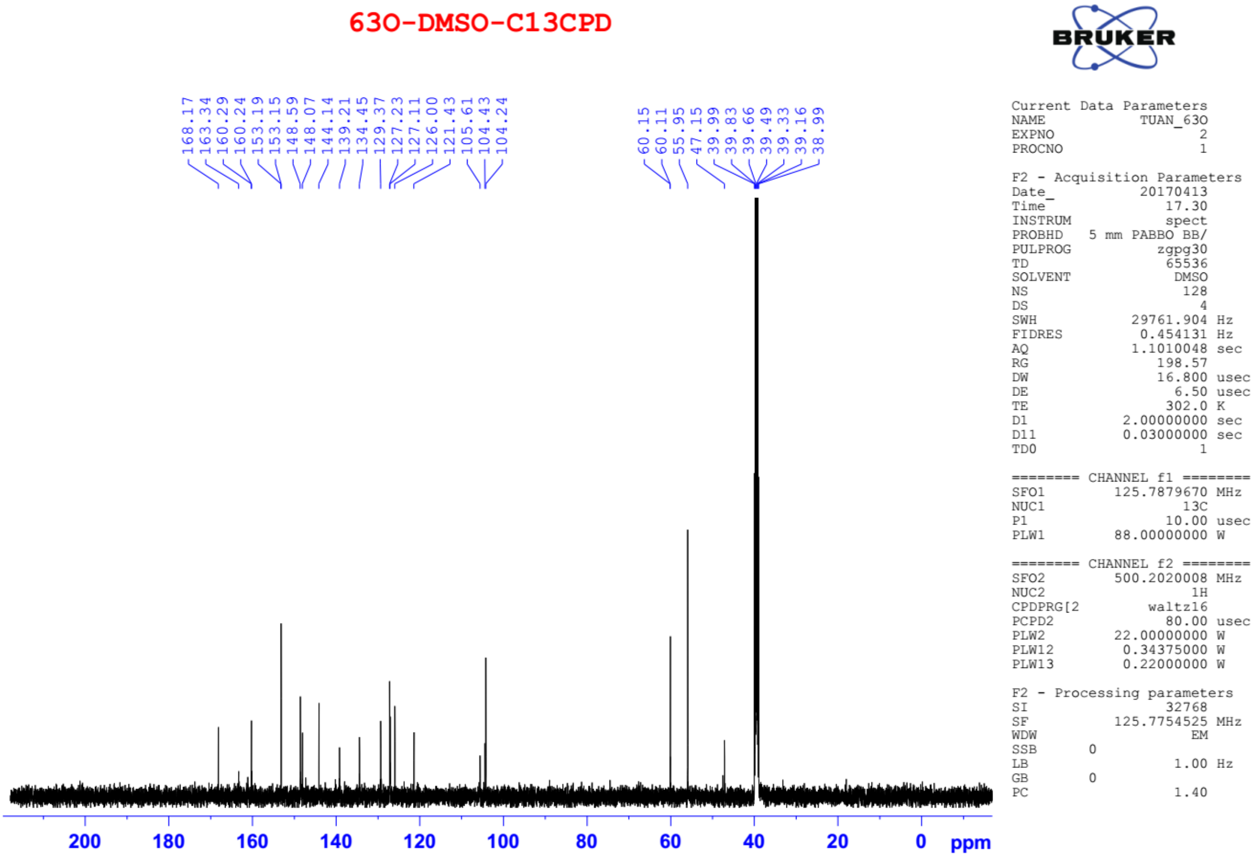

^{1}H NMR of compound 5r

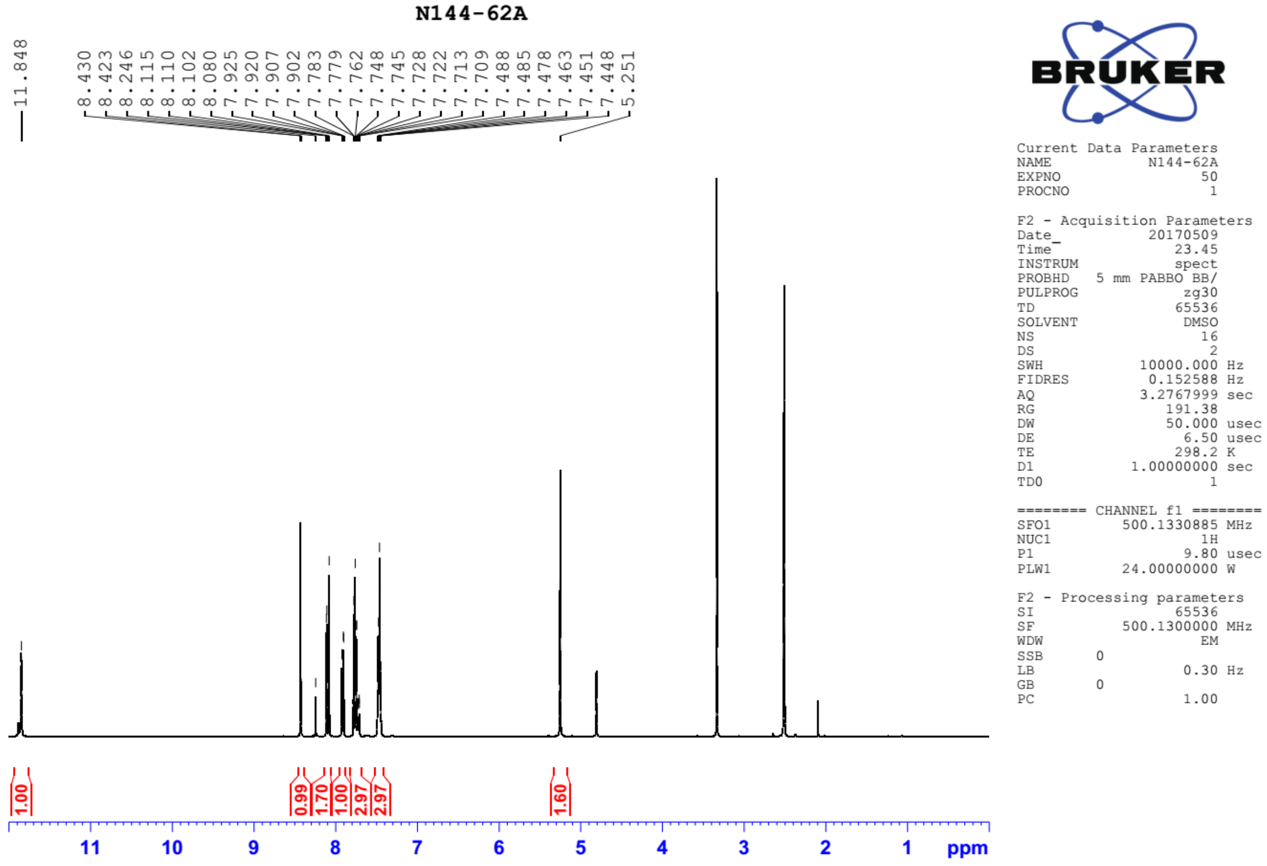

^{13}C NMR of compound 5r

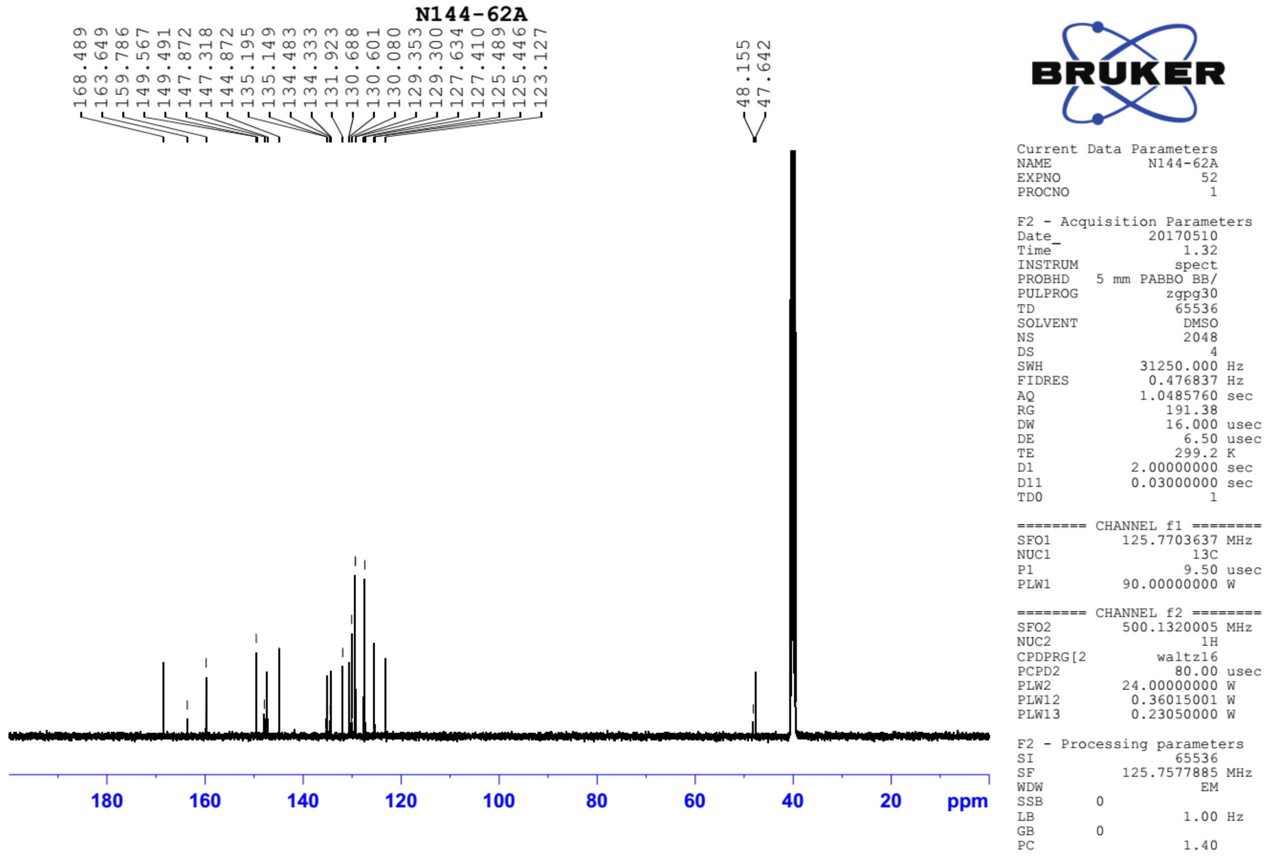

^{1}H NMR of compound 5s

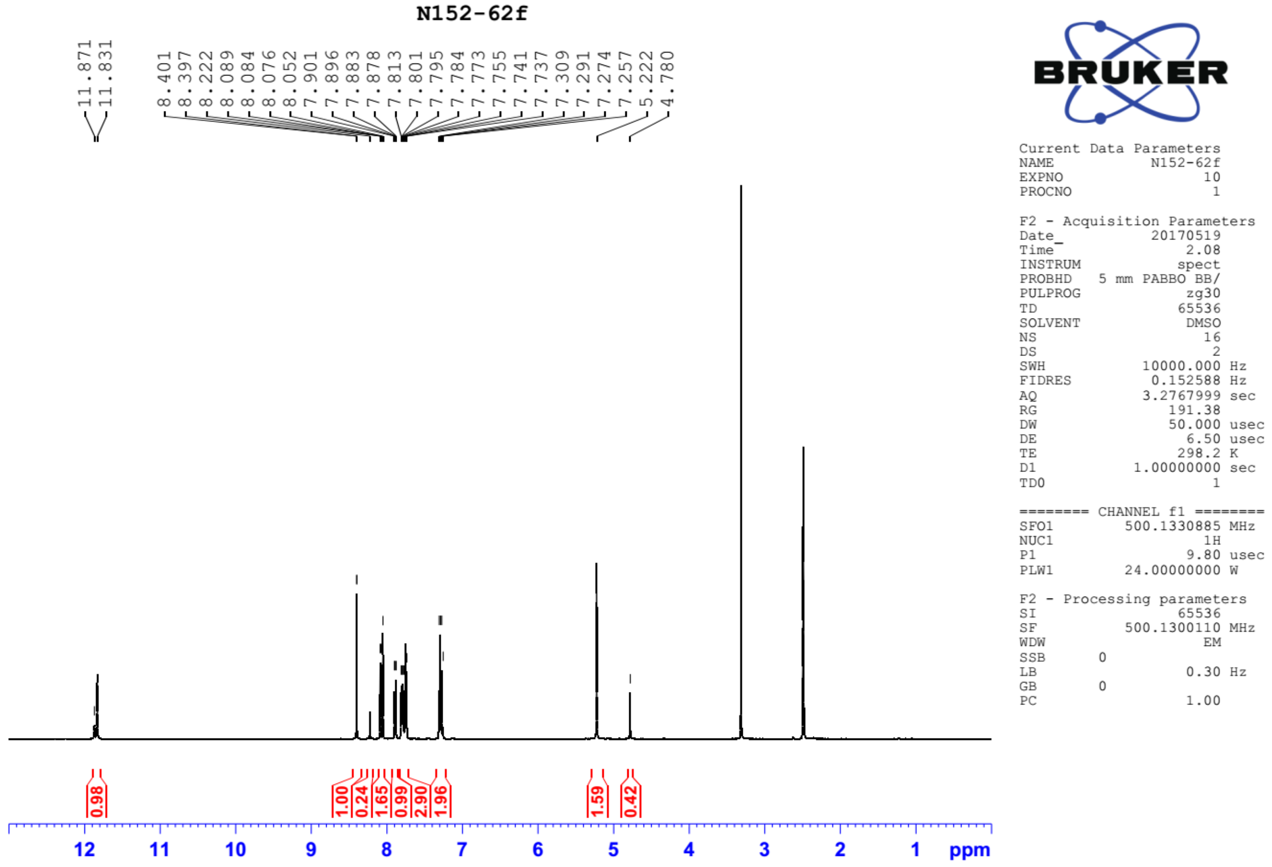

^{13}C NMR of compound 5s

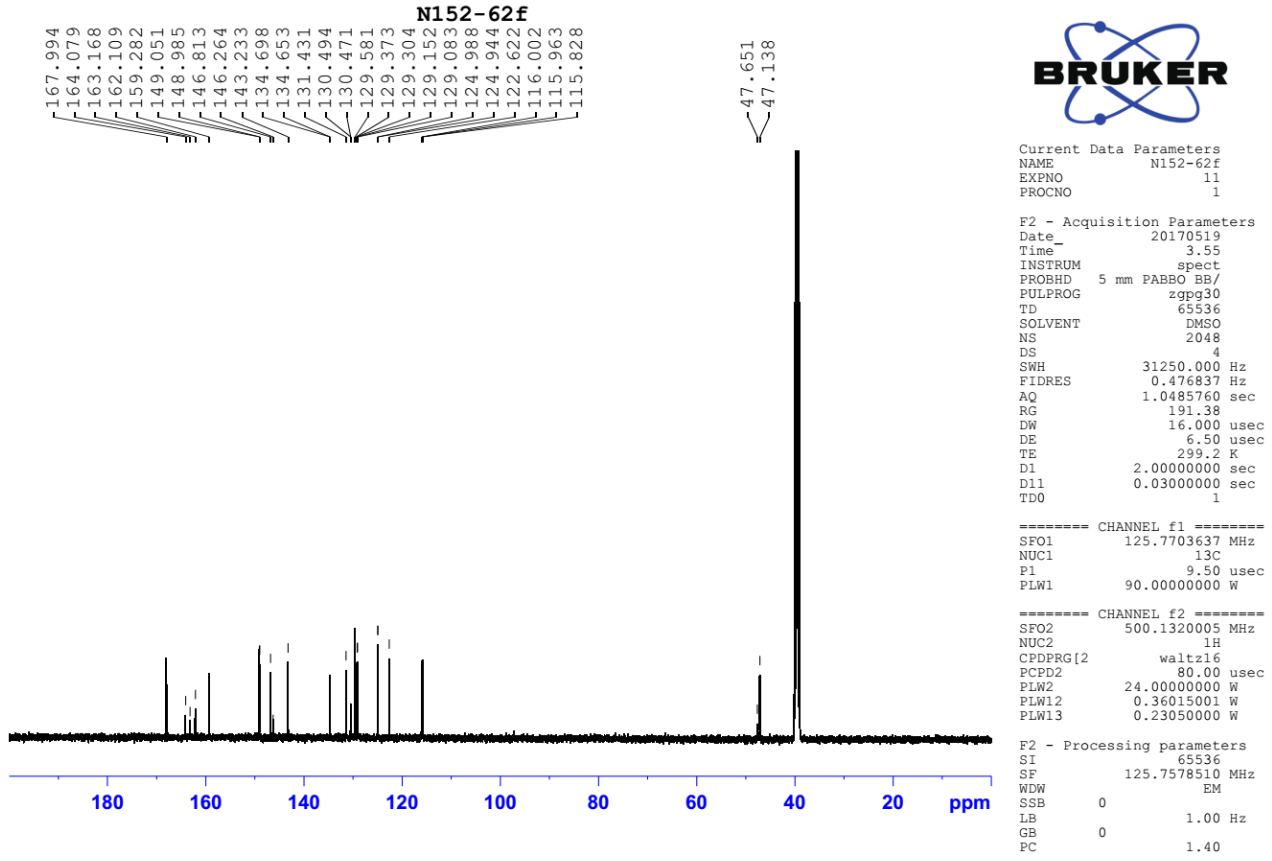

^{1}H NMR of compound 5t

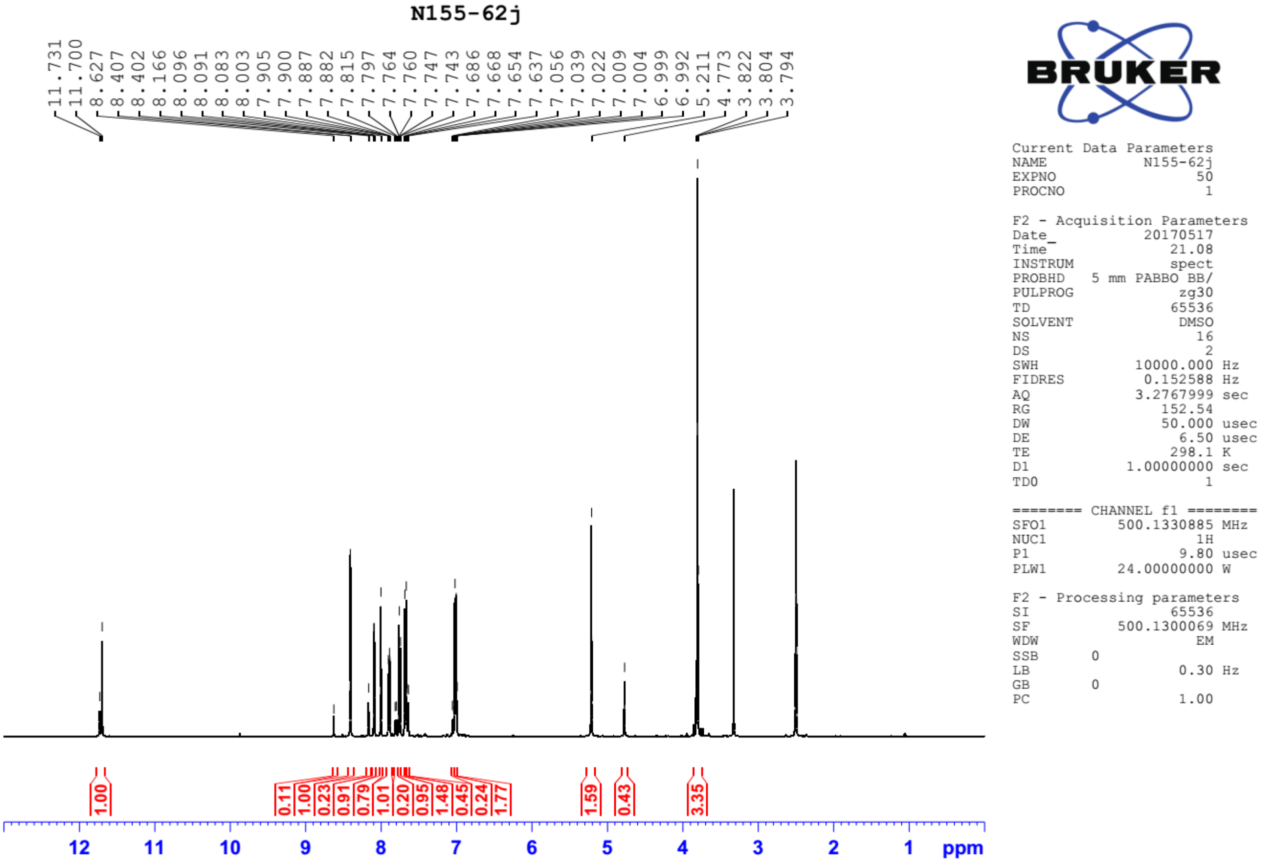

^{13}C NMR of compound 5t

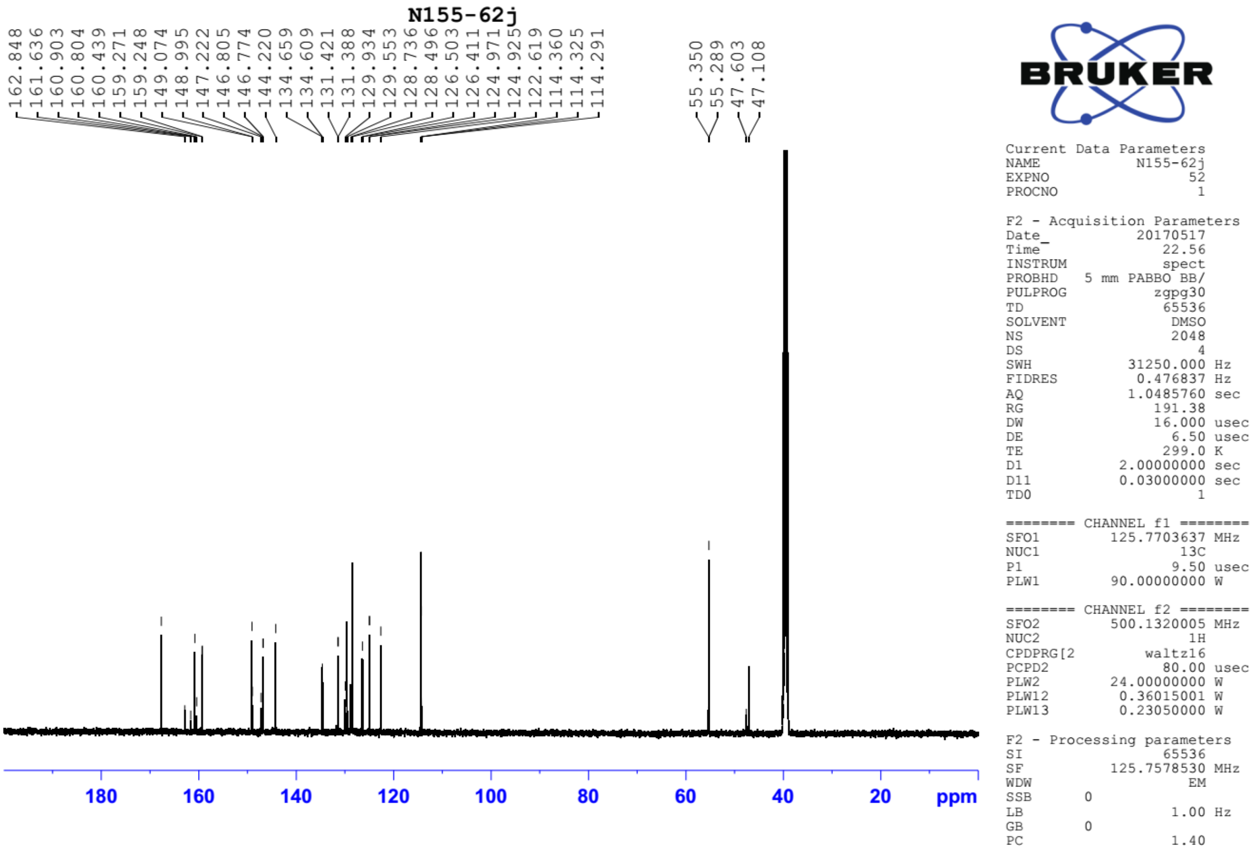

^{1}H NMR of compound 5u

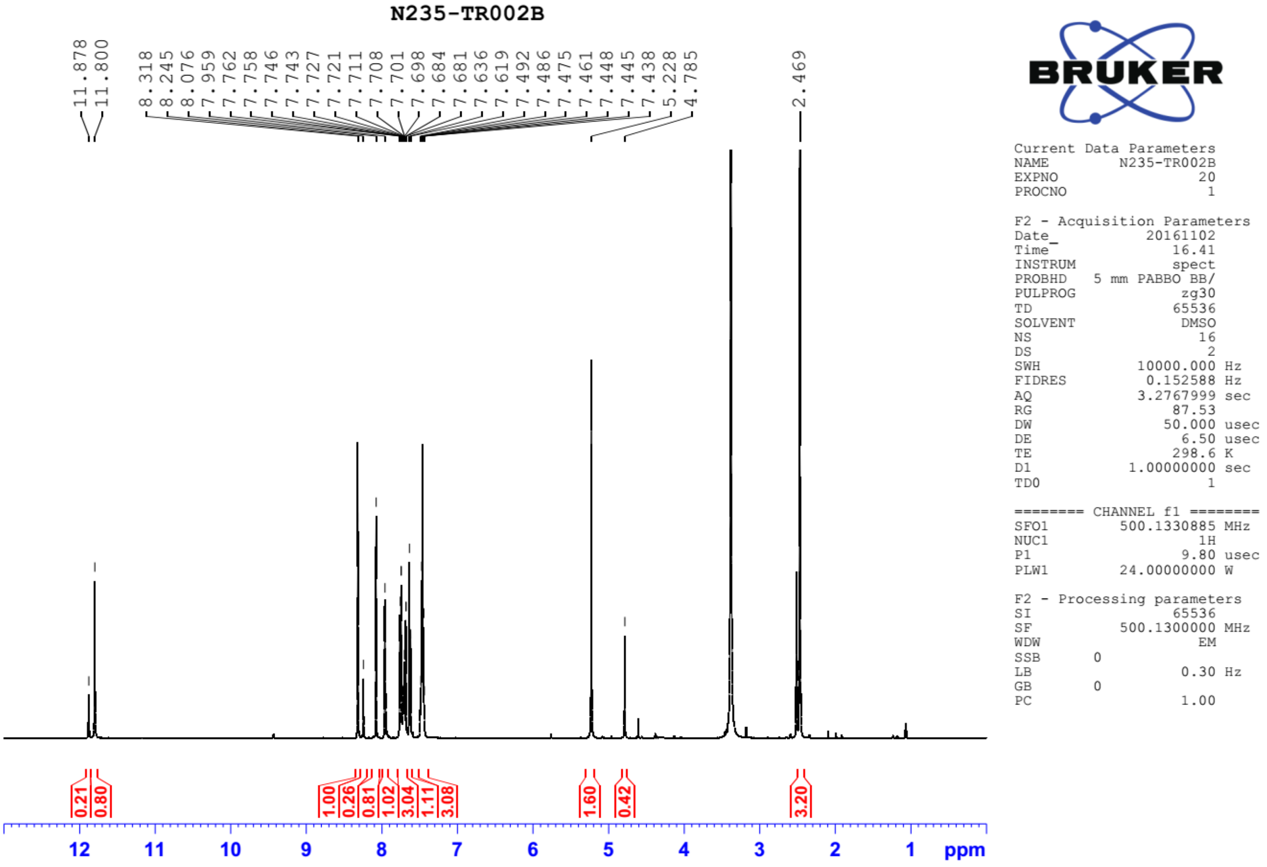

^{13}C NMR of compound 5u

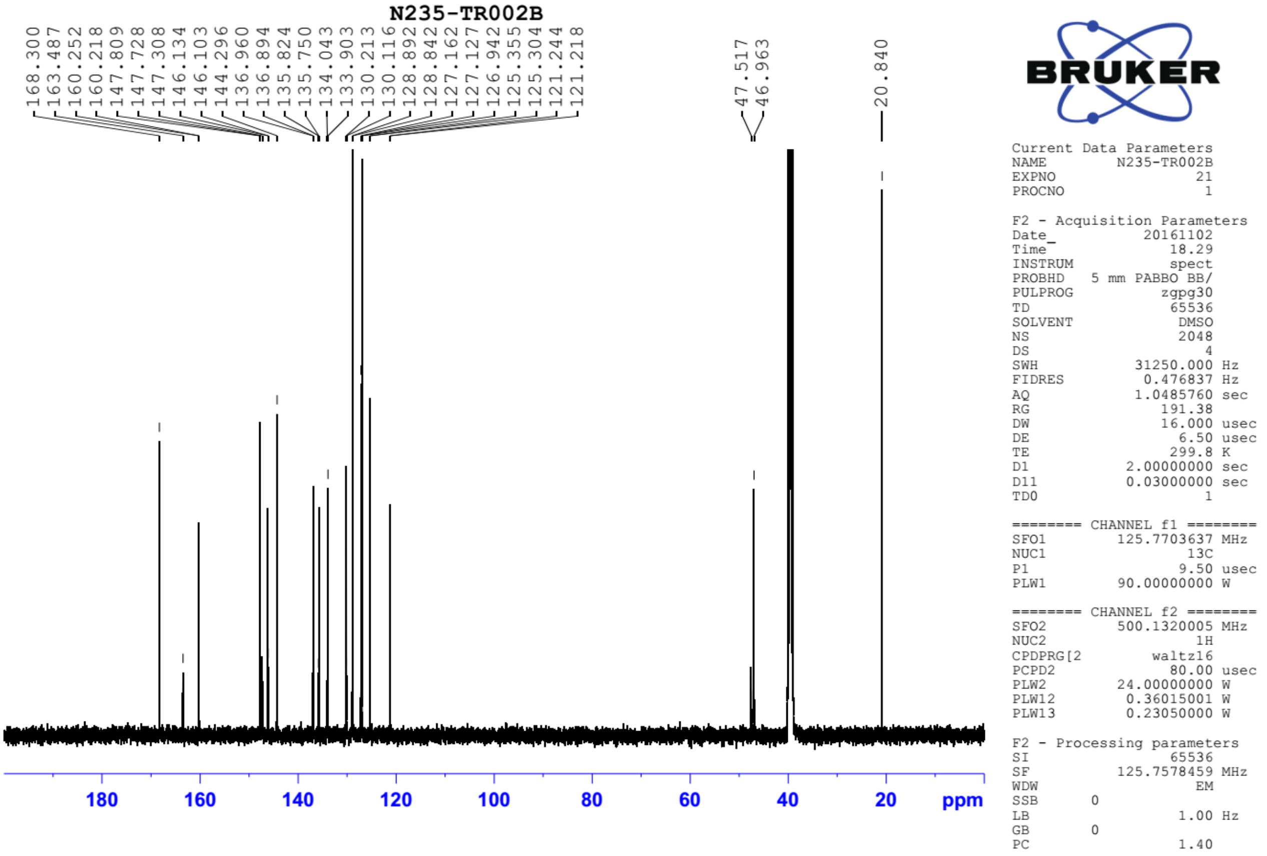

^1H NMR of compound 5v

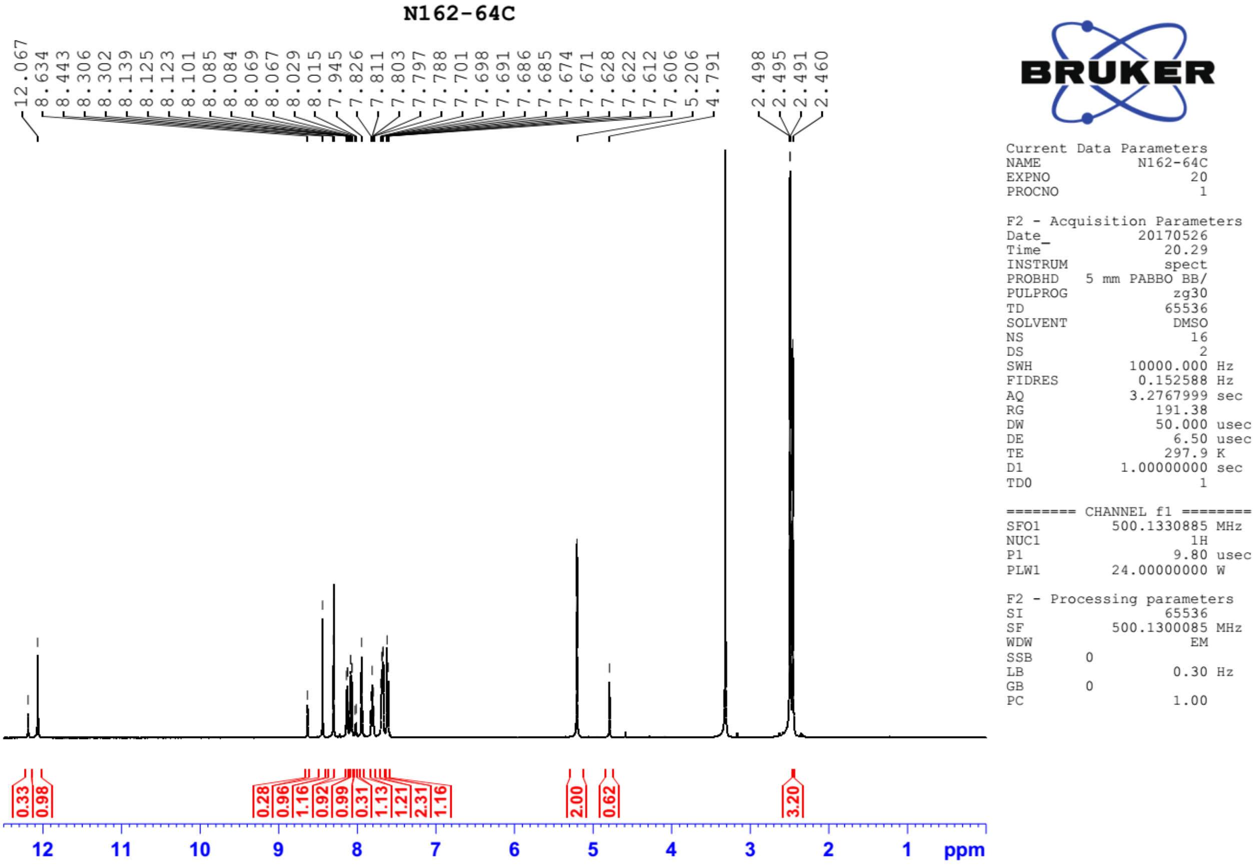

^{13}C NMR of compound 5v

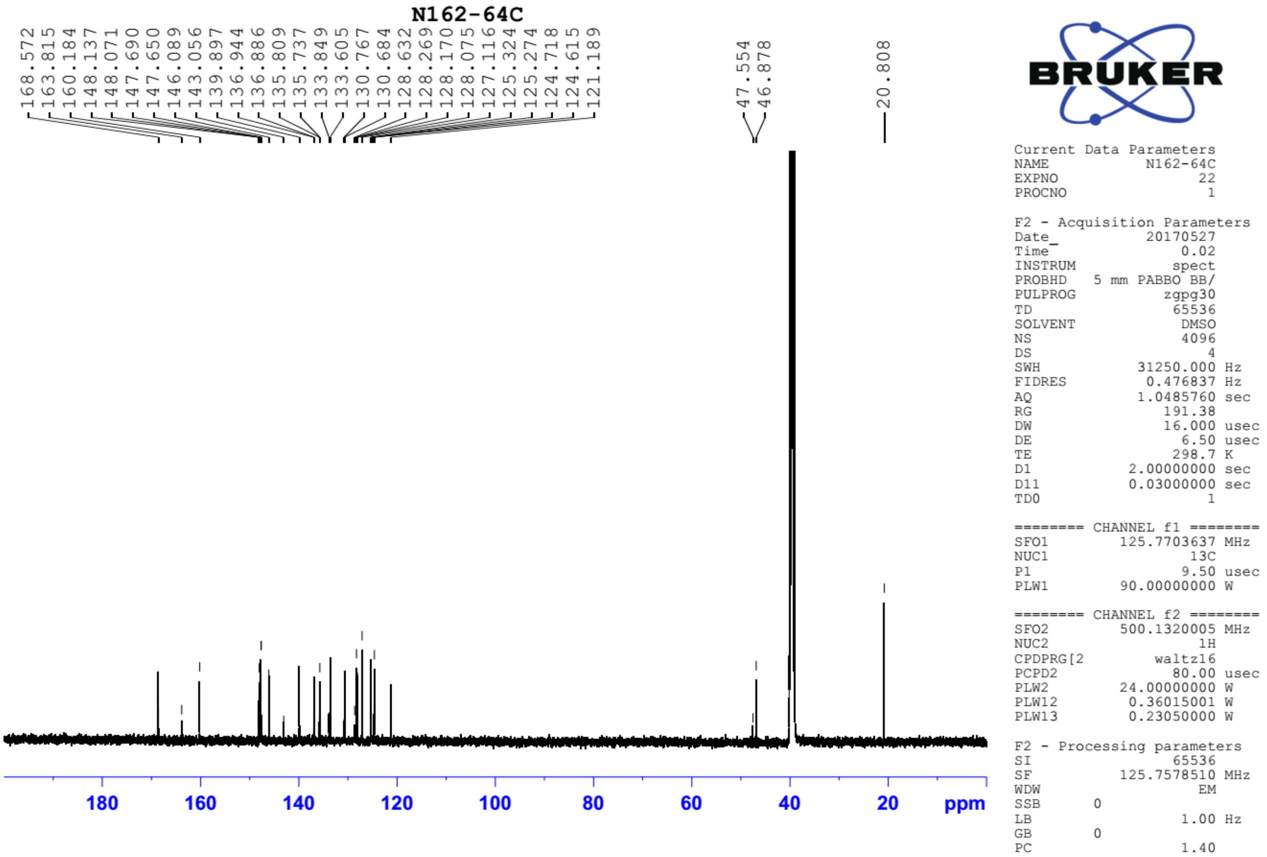

^{1}H NMR of compound 5w

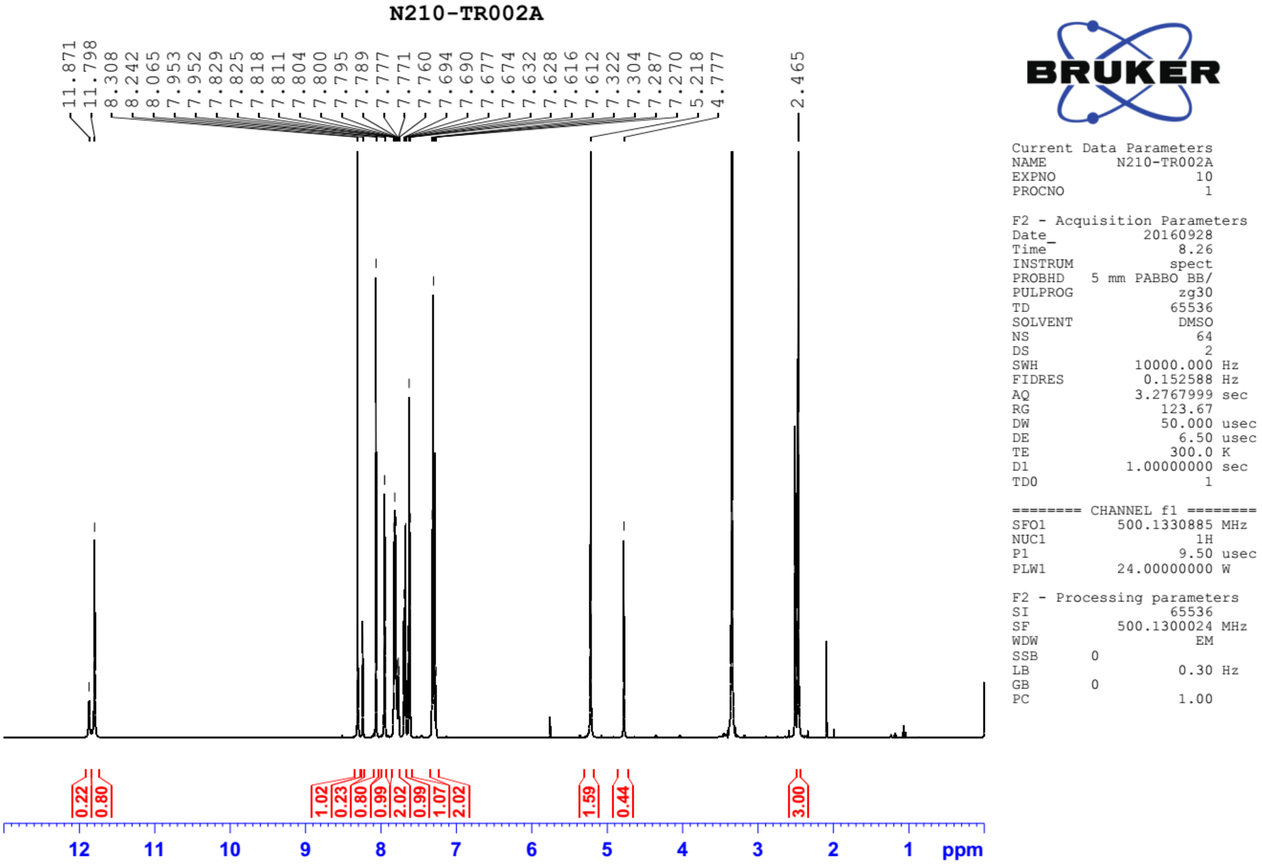

^{13}C NMR of compound 5w

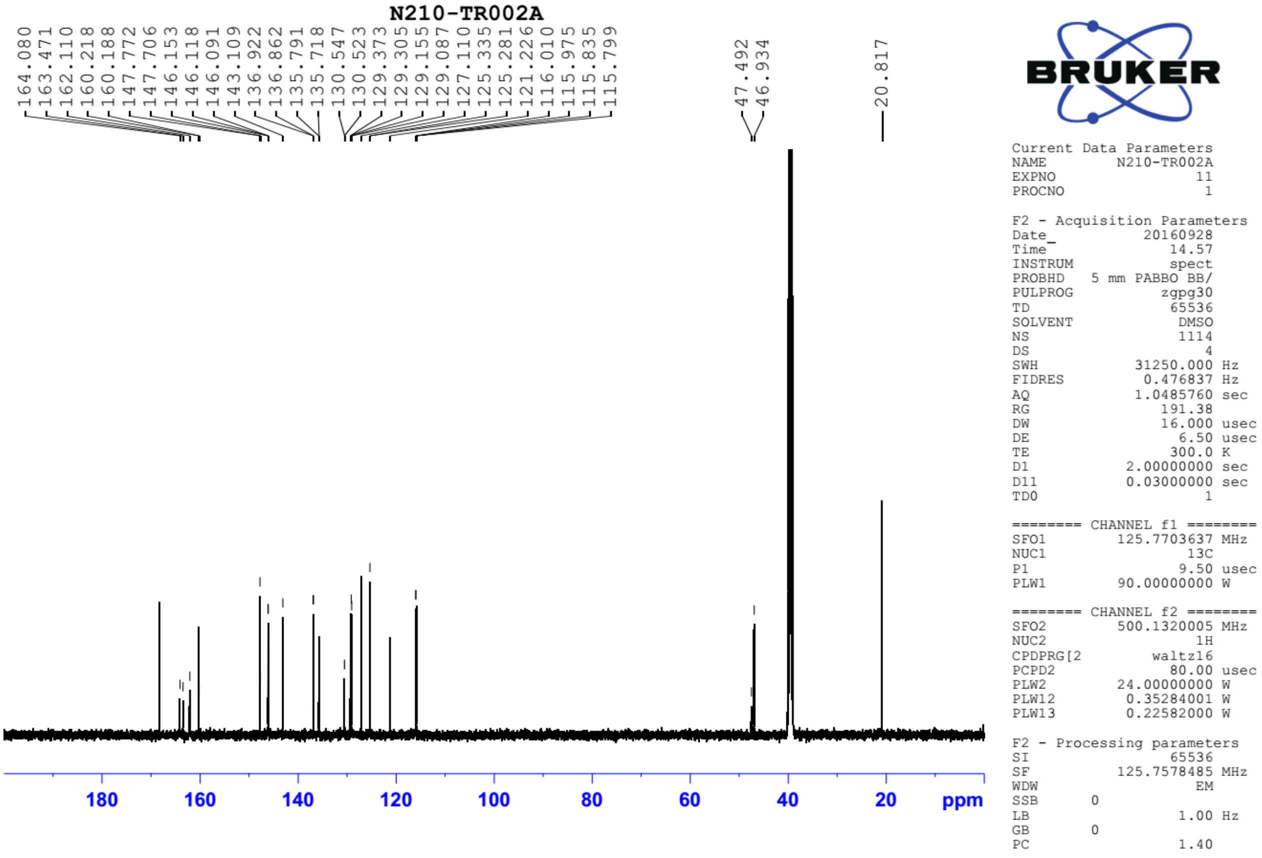

^{1}H NMR of compound 5y

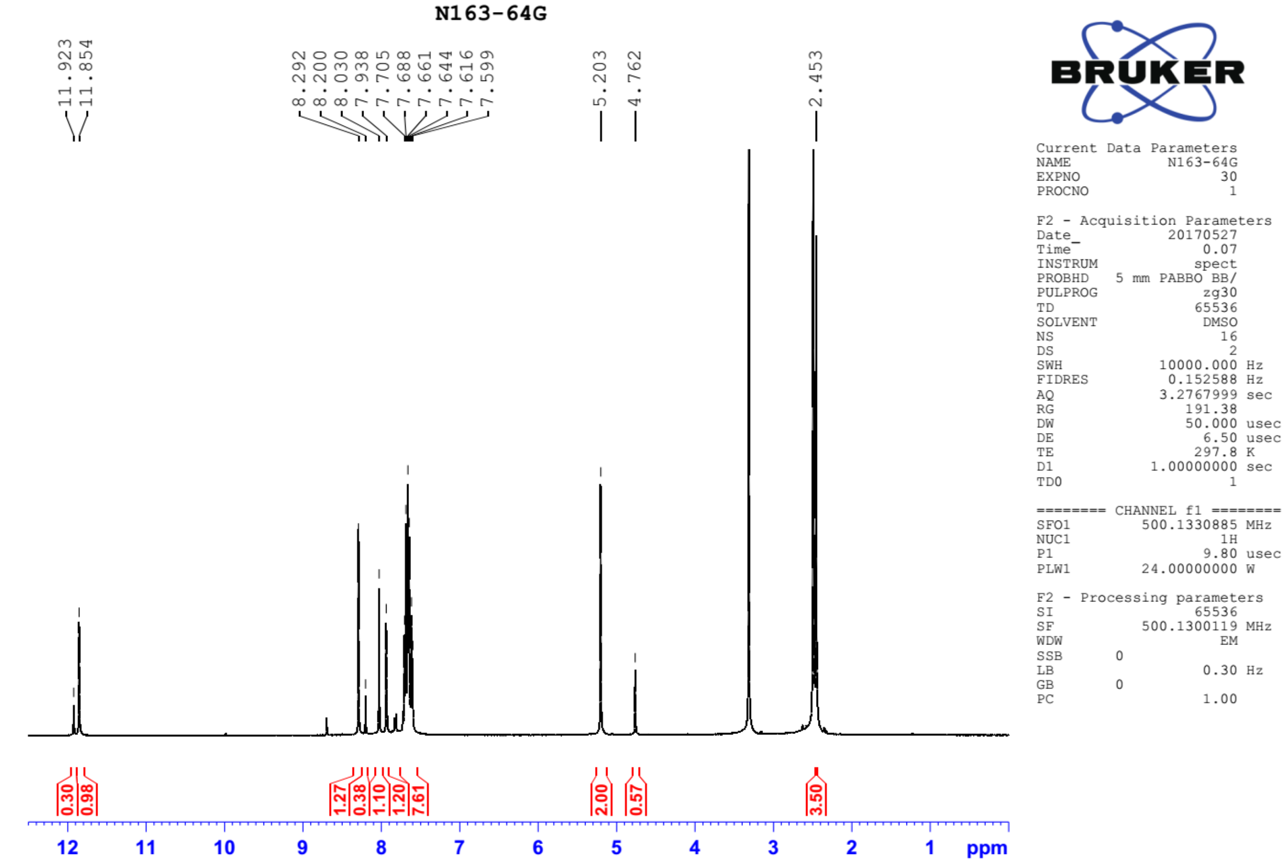

^{13}C NMR of compound 5y

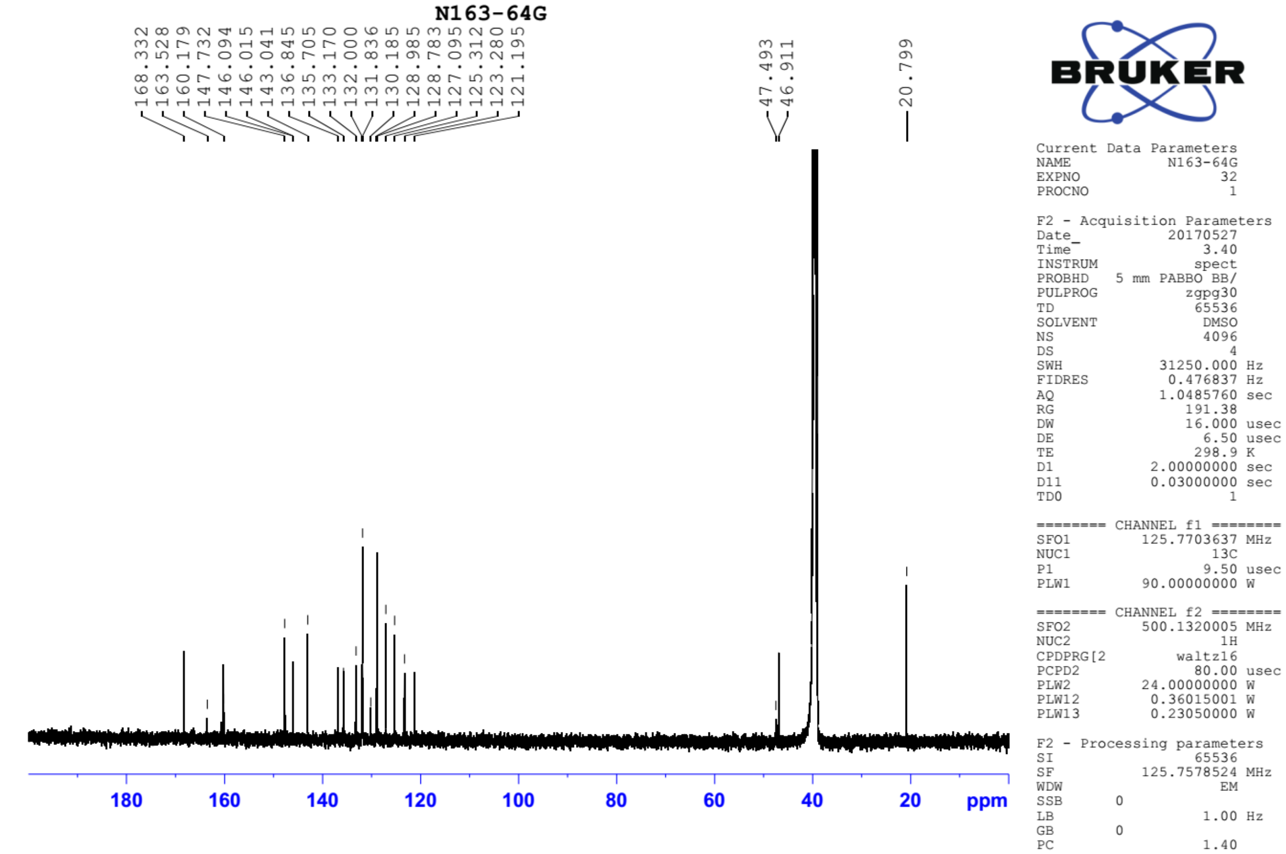

^{1}H NMR of compound 5x

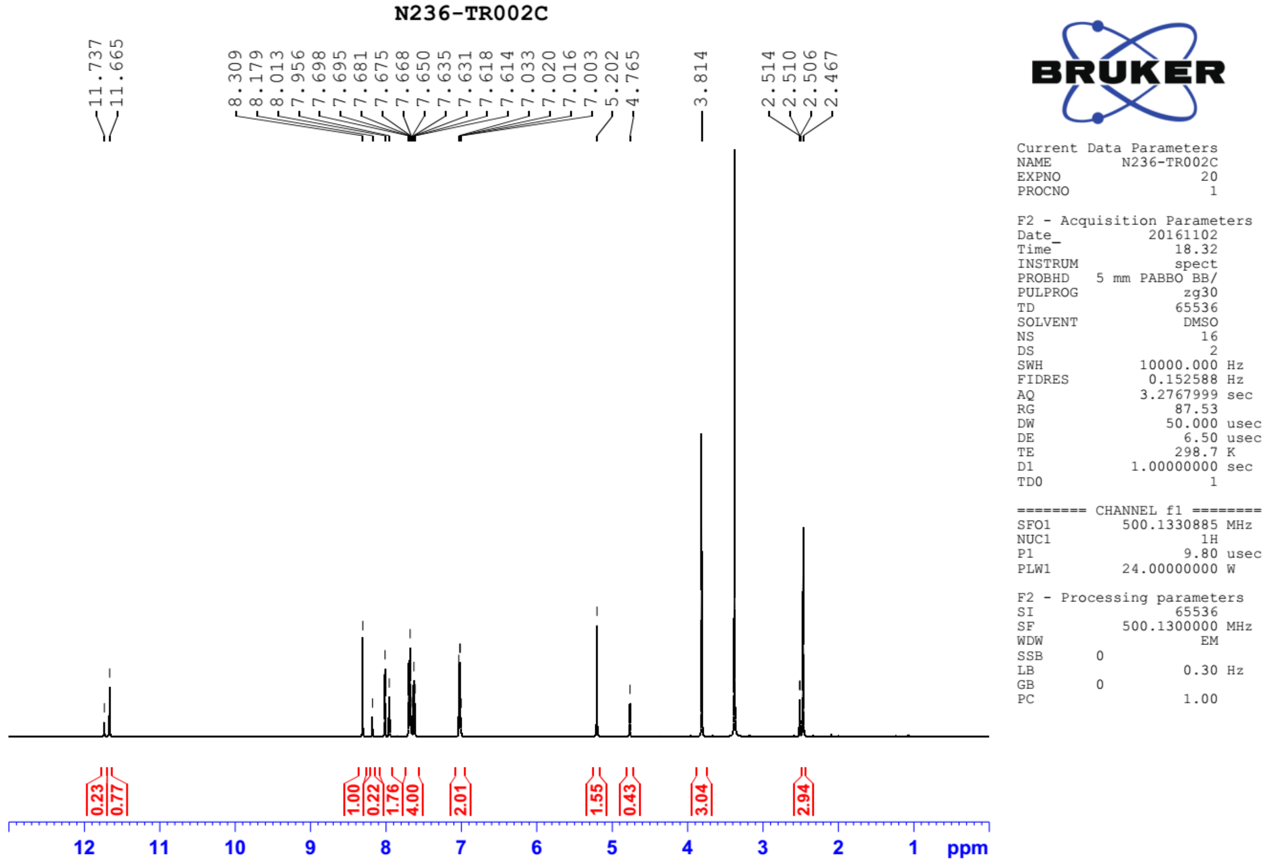

^{13}C NMR of compound 5x

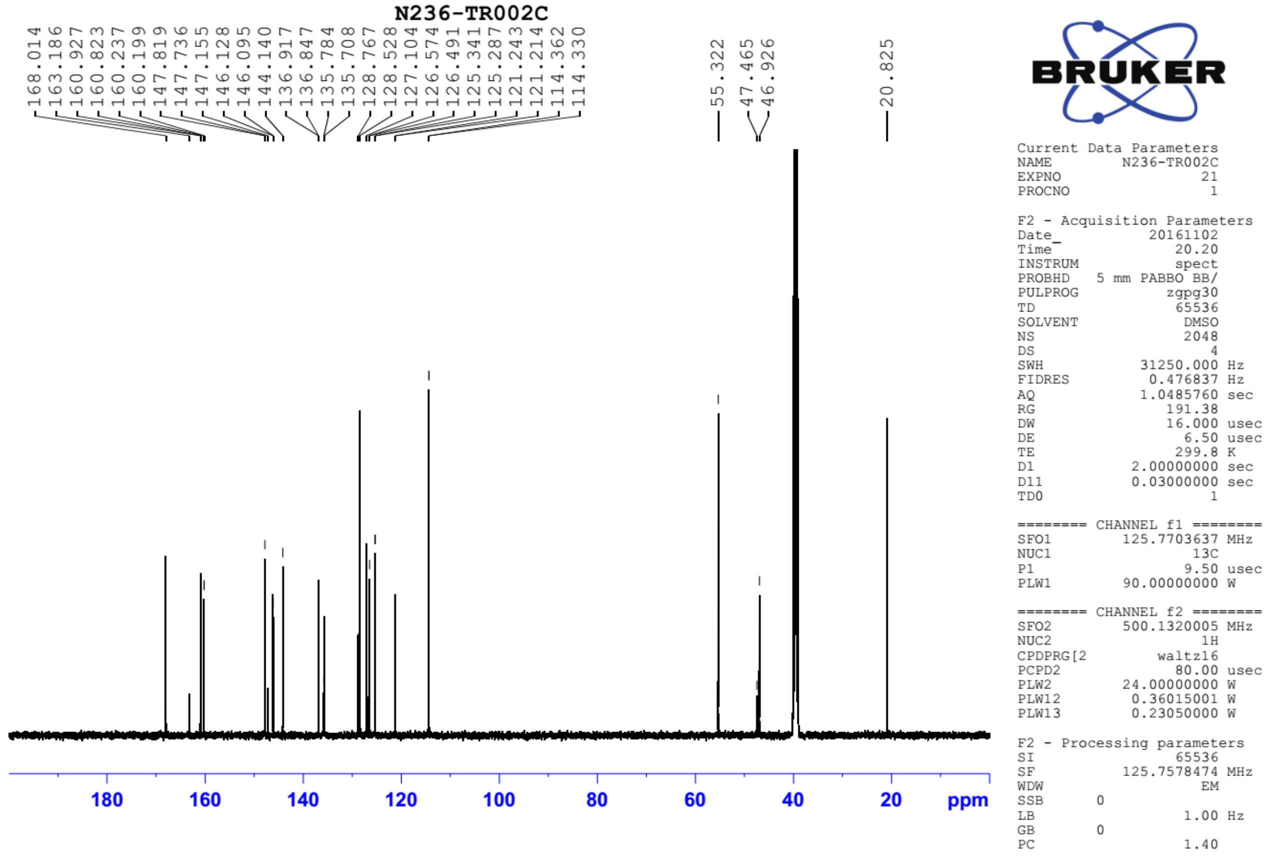

^{1}H NMR of compound 5z

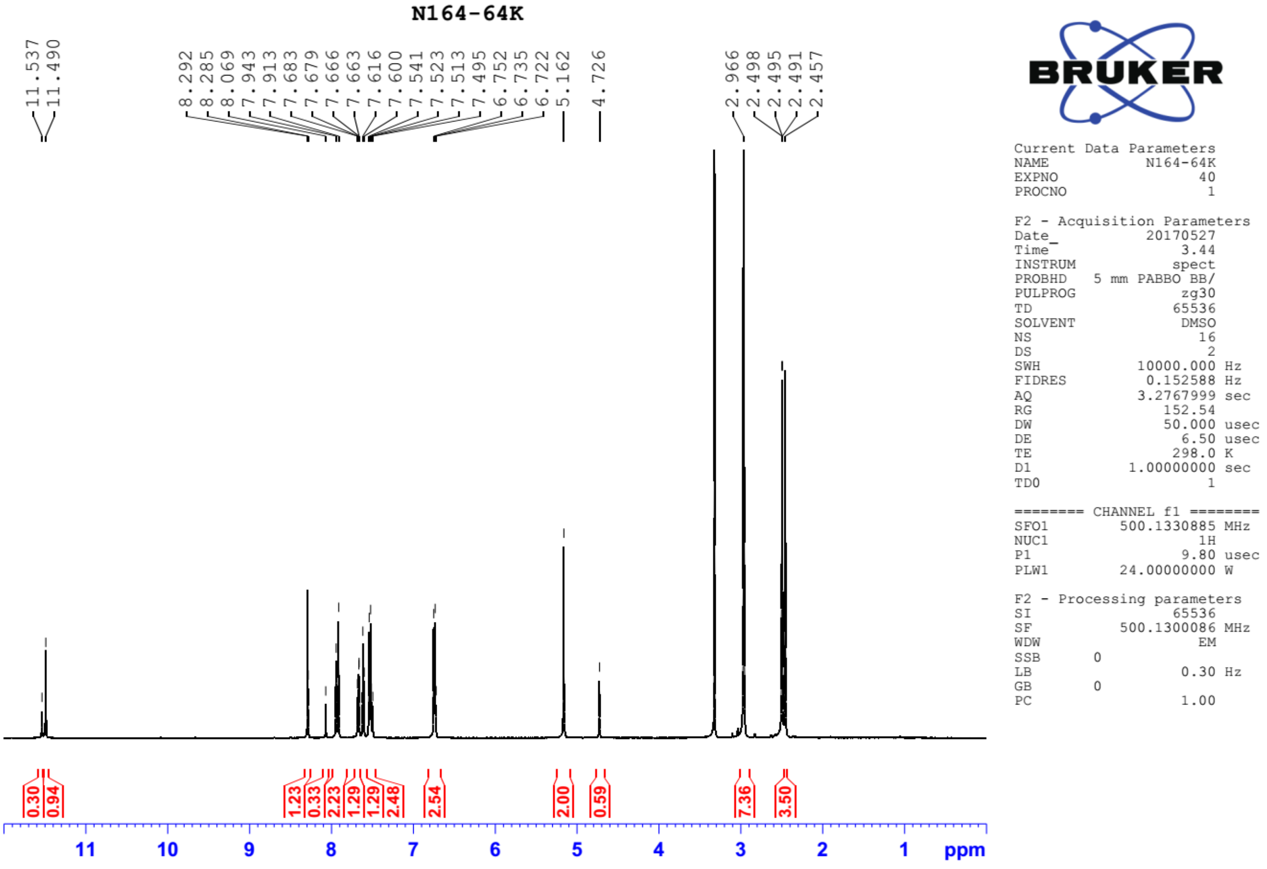

^{13}C NMR of compound 5z

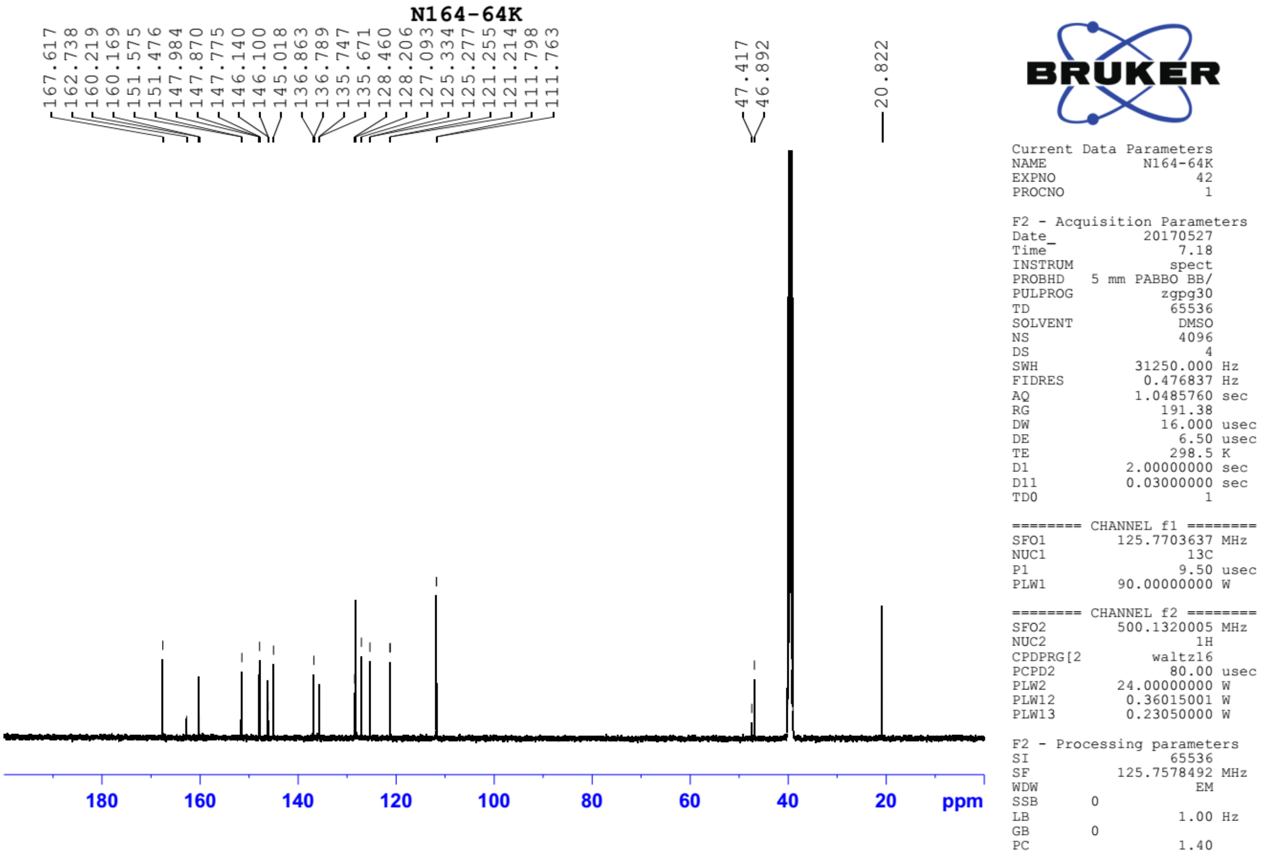

^{1}H NMR of compound 6a

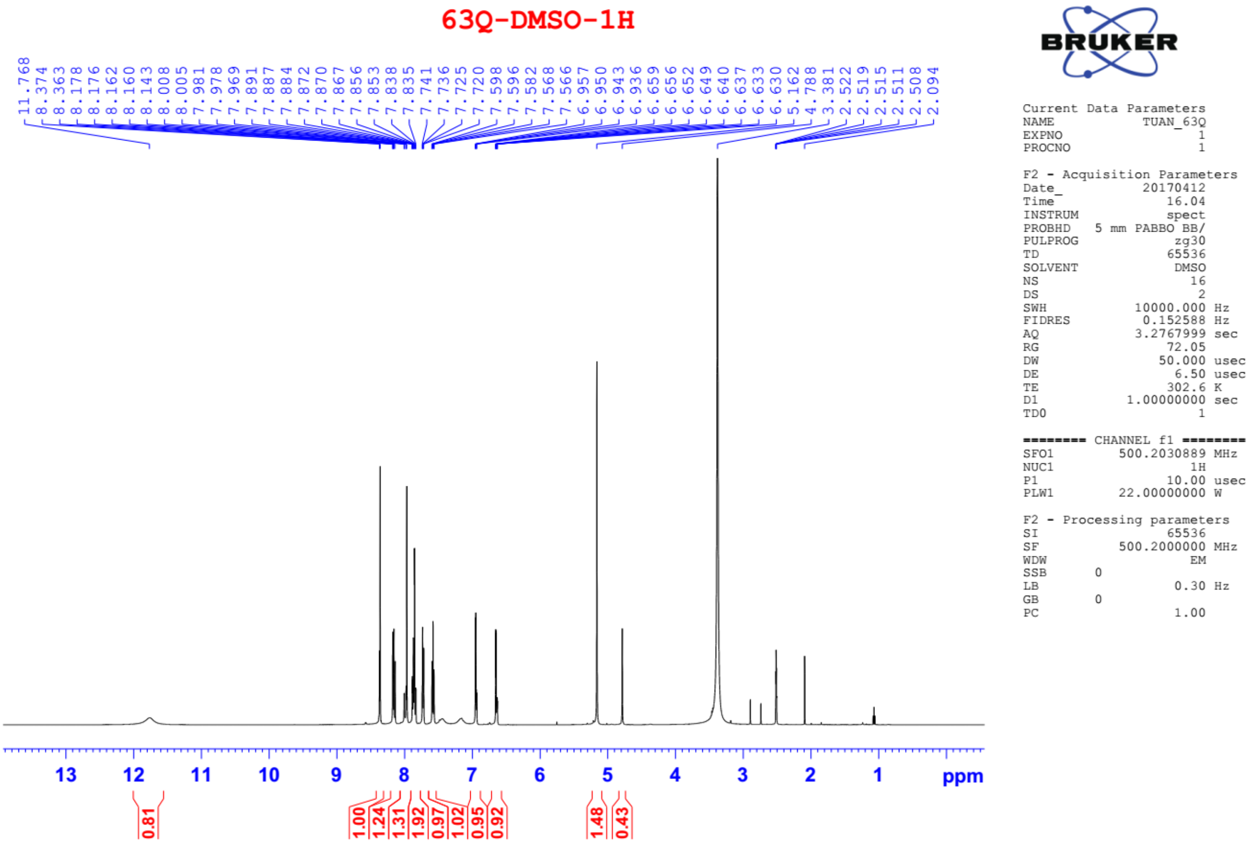

^{13}C NMR of compound 6a

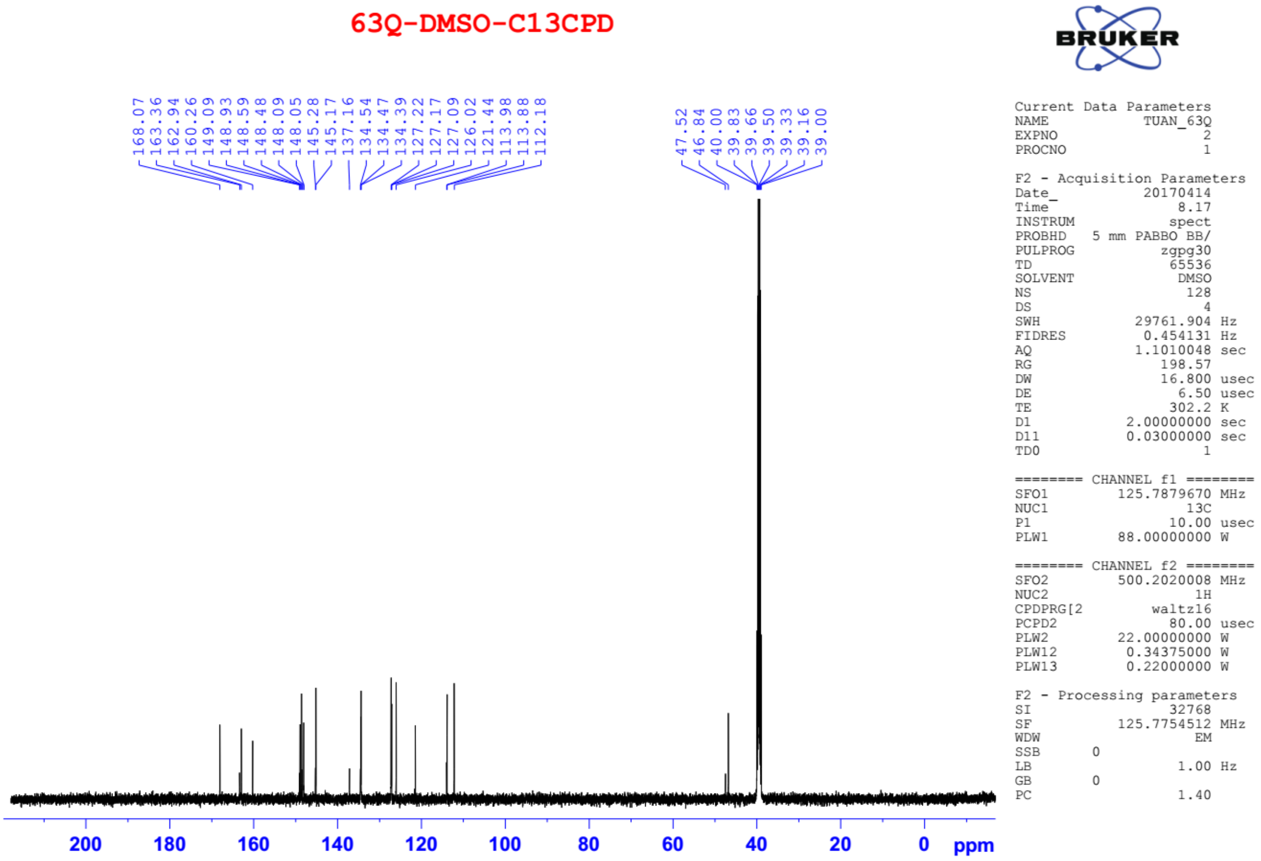

^{1}H NMR of compound 6b

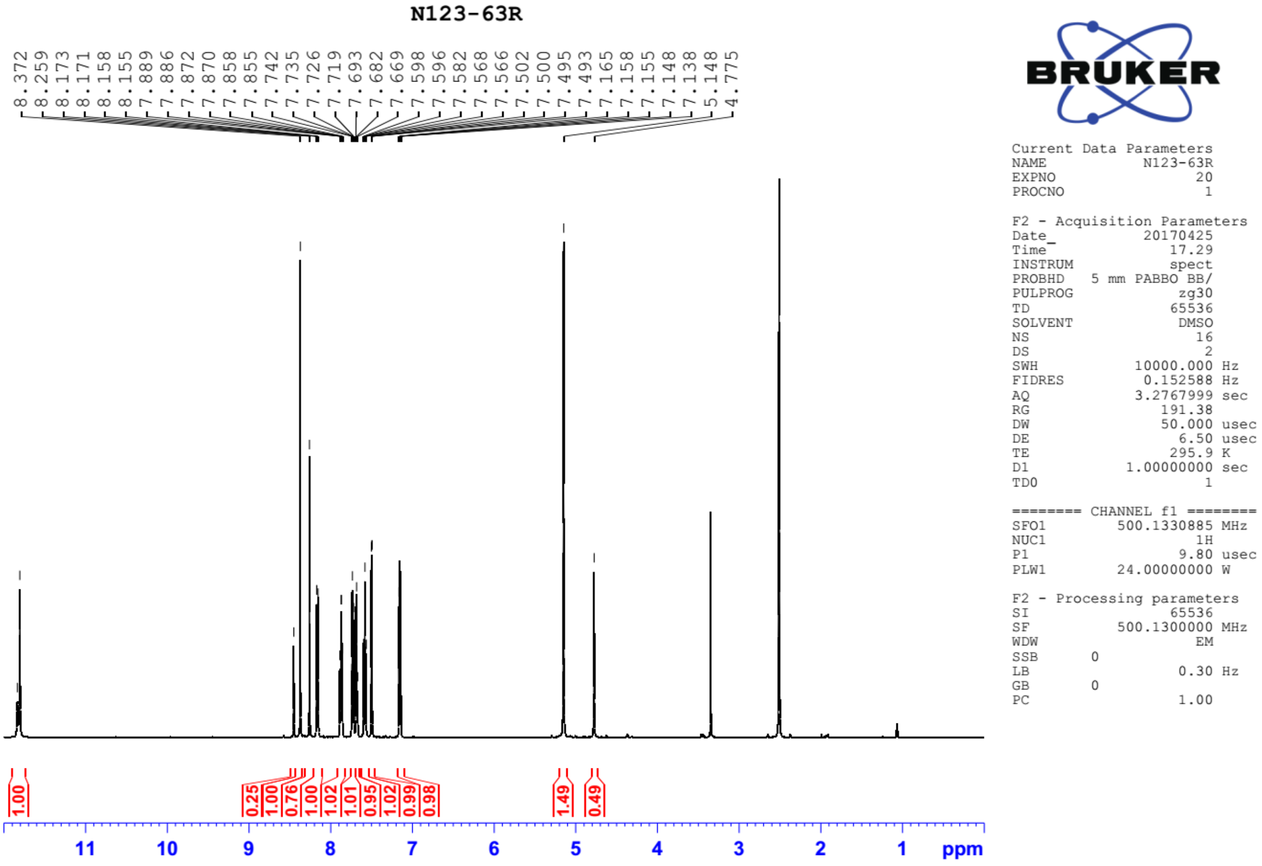

^{13}C NMR of compound 6b

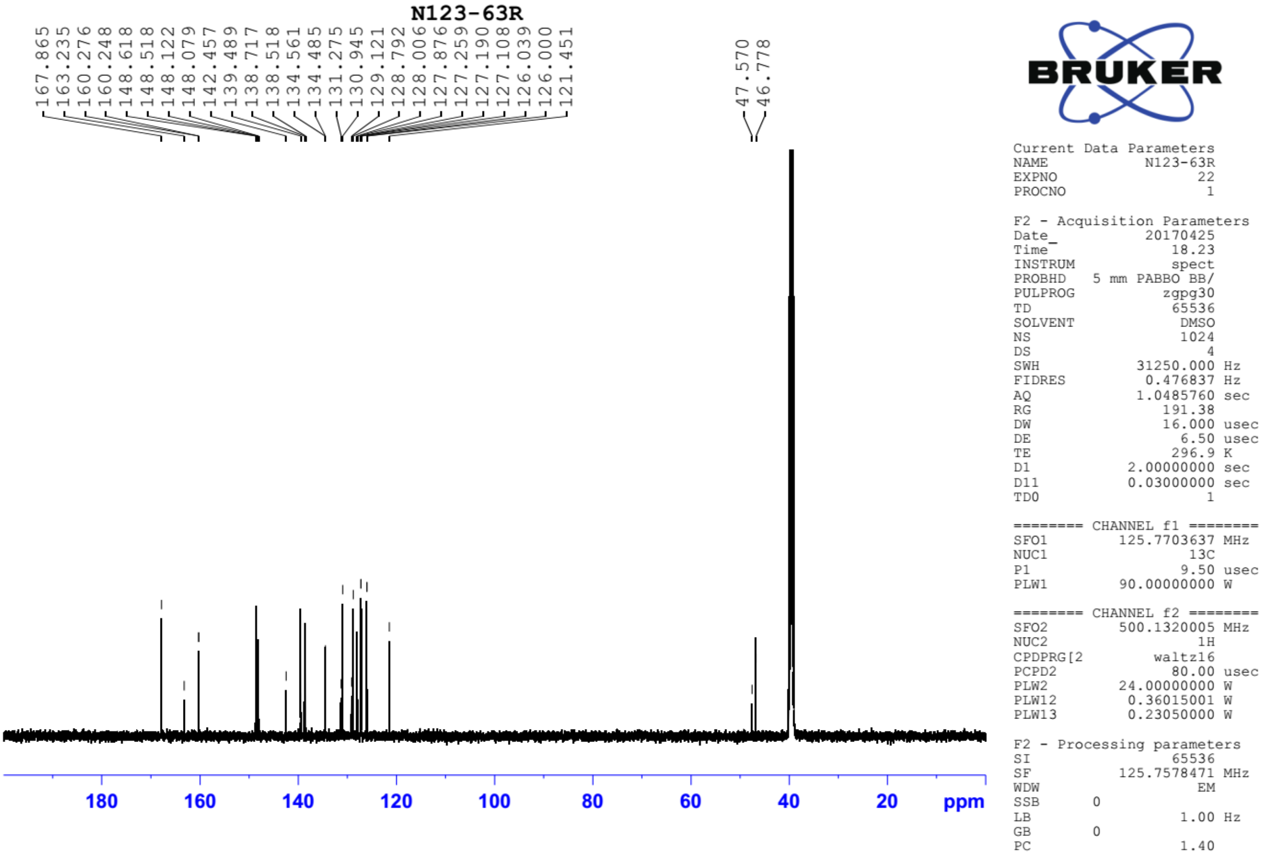

^{1}H NMR of compound 6c

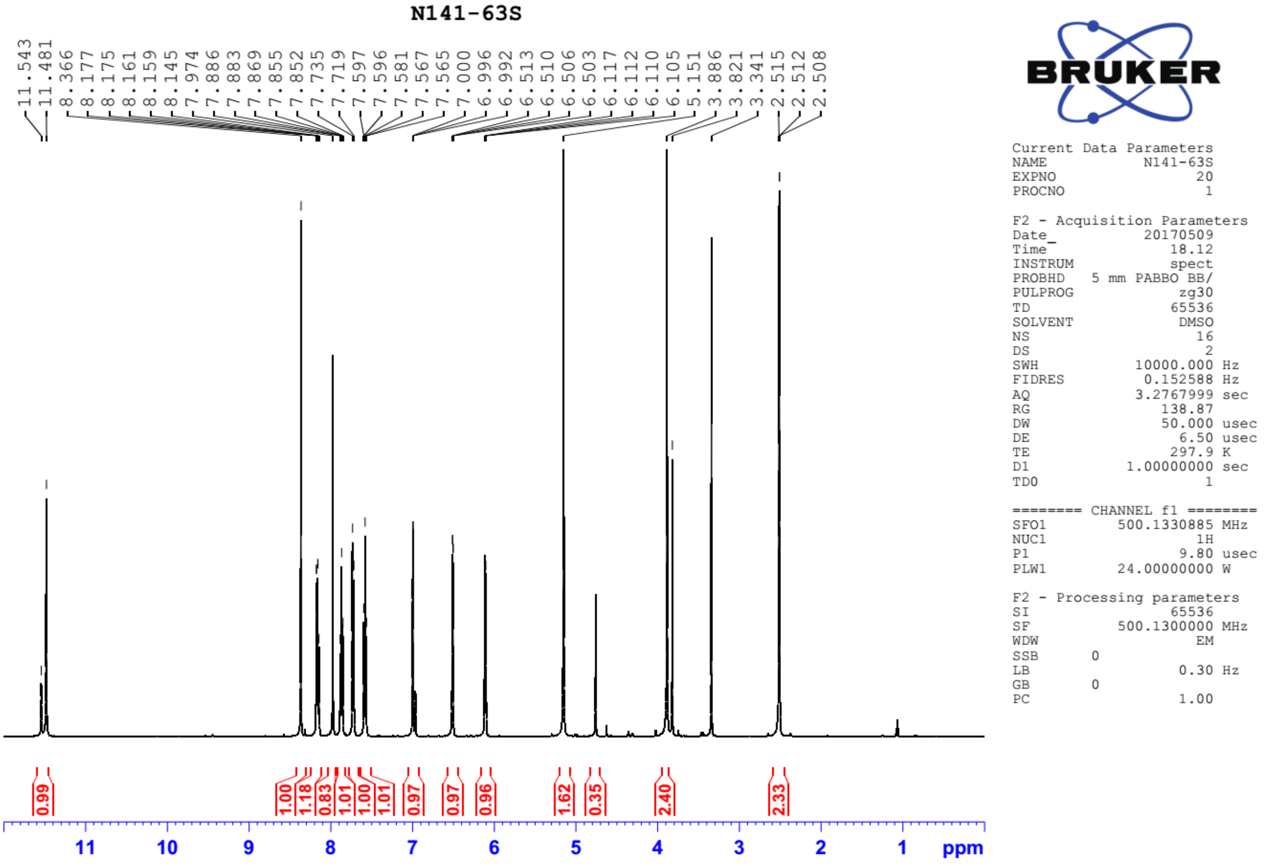

^{13}C NMR of compound 6c

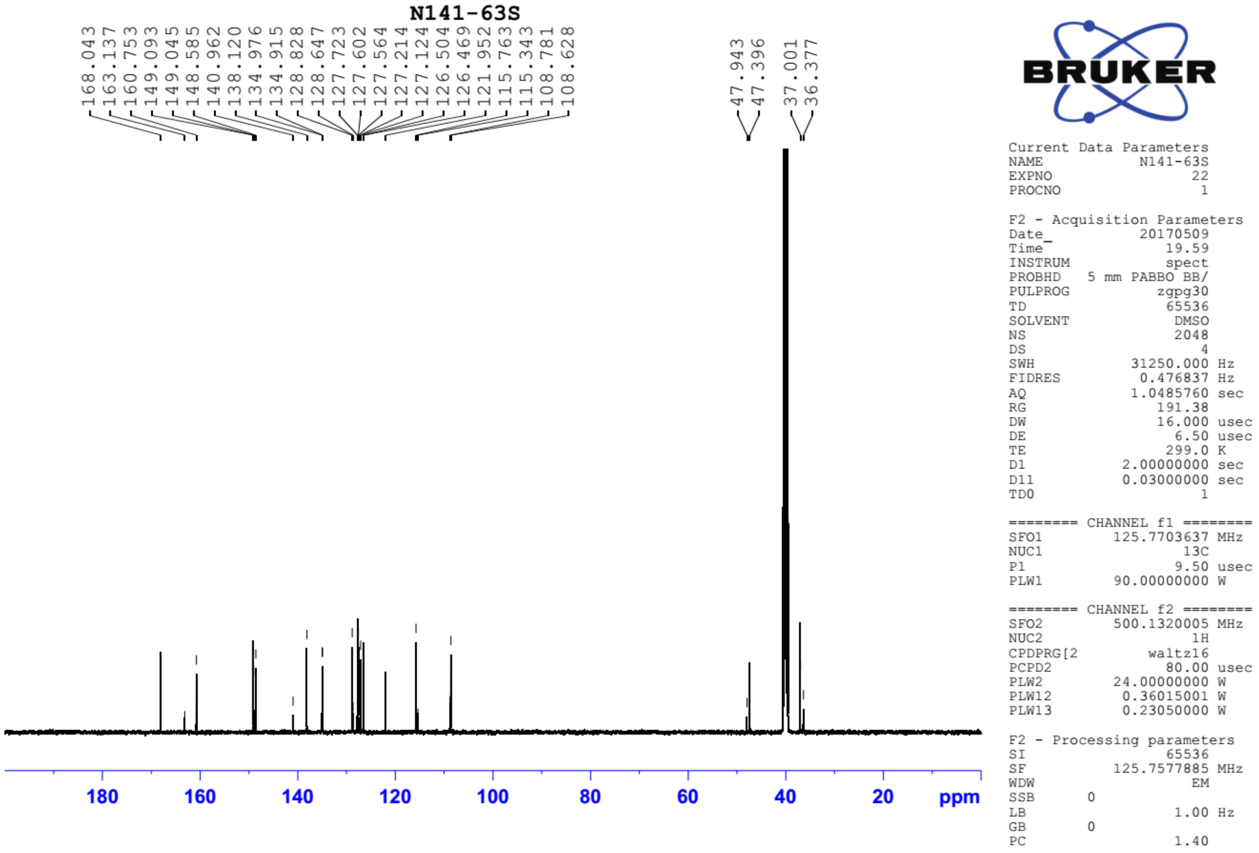

^{1}H NMR of compound 6d

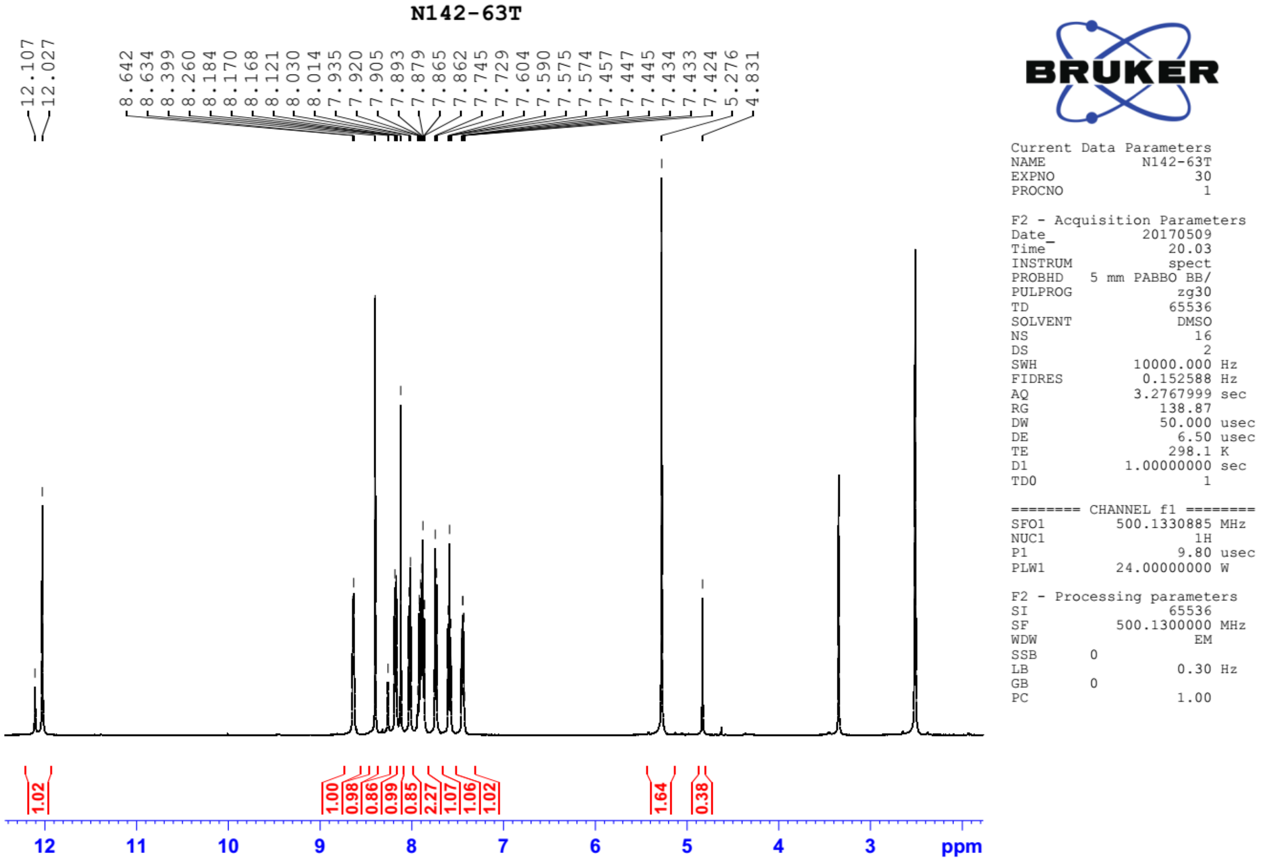

^{13}C NMR of compound 6d

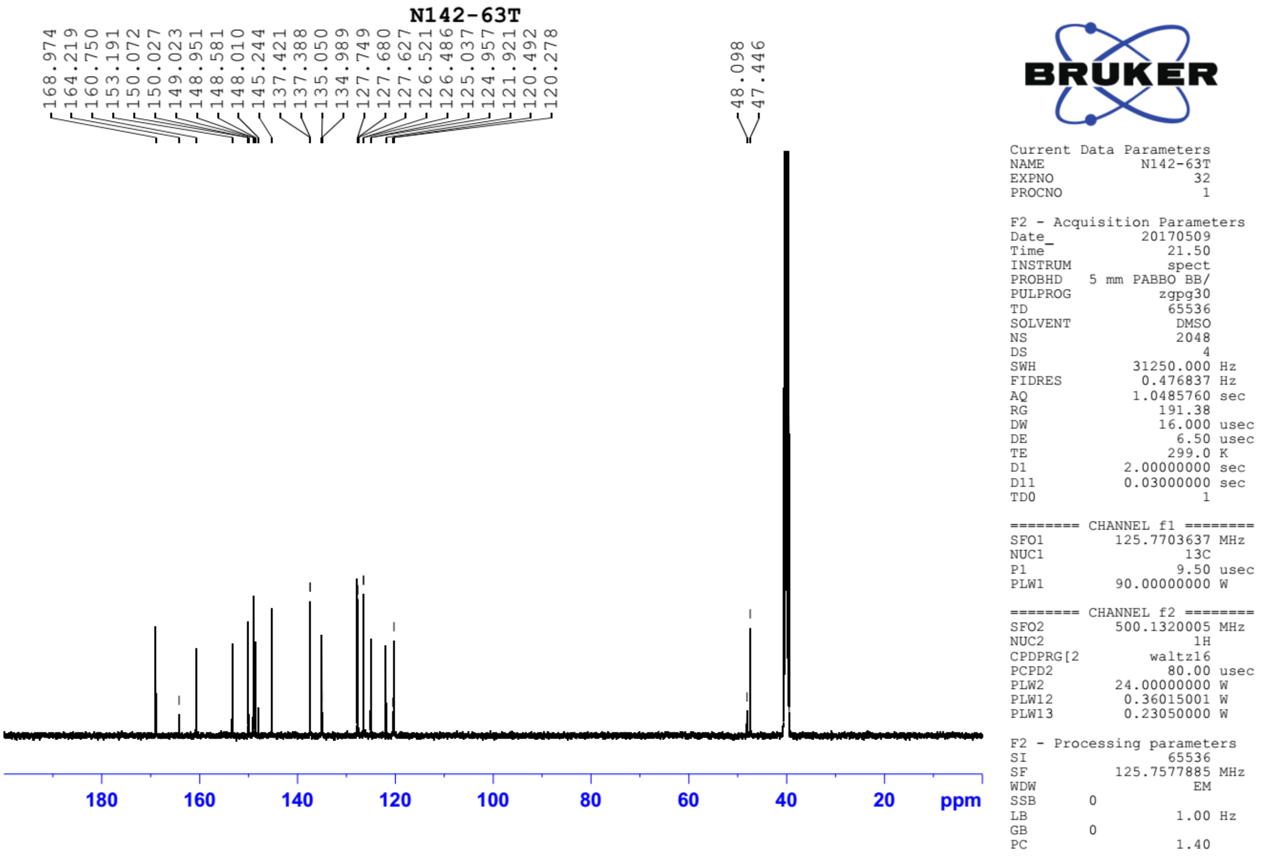

^{1}H NMR of compound 6e

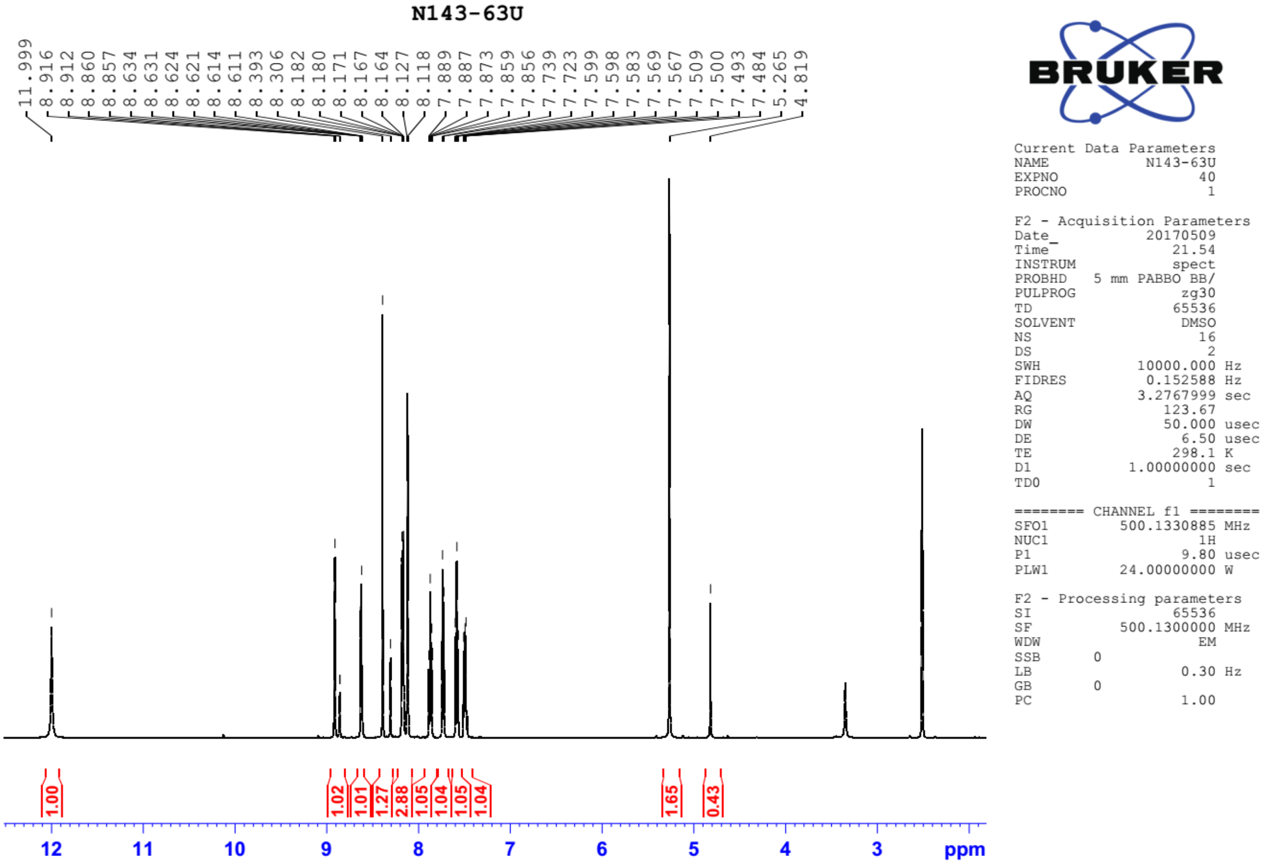

^{13}C NMR of compound 6e

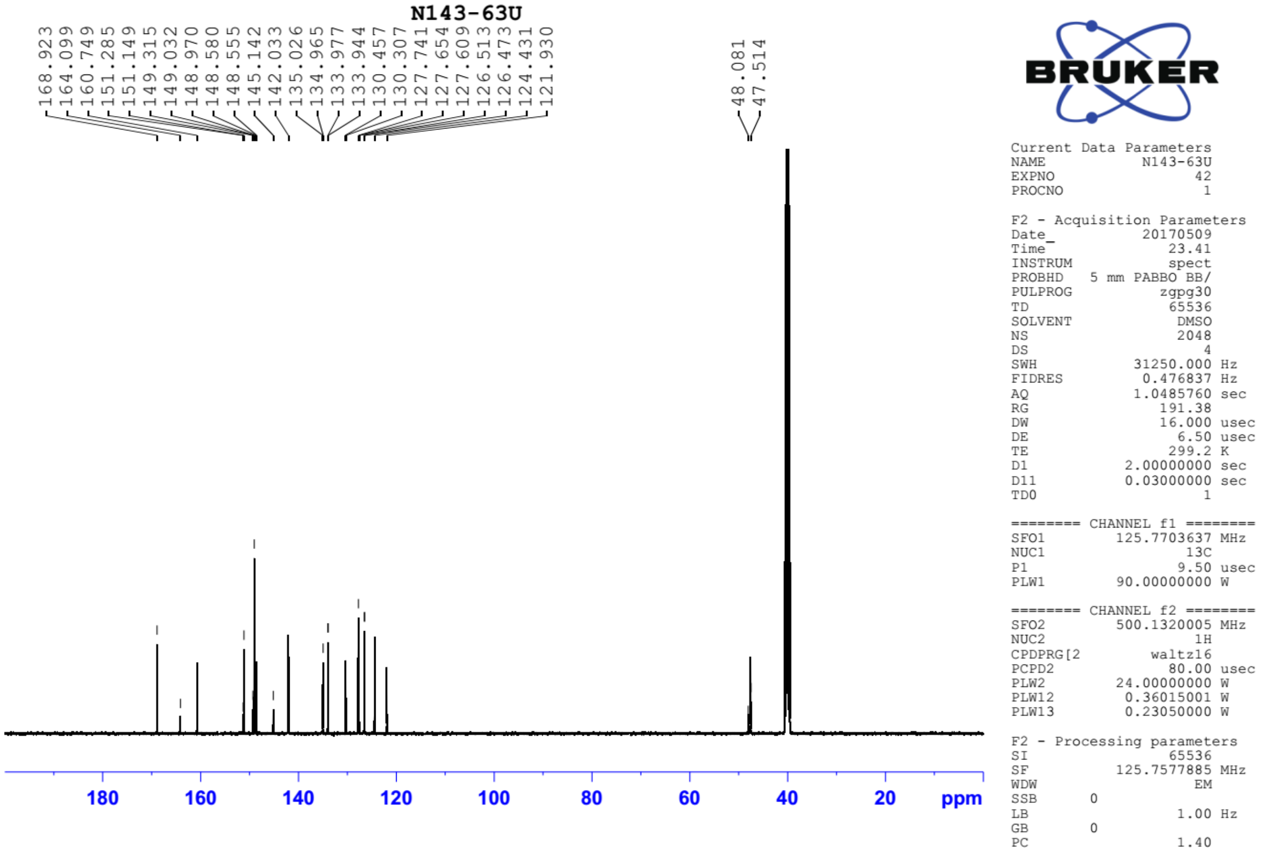

^{1}H NMR of compound 6f

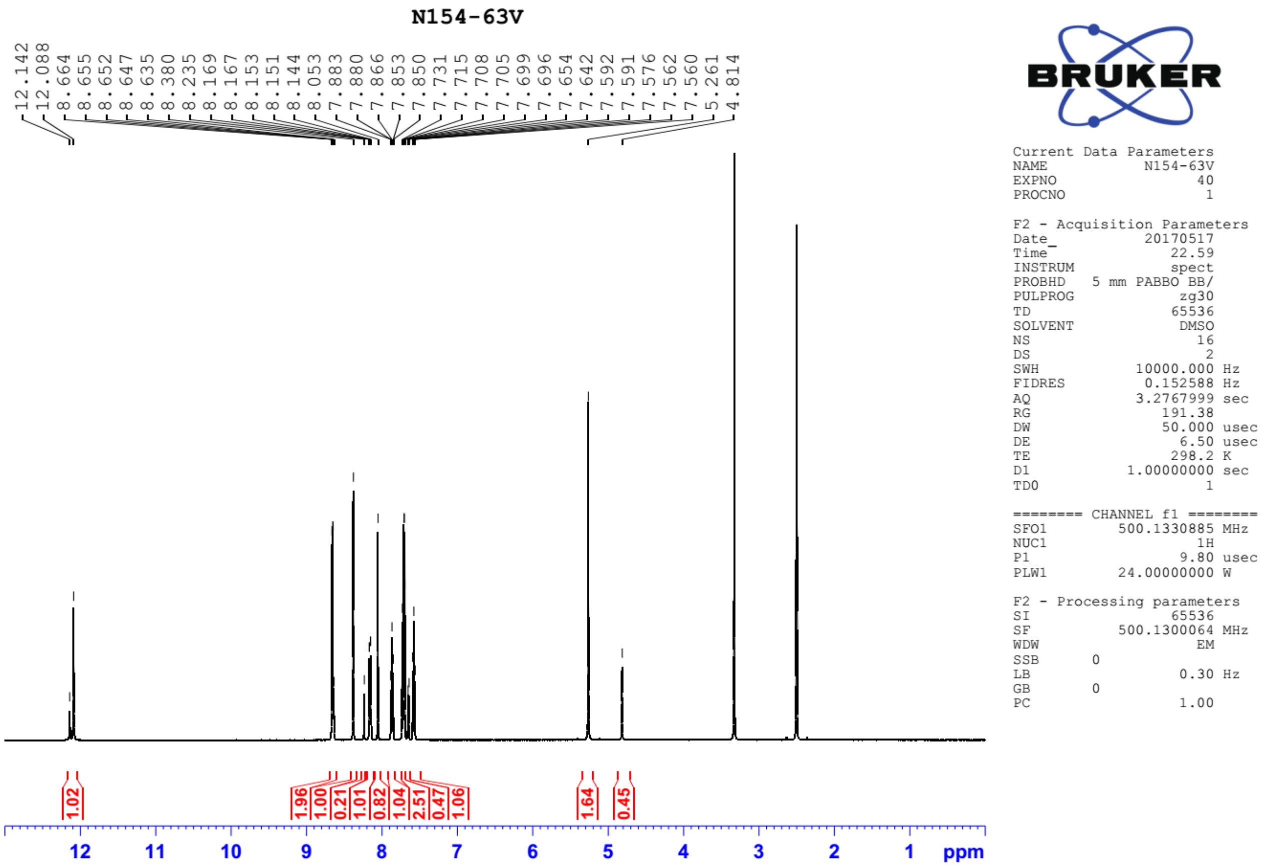

^{13}C NMR of compound 6f

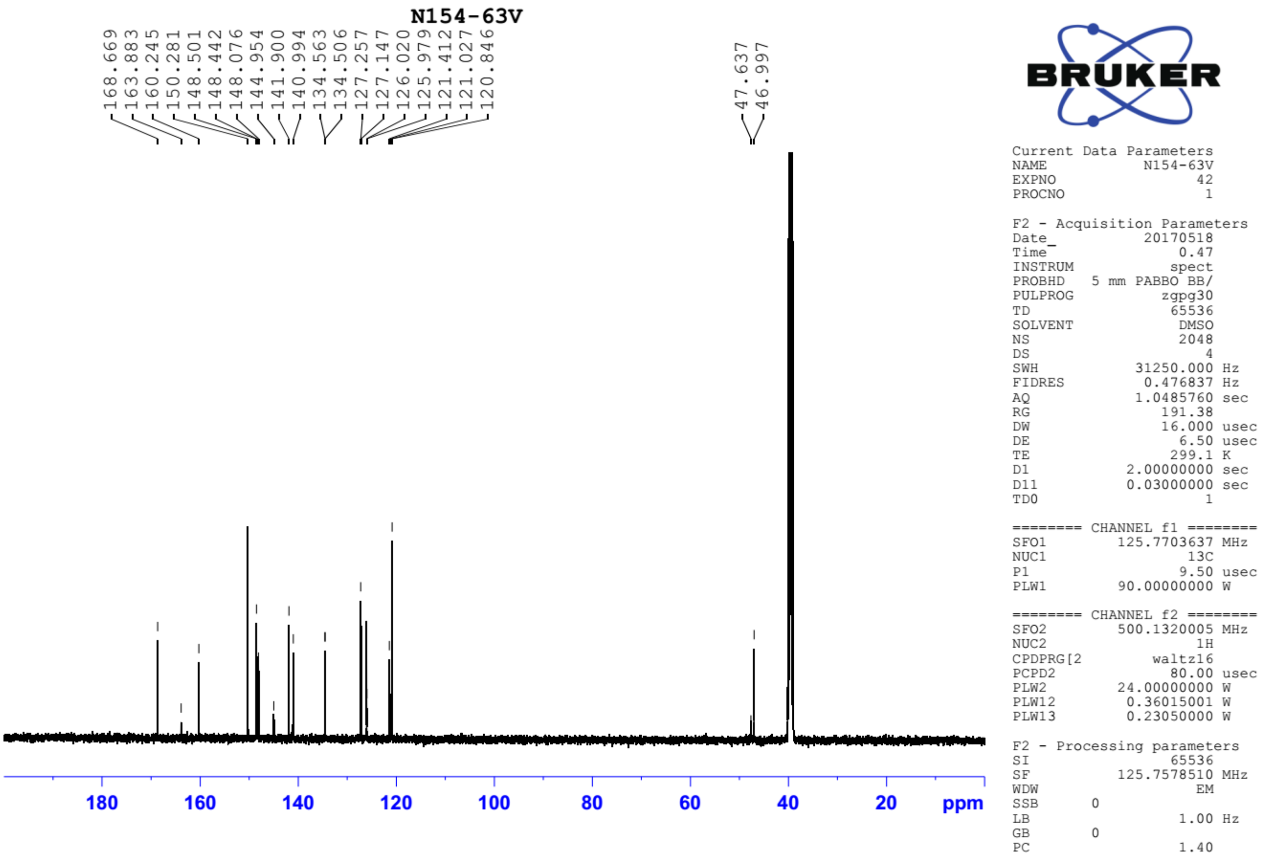

^{1}H NMR of compound 6g

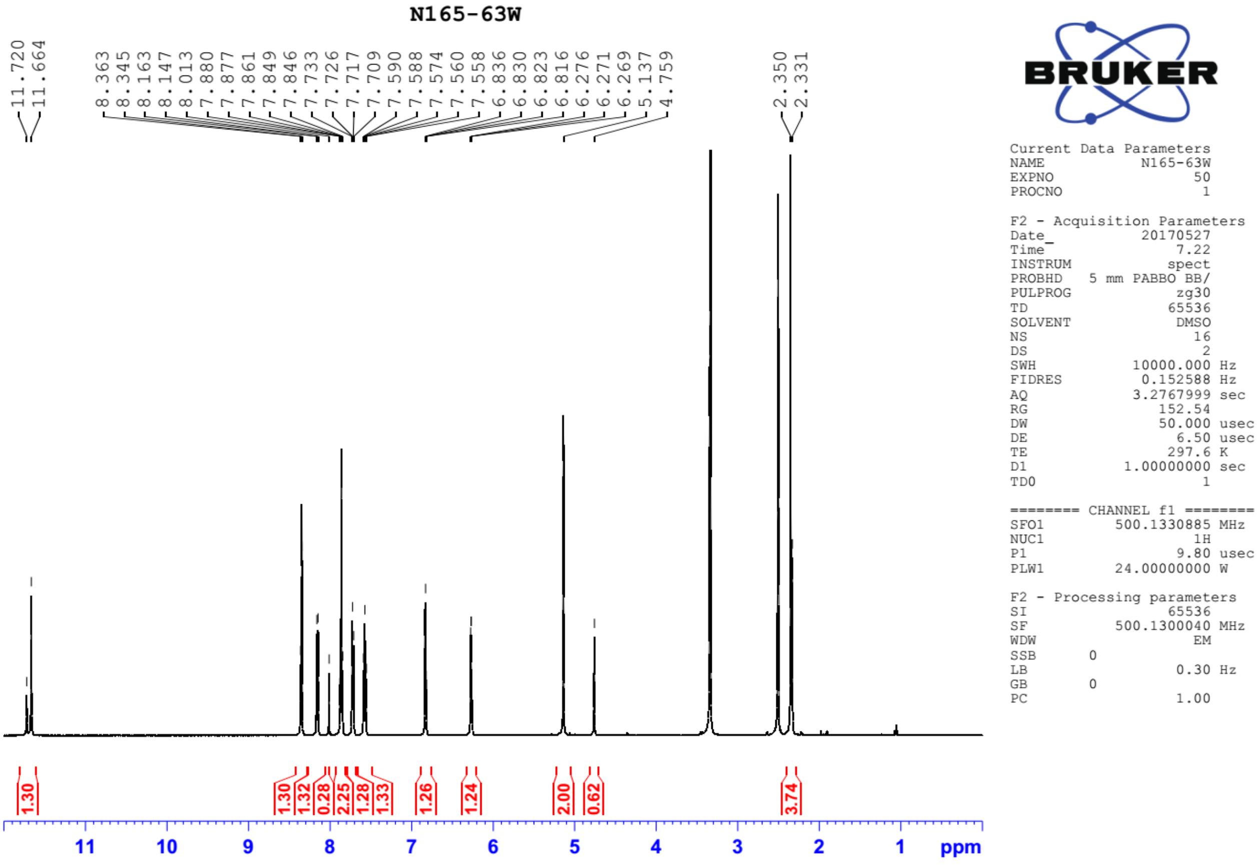

^{13}C NMR of compound 6g

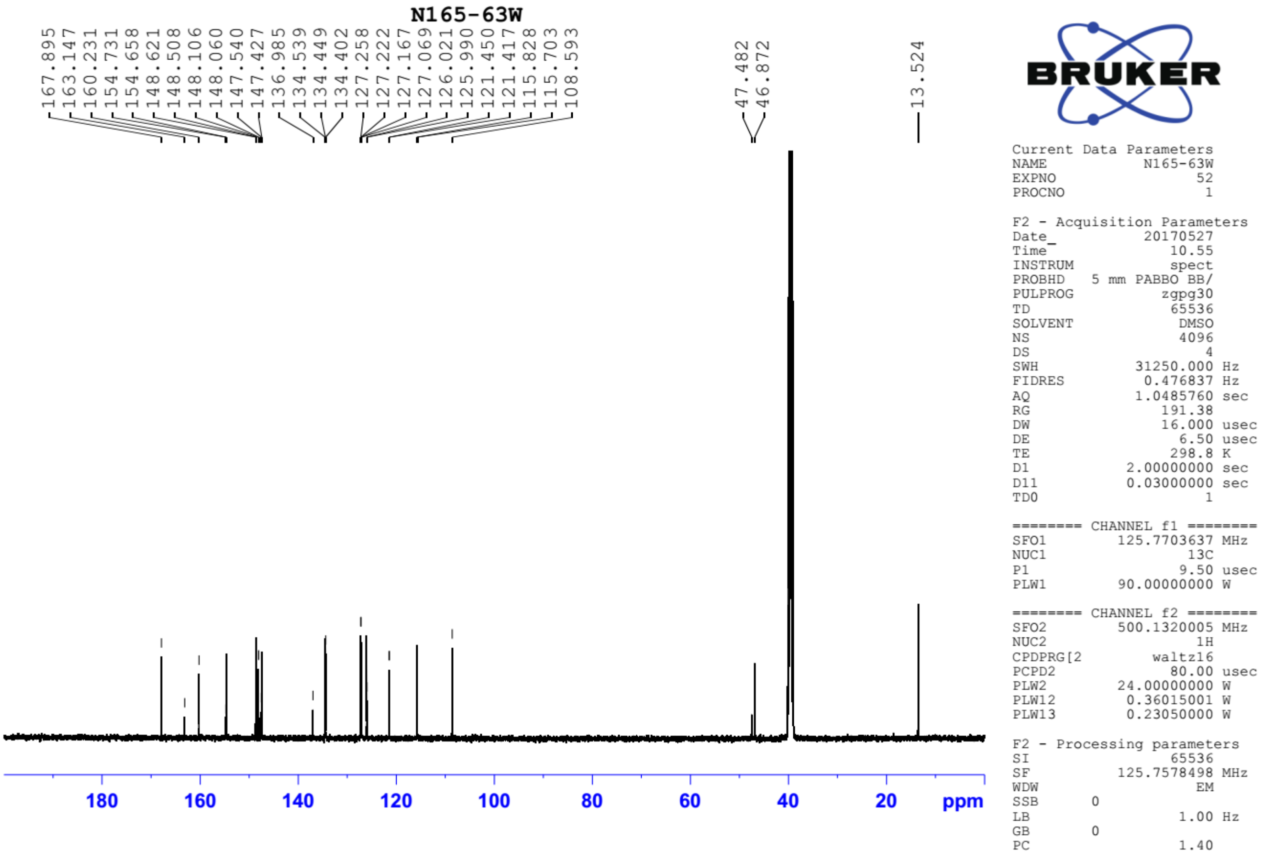

^{1}H NMR of compound 7a

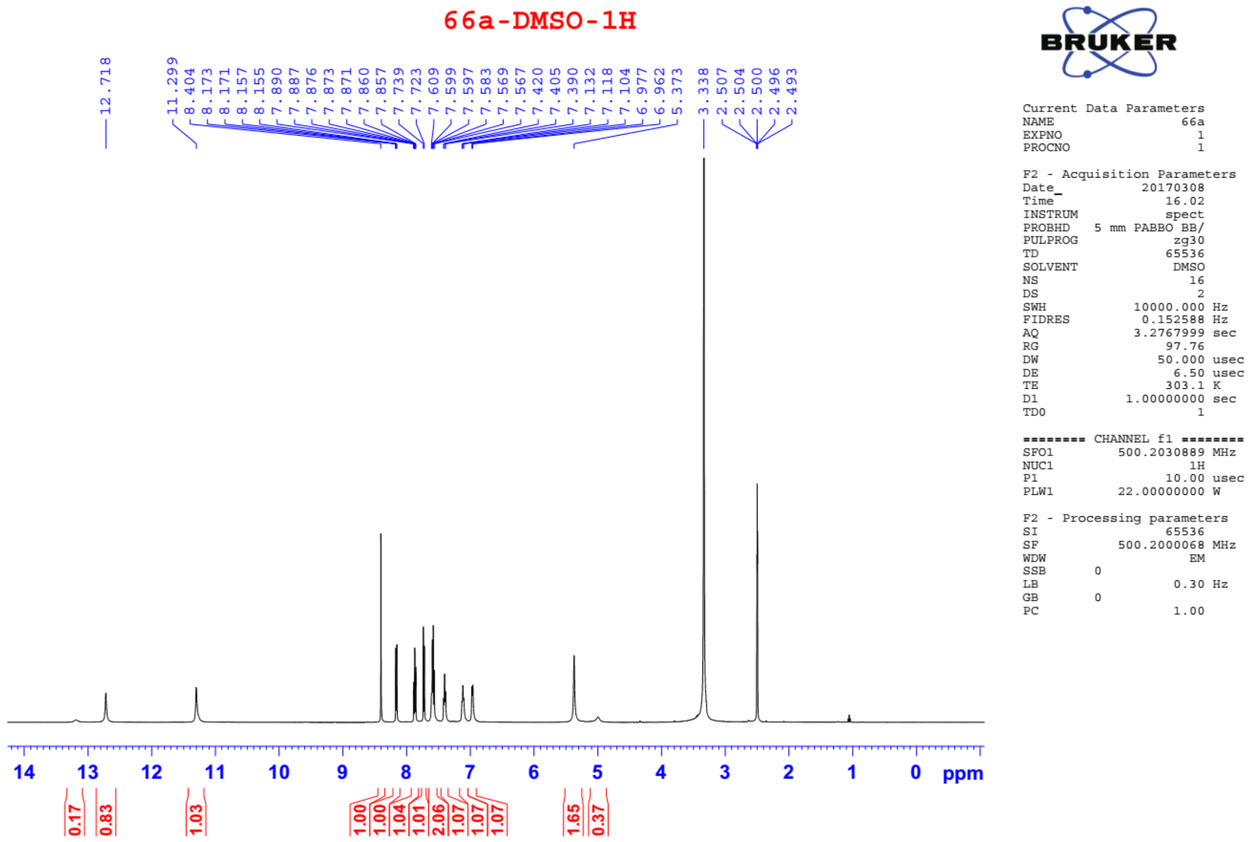

^{13}C NMR of compound 7a

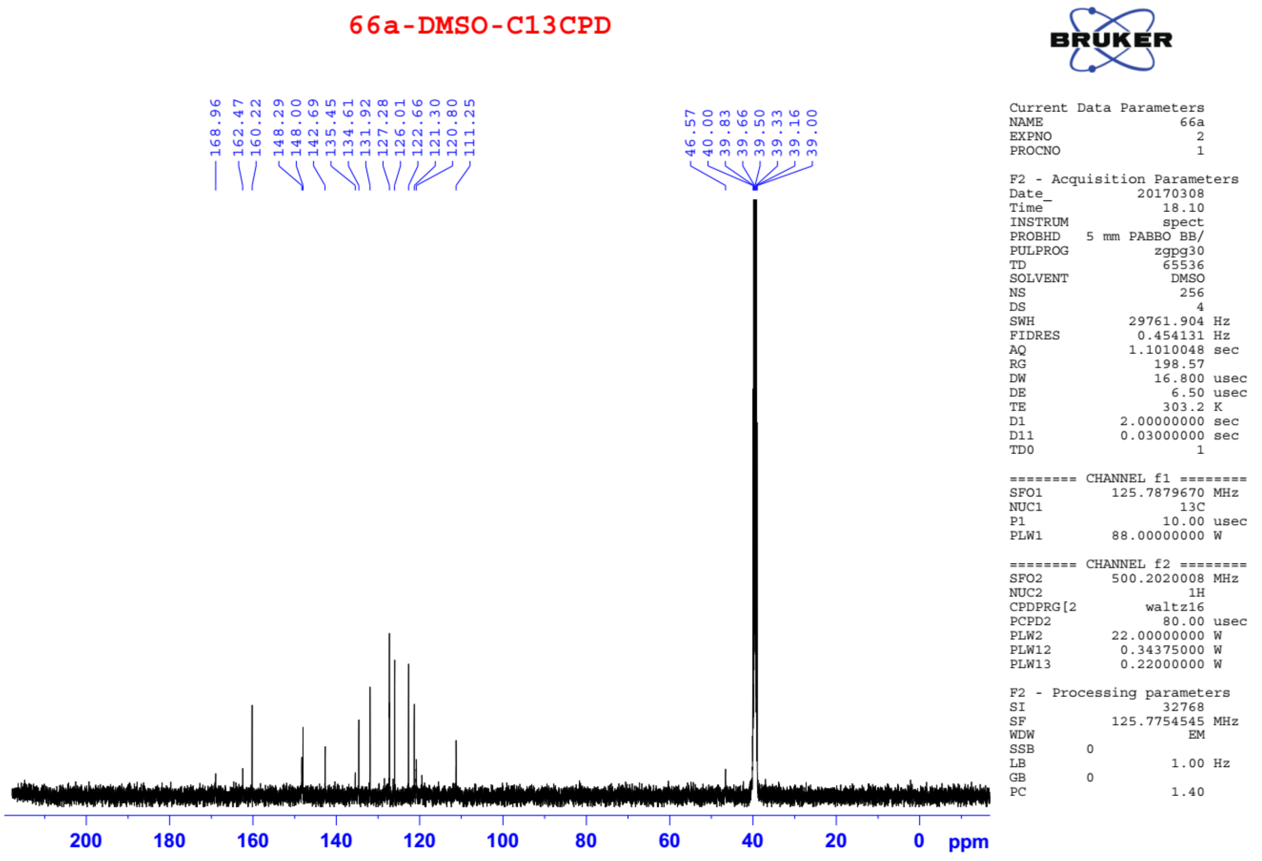

^{1}H NMR of compound 7b

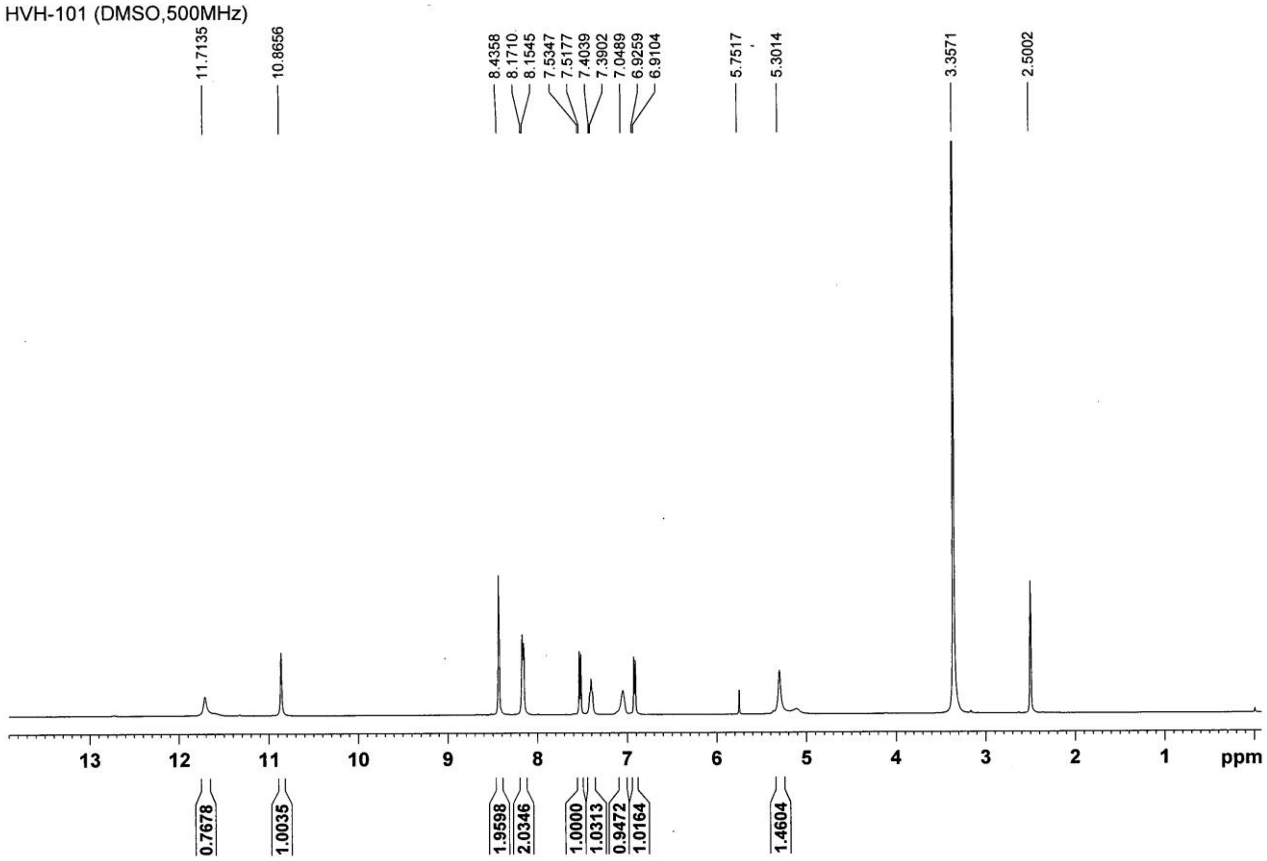

^{13}C NMR of compound 7b

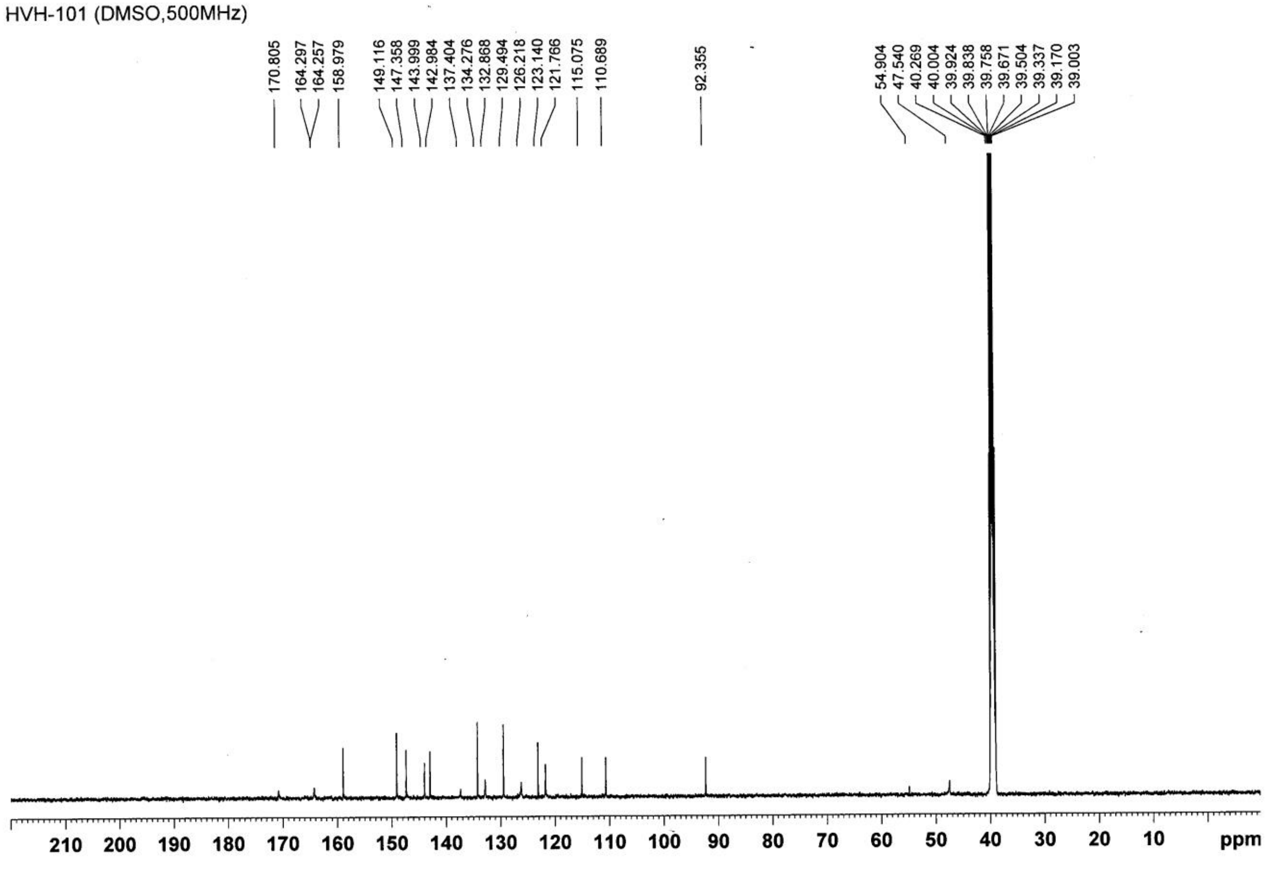

Caption for the Graphical Abstract

A series of (E)-N'-arylidene-2-(4-oxoquinazolin-4(3H)-yl)acetohydrazides with significant cytotoxicity against three human cancer cell lines. Compound 5t displayed cytotoxicity up to 5-fold more potent than 5-FU, meanwile compound 7b activated caspases activity by almost 200% in comparison to that of PAC-1.
